# Dynamic analysis and spatiotemporal evolution of multi-stage knowledge dissemination under AI-enabled dual-channel “Interpersonal–Human-Machine” interaction

**DOI:** 10.1371/journal.pone.0361482

**Published:** 2026-10-29

**Authors:** Yanhua Lv, Yadong Wang, Huanhuan Wang, Sida Kang

**Affiliations:** School of Business Administration, University of Science and Technology Liaoning, Anshan, Liaoning, China; Institute of Theoretical and Applied Informatics Polish Academy of Sciences: Instytut Informatyki Teoretycznej i Stosowanej Polskiej Akademii Nauk, UKRAINE

## Abstract

With the deep integration of digitalization and artificial intelligence technologies into enterprise knowledge management, AI-assisted learning is reshaping the channels through which knowledge is acquired within organizations. Meanwhile, knowledge dissemination is evolving from traditional interpersonal interaction toward human-machine collaborative interaction. In light of this transformation, this study develops a multi-stage 2*SEIR*-*CA* dynamic model of knowledge dissemination under an AI-enabled mechanism that incorporates dual-channel “interpersonal–human-machine” interaction. Specifically, employees are classified into five states: employees who have not yet mastered the knowledge, employees with limited knowledge mastery, employees with strong knowledge mastery, knowledge-disseminating employees, and knowledge-exited employees. On this basis, the next-generation matrix method is employed to calculate the basic reproduction number of the system and to analyze the existence and stability of equilibrium points. An optimal control model is then established to derive strategies for promoting knowledge dissemination, and a cellular automaton model is introduced to characterize its spatiotemporal evolution. The results show that the system exhibits a pronounced threshold effect: when key dissemination parameters exceed their critical levels, knowledge can continue to diffuse within the organization; otherwise, it will gradually decay. Second, the AI-enabled mechanism enhances knowledge transformation efficiency and contact intensity, thereby significantly promoting the transition of employees with strong knowledge mastery to knowledge-disseminating employees. Third, the optimal control strategy proposed in this study can optimize transformation pathways across different stages under resource constraints, thereby improving overall dissemination efficiency and maintaining the structural stability of the dissemination system. Fourth, the spatiotemporal simulations demonstrate that, under optimal regulation, knowledge dissemination can form a stable diffusion pattern with high coverage. Under the assumptions and parameter settings of the proposed model, the findings provide theoretical insights into the dynamic mechanisms underlying AI-enabled knowledge dissemination and offer model-based implications for knowledge management practices in organizations.

## Introduction

Against the backdrop of the accelerated advancement of the digital economy and intelligent transformation, knowledge has become a core strategic resource driving organizational innovation and social development [1]. The creation, sharing, and dissemination of knowledge constitute not only an essential foundation for organizational learning but also a core driver through which enterprises acquire sustained competitive advantage [2,3]. Early research on knowledge management has shown that knowledge does not reside solely at the individual level but is deeply embedded in organizational processes, technological tools, and social interactions [4]. Therefore, the efficiency of knowledge dissemination among individuals, teams, and organizations has become a key factor in determining organizational performance [5–7].

The study by Argote et al. confirms that performance differences among organizations stem, to a considerable extent, from variations in the efficiency of knowledge transfer and dissemination [8]. Wang et al.’s systematic review of the knowledge-sharing literature further indicates that knowledge dissemination is jointly shaped by multiple factors, including individual motivation, organizational culture, technological platforms, and social network structure [9]. In addition, information technology infrastructure and the spatiotemporal configuration of organizations also exert a significant influence on the efficiency of knowledge dissemination. For instance, the application of IT technologies and technology co-location patterns can effectively promote knowledge sharing and diffusion within enterprises [10]. Knowledge dissemination is therefore not merely a process of information exchange but a complex social phenomenon shaped by individual interactions and technological environments.

Against this backdrop, the rapid development of digital technologies and Internet platforms is profoundly reshaping the mode of knowledge dissemination [11]. Digital platforms, social media, and online collaboration technologies have expanded the scale and speed of knowledge exchange, enabling knowledge to disseminate rapidly across organizational and geographical boundaries [12]. Digital information technologies not only improve the efficiency of knowledge acquisition, but also promote collaboration among different actors, thereby giving rise to a more open knowledge dissemination network [13]. Meanwhile, the development of digital technologies has endowed knowledge flows with pronounced networked and decentralized characteristics, generating complex knowledge dissemination networks and giving knowledge dissemination the evolutionary features of complex networks [14]. As technological evolution continues to advance, this process is being further shaped by emerging intelligent technologies.

In recent years, the rapid development of artificial intelligence (AI) has fundamentally reconfigured the modes of knowledge generation, dissemination, and organizational decision-making [15]. AI systems can process large-scale data and support complex cognitive activities. Their application can not only automate decision-making processes but also enhance human cognitive capabilities, thereby giving rise to a management mode in which automation and augmentation coexist [16,17]. Raisch et al. define this phenomenon as the “automation–augmentation paradox” and point out that organizations must strike a balance between automation efficiency and human cognitive capability when applying AI [18]. More importantly, AI-driven decision systems can reduce information asymmetry through data analysis and improve information-processing efficiency, thereby substantially accelerating knowledge generation and dissemination [19]. Today, AI has moved beyond the role of a traditional auxiliary tool and has become deeply embedded in knowledge dissemination networks as a heterogeneous node, significantly altering the topological structure and evolutionary dynamic characteristics of knowledge dissemination [20,21]. Accordingly, the process of knowledge dissemination exhibits increasingly complex patterns of dynamic evolution, providing a foundation for subsequent dynamic modeling and quantitative analysis.

To accurately characterize the dynamic evolution mechanism of knowledge dissemination, scholars have widely drawn upon the theoretical analytical framework of epidemic dissemination models [22]. Knowledge diffusion and disease transmission are highly similar in structure, as both involve the continuous diffusion of information or knowledge within a population through contact among individuals [23]. The classical *SIR* model proposed by Kermack and McKendrick laid a solid mathematical foundation for describing dynamic transmission processes in populations [24]. On this basis, researchers further developed extended models such as *SEIR* and *SIS* to characterize more complex dissemination mechanisms involving latent periods, reinfection, and other processes [25,26]. Anderson et al. systematized the theory of epidemic dynamic dissemination and proposed the basic reproduction number as a core threshold for determining whether dissemination will break out [27]. These theoretical models have not only been widely applied in epidemiological research, but have also gradually been introduced into research fields such as information dissemination, innovation diffusion, and knowledge dissemination [28–30].

Classical dissemination models remain limited in characterizing spatiotemporal heterogeneity and local interactions because they primarily rely on continuous differential equations to describe macro-level population changes and therefore have difficulty capturing complex local interactions among individuals in real-world systems [31]. Although complex network theory has become an important tool for studying dissemination processes, it still lacks the capacity for microscopic simulation of the spatiotemporal evolution of dissemination behaviors [32]. In AI-enabled knowledge networks, knowledge dissemination depends heavily on specific organizational spatiotemporal configurations and local interactions on digital platforms, while the spatiotemporal heterogeneity of network structure can significantly alter dissemination pathways [33]. To this end, cellular automata (*CA*), as an effective tool for characterizing discrete spatiotemporal dynamic evolution, have been widely introduced into the study of dissemination behaviors [34,35]. Cellular automata are discrete dynamical systems composed of a large number of cells, each of which updates its state according to simple rules based on the states of its neighborhood. Although these rules are concise, they can generate highly complex self-organizing structures and accurately simulate macro-level spatiotemporal diffusion processes through underlying local neighborhood interaction rules [36]. This characteristic enables cellular automata to effectively compensate for the dual limitations of traditional macro-level differential equations and complex network models in characterizing spatiotemporal heterogeneity, thereby providing a well-suited methodological framework for simulating the spatiotemporal evolution of AI-enabled knowledge dissemination.

After clarifying the spatiotemporal evolution mechanism of knowledge dissemination, how to scientifically regulate this process has become a core practical demand of organizational knowledge management in the digital era. Against this backdrop, optimal control theory provides important methodological support for designing effective intervention strategies for dissemination processes [37,38]. This method was first applied to epidemic models, where control variables were introduced to reduce the scale of infection and regulate the dissemination process [39,40]. Studies have shown that optimal control strategies can be solved by means of Pontryagin’s Maximum Principle, thereby minimizing control costs while reducing the number of infected individuals [41]. Such methods have also been applied to research on information dissemination and cybersecurity, including scenarios such as malware dissemination control and the regulation of information diffusion processes [42,43]. These applications confirm that optimal control theory is an effective tool for regulating dissemination processes in complex systems. In the context of AI-enabled knowledge dissemination, organizations can dynamically regulate the processes of knowledge acquisition, transformation, and diffusion by improving AI platform support and incentive mechanisms and optimizing the allocation of knowledge dissemination resources, thereby balancing dissemination effectiveness against intervention costs under resource constraints.

In summary, existing studies have laid a solid theoretical foundation for understanding the dynamic process and control methods of knowledge dissemination; however, research on AI-enabled knowledge dissemination and its spatiotemporal evolution remains relatively scarce. In studies of AI-enabled knowledge dissemination, traditional single-channel dissemination models are typically based on the assumption of homogeneous interpersonal interaction and further presume homogeneous mixing among individuals, whereby all individuals have the same contact probability [44]. Such models cannot fully reflect the complex dissemination mechanism in an AI-enabled environment. In the context of human-machine collaboration, knowledge dissemination largely occurs within a dual-layer network structure formed by the coupling of the “employee–employee” relational network and the “employee–AI” interaction network. One layer describes direct knowledge dissemination among individuals, while the other characterizes the process by which individuals acquire knowledge and enhance their capabilities with support from AI-assisted learning systems. Accordingly, knowledge-state transitions exhibit multidimensional characteristics [45]. Existing research on the spatiotemporal evolution of knowledge dissemination has focused primarily on its temporal dynamic evolution, with relatively limited attention paid to spatiotemporal structural factors.

Based on the above considerations, this study constructs a multi-stage 2*SEIR*-*CA* dynamic model of knowledge dissemination that integrates a dual-channel “interpersonal–human-machine” interaction mechanism. Compared with existing knowledge dissemination models, the proposed model has three major improvements. First, the $S_{1}-S_{2}-E-I-R$ multi-stage state structure explicitly captures heterogeneity in employees’ initial knowledge mastery and the progressive transition from knowledge contact, comprehension, and internalization to active dissemination. Second, interpersonal knowledge contact and AI-assisted learning are incorporated into a unified state-transition framework, respectively characterizing the knowledge-access effect generated through interpersonal interaction and the capability-enhancement effect arising from AI-assisted learning. Third, under a unified representation of knowledge states and transition mechanisms, the macroscopic continuous dynamic relationships are mapped onto local neighborhood interactions and integrated with threshold analysis and optimal control, thereby enabling the enterprise knowledge dissemination process in an AI-enabled context to be examined from three complementary perspectives: dynamic evolution, regulation, and spatiotemporal diffusion.

The rest of this paper is organized as follows. The multi-stage 2*SEIR*-*CA* dynamic model of knowledge dissemination under an AI-enabled mechanism, which considers dual-channel “interpersonal–human-machine” interactions, is presented in the “The Model” section. The model assumptions and parameters are also described, and the cellular automata method is introduced to characterize the spatiotemporal evolution mechanism of knowledge dissemination. The basic reproduction number of the system, calculated using the next-generation matrix method, together with the local and global stability of the knowledge-free equilibrium and the knowledge-present equilibrium, is presented in the “Stability Analysis of the Model” section. The optimal control system for knowledge dissemination and the corresponding optimal control strategies are presented in the “The Optimal Control Model” section. The influence of changes in key parameters on the knowledge dissemination process, as well as the effectiveness of the optimal control strategies based on numerical simulations, is illustrated in the “Numerical Simulations” section. In addition, the spatiotemporal evolution characteristics of knowledge dissemination are further explored using the cellular automata method. Finally, the “Conclusions and Implications” section summarizes the research work of this paper and presents corresponding managerial implications and future research directions.

## The model

### Model construction

To characterize the dynamic evolution of enterprise knowledge dissemination in an AI-enabled environment, this study constructs a multi-stage 2*SEIR*-*CA* dynamic model that integrates a dual-channel “interpersonal–human-machine” interaction mechanism. Unlike traditional dissemination frameworks, where state transitions are mainly driven by interpersonal knowledge contact, the proposed model considers heterogeneity in employees’ initial knowledge mastery. It also captures the stagewise progression of knowledge from contact and comprehension to internalization and active dissemination. Meanwhile, the knowledge dissemination process considered in this study involves multiple stages, including learning, comprehension, and internalization, and individuals can further participate in knowledge dissemination only after they have mastered the knowledge. Specifically, interpersonal interaction determines employees’ opportunities to come into contact with relevant knowledge, whereas AI is not introduced as an independent dissemination agent or an additional population compartment in the model. Instead, it is modeled as an enabling mechanism embedded in the knowledge-learning process, influencing the efficiency with which employees with different levels of prior knowledge comprehend and master knowledge. Accordingly, this study models “knowledge access” and “learning capability enhancement” as two functionally distinct yet mutually coupled mechanisms, thereby characterizing the dynamic process through which employees progressively transition from knowledge learners to knowledge-disseminating employees under AI-assisted learning. On this basis, the following basic assumptions are proposed.

Consider an organizational system composed of enterprise employees, in which knowledge is disseminated among employees through communication, collaboration, and learning. According to employees’ degree of mastery of a certain professional knowledge and their roles in the knowledge dissemination process, the members of the system are classified into five distinct types: (1) employees who have not yet mastered the knowledge, denoted by $S_{1}\left(t\right)$, referring to individuals who have not yet been exposed to it or who lack an adequate understanding of it; (2) employees with limited knowledge mastery, denoted by $S_{2}\left(t\right)$, referring to individuals who have already been exposed to the relevant knowledge but possess only a preliminary understanding of it; (3) employees with strong knowledge mastery, denoted by E(t), referring to individuals who have a relatively in-depth understanding of the knowledge but have not yet actively participated in its dissemination; (4) knowledge-disseminating employees, denoted by I(t), referring to individuals who have mastered the knowledge and actively disseminate it to other members; (5) knowledge-exited employees, denoted by R(t), referring to individuals who have lost interest in the knowledge or regard it as no longer valuable, and therefore no longer participate in knowledge dissemination.

The descriptions of the model compartments are presented in Table 1.

**Table 1:** Description of the compartments in the 2*SEIR*-*CA* model.

Parameter	Meaning
$S_{1}\left(t\right)$	Employees who have not yet mastered the knowledge.
$S_{2}\left(t\right)$	Employees with limited knowledge mastery.
E(t)	Employees with strong knowledge mastery.
I(t)	Knowledge-disseminating employees.
R(t)	Knowledge-exited employees.

Based on the above compartmental classification, the model further characterizes employee inflow and outflow, knowledge contact, AI-assisted learning, and the transition processes among different knowledge states through relevant parameters. The specific meanings of these parameters are described as follows. *B* represents the number of employees entering the organizational system per unit time, and *p* denotes the proportion of newly recruited employees who have not yet mastered the knowledge. Therefore, *pB* and (1−p)B represent the numbers of employees entering the *S*_1_ and *S*_2_ states, respectively. The parameter μ represents the rate at which employees leave the organizational system due to resignation, job transfer, or other natural causes. The parameter α represents the rate of effective knowledge contact between employees and knowledge-disseminating employees, while *k* denotes the effective connectivity degree of the organizational knowledge network. Together, these two parameters characterize the intensity of knowledge contact and coverage within the interpersonal interaction channel. To characterize the facilitating role of AI-assisted learning in employees’ knowledge acquisition and comprehension processes, an AI-enabled mechanism is introduced in this study. Specifically, $\varphi_{1}^{AI}$ and $\varphi_{2}^{AI}$ are used to represent the efficiencies at which employees in the *S*_1_ and *S*_2_ states, respectively, transform into employees with a higher level of knowledge mastery under the influence of AI-assisted learning. Meanwhile, η and ρ represent the proportions of employees in the *S*_1_ and *S*_2_ states, respectively, who withdraw from the current knowledge dissemination process after knowledge exposure due to factors such as a lack of interest, insufficient knowledge adaptability, or decreased learning willingness. In the macroscopic dynamic model, employee interactions with AI tools encompass substantial micro-level heterogeneity, including AI tool accessibility, frequency of use, feedback relevance, and employees’ degree of adoption of AI-generated recommendations. To preserve the analytical tractability of the model, this study adopts an aggregate representation in which the average enabling effects of these heterogeneous human-machine interactions are captured by $\varphi_{1}^{AI}$ and $\varphi_{2}^{AI}$, respectively representing the extent to which AI facilitates the comprehension and mastery of knowledge among employees who have not yet mastered the knowledge and those with limited knowledge mastery. The parameter ι represents the rate at which employees with a relatively high level of knowledge mastery transform into knowledge disseminators after completing knowledge absorption, comprehension, and internalization. The parameter σ denotes the rate at which such employees withdraw from the current knowledge dissemination process before participating in knowledge dissemination. The parameter β represents the rate at which knowledge disseminators transform into knowledge exiters due to factors such as knowledge updating, weakened willingness to disseminate knowledge, or changes in work tasks. The aforementioned parameters collectively characterize the dynamic evolutionary mechanism of the organizational knowledge dissemination system from multiple perspectives, including organizational member mobility, knowledge contact, AI-assisted learning, knowledge absorption and transformation, as well as the attenuation of dissemination behaviors.

The meanings, operational definitions, and value ranges of the model parameters are presented in Table 2.

**Table 2:** Baseline values, explored ranges, and justification of the parameters in the 2*SEIR*–*CA* model.

Parameter	Meaning	Baseline value	Parameter ranges	Source or justification
*B*	Number of employees entering the organizational system per unit time.	3	[2,6]	Organization-size scenario parameter. Together with the natural outflow rate μ, it determines the long-run population scale B/μ. The explored range covers low, moderate, and relatively high employee-inflow scenarios.
*p*	Proportion of newly entering employees initially assigned to state *S*_1_; 1−p denotes the proportion initially assigned to state *S*_2_.	0.50	[0.10,0.90]	Definition-constrained parameter with a theoretical domain of [0,1]. The baseline value represents an equal allocation of newly entering employees between the *S*_1_ and *S*_2_ states.
μ	Natural outflow rate.	0.20	[0.05,0.40]	Duration-informed scenario parameter. Its reciprocal 1/μ represents the expected organizational residence-time scale.
α	Knowledge contact rate.	0.70	[0.30,1.00]	Mechanism-based scenario parameter. The baseline value represents a moderately high level of effective knowledge contact, whereas the explored range covers low- to high-contact conditions.
*k*	Network average degree.	1.50	[0.50,3.00]	Network-structure scenario parameter. The baseline value represents moderate effective connectivity, and the explored range covers relatively sparse to relatively dense organizational connections.
$\varphi_{1}^{AI}$	Transformation rate at which employees who have not yet mastered the knowledge transform into employees with strong knowledge mastery under AI-assisted learning.	0.70	[0.40,1.00]	Mechanism-based scenario parameter. The baseline scenario assumes that AI provides relatively strong marginal assistance to employees with weaker prior knowledge; this assumption is relaxed over the full explored range in the robustness analysis.
$\varphi_{2}^{AI}$	Transformation rate at which employees with limited knowledge mastery transform into employees with strong knowledge mastery under AI-assisted learning.	0.50	[0.20,0.80]	Mechanism-based scenario parameter. A baseline value different from that of $\varphi_{1}^{AI}$ is used to represent heterogeneous AI-assisted transformation efficiencies across employees with different initial knowledge foundations.
η	Proportion of employees who have not yet mastered the knowledge transforming into knowledge-exited employees after contact with knowledge-disseminating employees.	0.20	[0.10,0.90]	Definition-constrained conditional-proportion parameter with a theoretical domain of [0,1]. The baseline value represents a relatively low post-contact withdrawal level.
ρ	Proportion of employees with limited knowledge mastery transforming into knowledge-exited employees after contact with knowledge-disseminating employees.	0.20	[0.10,0.90]	Definition-constrained conditional-proportion parameter. The same baseline value as η is used to construct a symmetric baseline withdrawal scenario, while their potentially different effects are examined through parameter perturbation.
ι	Knowledge absorption rate.	0.70	[0.30,1.00]	Duration-informed mechanism-based parameter. The baseline value represents a relatively efficient transformation from strong knowledge mastery to active knowledge dissemination.
σ	Proportion of employees with strong knowledge mastery transforming into knowledge-exited employees.	0.20	[0.10,0.50]	State-exit scenario parameter representing withdrawal caused by interrupted learning, declining knowledge relevance, or failure to develop a willingness to disseminate knowledge.
β	Proportion of knowledge-disseminating employees transforming into knowledge-exited employees due to their own reasons.	0.10	[0.05,0.50]	State-exit scenario parameter. The baseline value is lower than the E→I transition rate, representing a baseline condition in which knowledge-dissemination behavior has a certain degree of persistence.

It should be noted that this study focuses on mechanism-oriented theoretical modeling. At present, longitudinal micro-level enterprise data capable of simultaneously tracking the five knowledge states of employees and their complete state transition processes are still unavailable. Therefore, the baseline parameter values in Table 2 should not be interpreted as empirical coefficients statistically estimated from a specific enterprise; rather, they should be regarded as benchmark settings adopted to construct representative knowledge dissemination scenarios within a theoretically feasible range. Specifically, the proportion-based parameters are constrained by their definitions to fall within the range of [0,1]. For transformation-rate parameters, including knowledge absorption rates, state-exit rates, and natural employee outflow rates, their rationality is assessed based on the average duration of the corresponding states implied by the reciprocal values of these parameters. For the AI-assisted learning parameters and organizational network parameters, for which directly transferable empirical estimates are currently unavailable, this study explicitly treats them as mechanism-oriented scenario parameters. Subsequently, systematic perturbation analyses are conducted to examine whether the main findings remain robust under variations in these baseline parameter settings.

Within each unit time, *B* individuals enter the system, where *B* is a constant. It is assumed that *pB* and (1−p)B individuals enter the group of employees who have not yet mastered the knowledge and the group of employees with limited knowledge mastery, respectively. Considering natural employee outflow from the organizational system, and to simplify the model, all groups are temporarily assumed to have the same natural employee outflow rate μ; this assumption may be relaxed in future research.

As shown in Fig. 1, the state transitions of employees who have not yet mastered the knowledge, employees with limited knowledge mastery, employees with strong knowledge mastery, knowledge-disseminating employees, and knowledge-exited employees within a unit time are presented as follows:

- Within a unit time, employees who have not yet mastered the knowledge may come into contact with knowledge-disseminating employees. Through AI-assisted learning, they may transform into employees with strong knowledge mastery, with the corresponding transition quantity given by $\alpha \varphi_{1}^{AI}kS_{1}I$. Alternatively, they may directly transform into knowledge-exited employees, with the corresponding transition quantity given by $\alpha \eta S_{1}I$. Meanwhile, employees in this category leave the organizational system due to natural outflow, with the corresponding number given by $\mu S_{1}$.Considering the number of newly entering employees *pB*, the change in the number of employees who have not yet mastered the knowledge within the organizational system per unit time is expressed as $pB-\alpha \eta S_{1}I-\alpha \varphi_{1}^{AI}kS_{1}I-\mu S_{1}$.
- After employees with limited knowledge mastery come into contact with knowledge-disseminating employees, they may likewise transform into employees with strong knowledge mastery through AI-assisted learning, with the corresponding transition quantity given by $\alpha \varphi_{2}^{AI}kS_{2}I$. Alternatively, they may transform into knowledge-exited employees, with the corresponding transition quantity given by $\alpha \rho S_{2}I$. Meanwhile, employees in this category leave the organizational system due to natural outflow, with the corresponding number given by $\mu S_{2}$.Considering the number of newly entering employees (1−p)B, the change in the number of employees with limited knowledge mastery per unit time is expressed as $\left(1-p\right)B-\alpha \rho S_{2}I-\alpha \varphi_{2}^{AI}kS_{2}I-\mu S_{2}$.
- Employees in the state of strong knowledge mastery are mainly derived from the transformation of employees in the *S*_1_and *S*_2_ states under knowledge contact and AI-assisted learning. The corresponding inflows are $\alpha \varphi_{1}^{AI}kS_{1}I$ and $\alpha \varphi_{2}^{AI}kS_{2}I$. Employees in this category can further transform into knowledge-disseminating employees or knowledge-exited employees, and may also leave the organizational system due to natural outflow. Therefore, the change in the number of employees with strong knowledge mastery per unit time is expressed as $\alpha \varphi_{1}^{AI}kS_{1}I+\alpha \varphi_{2}^{AI}kS_{2}I-\left(\sigma +\iota +\mu \right)E$.
- Knowledge-disseminating employees are mainly transformed from employees with strong knowledge mastery, with the corresponding inflow given by ιE. Meanwhile, they may transform into knowledge-exited employees with probability β, and the number of employees leaving the organizational system is μI. Therefore, the change in the number of knowledge-disseminating employees per unit time is expressed as ιE−(β+μ)I.
- Through the above analysis, the inflows of knowledge-exited employees originate from the exits of employees in the *S*_1_ state, the *S*_2_ state, the *E* state, and knowledge-disseminating employees in the *I* state. The corresponding inflow quantities are $\alpha \eta S_{1}I$, $\alpha \rho S_{2}I$, σE, and βI, respectively. Meanwhile, some knowledge-exited employees leave the organizational system due to certain reasons, and the corresponding number of employees leaving is μR. Therefore, the change in the number of knowledge-exited employees *R* per unit time is expressed as $\alpha \eta S_{1}I+\alpha \rho S_{2}I+\sigma E+\beta I-\mu R$.

**Fig 1:**
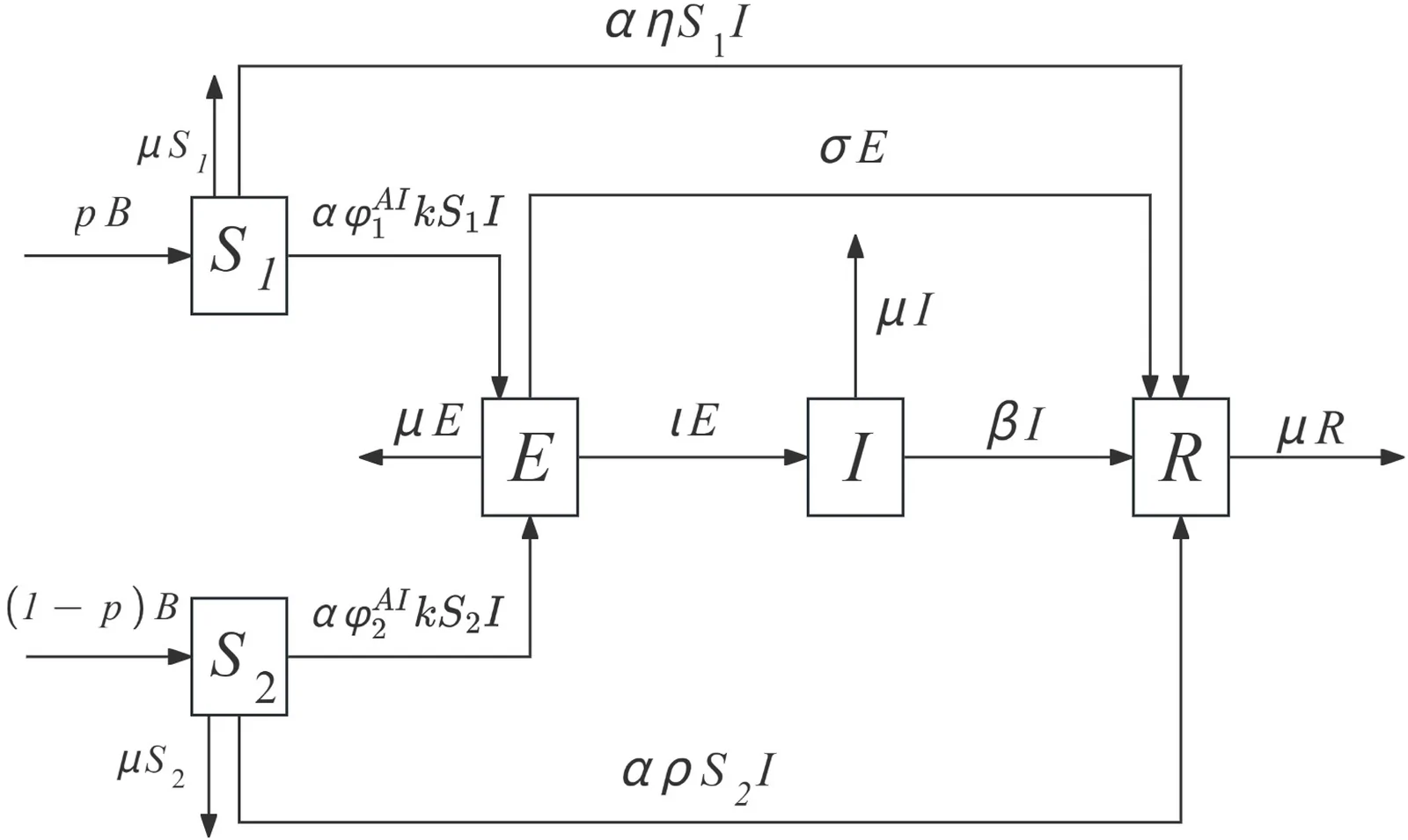
Flowchart of AI-enabled knowledge dissemination.

The product terms in the model simultaneously incorporate two types of mechanisms: α and *k* represent the intensity of knowledge contact generated through the employee–employee channel, whereas $\varphi_{1}^{AI}$ and $\varphi_{2}^{AI}$ represent the enhancing effects of the employee–AI channel on the efficiency of knowledge understanding and mastery. Together, these two mechanisms constitute the “interpersonal–human-machine” dual-channel coupling mechanism defined in this study.

Based on the above analysis, the 2*SEIR*-*CA* dynamic equations for knowledge dissemination proposed in this study are as follows:


$$\begin{array}{c} \;\{\begin{array}{c} \frac{dS_{1}}{dt}=pB-\alpha \eta S_{1}I-\alpha \varphi_{1}^{AI}kS_{1}I-\mu S_{1},\\ \frac{dS_{2}}{dt}=(1-p)B-\alpha \rho S_{2}I-\alpha \varphi_{2}^{AI}kS_{2}I-\mu S_{2},\\ \frac{dE}{dt}=\alpha \varphi_{1}^{AI}kS_{1}I+\alpha \varphi_{2}^{AI}kS_{2}I-\left(\sigma +\iota +\mu \right)E,\\ \frac{dI}{dt}=\iota E-\mu I-\beta I,\\ \frac{dR}{dt}=\alpha \eta S_{1}I+\alpha \rho S_{2}I+\sigma E+\beta I-\mu R. \end{array} \end{array}$$ (1)

Where $B>0,\mu >0,\alpha >0,\eta >0,\rho >0,\sigma >0,\iota >0,\beta >0,0<\varphi_{2}<\varphi_{1}<1,k>1$, and the system satisfies the following initial conditions:


$$\begin{array}{c} S_{1}\left(0\right)=S_{1,0}\geq 0,S_{2}\left(0\right)=S_{2,0}\geq 0,E\left(0\right)=E_{0}\geq 0,I\left(0\right)=I_{0},R\left(0\right)=R_{0}\geq 0 \end{array}$$

Since N(t) represents the total population, we have $N\left(t\right)=S_{1}\left(t\right)+S_{2}\left(t\right)+E\left(t\right)+I\left(t\right)+R\left(t\right)$. It follows that the total population satisfies $\frac{dN\left(t\right)}{dt}=B-\mu N\left(t\right)$, with solution $N\left(t\right)=\left(N_{0}-\frac{B}{\mu }\right)e^{-\mu t}+\frac{B}{\mu }$. Therefore, the system has a unique positive equilibrium given by $\underset{t\to \infty }{\lim}N\left(t\right)=\frac{B}{\mu }$. The positive invariant set of the system is:


$$\begin{array}{c} \;\Omega =\left\{\left(S_{1},S_{2},E,I,R\right)\in R^{5},N\left(t\right)\leq \frac{B}{\mu }\right\} \end{array}$$ (2)

This demonstrates that the model is bounded, mathematically well-posed, and organizationally interpretable.

### Spatiotemporal evolution rules based on cellular automata

To investigate the spatiotemporal coupling characteristics of knowledge dissemination in an AI-enabled setting, this study maps the aforementioned multi-stage 2*SEIR*-*CA* dynamic model onto a two-dimensional discrete grid to conduct a microscopic evolution analysis. The cellular automaton model is not independent of the continuous dynamic model; rather, it spatially maps the macroscopic dynamic mechanism while preserving the same five knowledge states and their core transition relationships. Whereas the continuous dynamic model describes changes in the numbers of employees in different states at the aggregate organizational level, the cellular automaton translates the macroscopic average-contact process into stochastic interactions within local neighborhoods, thereby enabling further examination of how local interactions among individuals give rise to organizational-level patterns of knowledge aggregation and spatiotemporal diffusion.

A two-dimensional grid of size L×L is defined, and periodic boundary conditions are employed to eliminate edge effects. Each cell in the spatiotemporal grid represents a node in the system (i.e., an employee), and the state of the cell at position (i,j) at discrete time *t* is denoted as $S_{ij}\left(t\right)$. The state space of the cells is the discrete set $\Omega =\left\{S_{1},S_{2},E,I,R\right\}$. In this study, the standard Moore neighborhood is adopted, meaning that each cell interacts with its eight surrounding adjacent cells (local degree $k_{CA}=8$). For the central cell (i,j), its neighborhood set is defined as:


$$\begin{array}{c} U_{ij}=\left\{\left(x,y\right)|\max\left(\left|x-i\right|,\left|y-j\right|\right)=1,\left(x,y\right)\neq \left(i,j\right)\right\} \end{array}$$

In the evolution process of the cellular automata, the transformation rates defined in the macroscopic continuous-time framework need to be converted into state-transition probabilities within a time step Δt. The transition mechanisms can be categorized into spontaneous evolution and contact-driven evolution.

#### Spontaneous evolution rules.

The spontaneous transformation process is not influenced by the states of neighboring nodes, and its state transition probability within a time step Δt is directly derived from the macroscopic continuous transformation rate.

For natural employee outflow, cells in all states leave the organizational system with probability $P_{\mu }=1-e^{-\mu \Delta t}$, becoming empty cells. In subsequent evolution, these empty cells are refilled with new individuals in state *S*_1_ or *S*_2_ with probabilities *p* and 1−p, respectively.

For the evolution of employees with strong knowledge mastery (*E*), the probability of transforming into knowledge-disseminating employees (*I*) is $P_{\left(E\to I\right)}=1-e^{-\iota \Delta t}$, and the probability of transforming into knowledge-exited employees (*R*) is $P_{\left(E\to R\right)}=1-e^{-\sigma \Delta t}$.

For the evolution of knowledge-disseminating employees (*I*), the probability of transforming into knowledge-exited employees (*R*) is $P_{\left(I\to R\right)}=1-e^{-\beta \Delta t}$.

#### Contact-driven evolution rules.

At time *t*, let $N_{I\left(i,j,t\right)}$ denote the number of knowledge-disseminating employees (*I*) within the Moore neighborhood of cell (i,j), where $0\leq N_{I}\leq 8$.

For employees who have not yet mastered the knowledge *S*_1_, the local theoretical transition probability of transforming into employees with strong knowledge mastery *E* upon contact is $P{\;}_{\left(S_{1}\to E\right)}^{\prime }=1-{\left(1-\alpha \varphi_{1}^{AI}\Delta t\right)}^{N_{I\left(i,j,t\right)}}$, and the local theoretical transition probability of directly transforming into knowledge-exited employees *R* is $P{\;}_{\left(S_{1}\to R\right)}^{\prime }=1-{\left(1-\alpha \eta \Delta t\right)}^{N_{I\left(i,j,t\right)}}$.

For employees with limited knowledge mastery *S*_2_, the local theoretical transition probability of transforming into employees with strong knowledge mastery *E* upon contact is $P{\;}_{\left(S_{2}\to E\right)}^{\prime }=1-{\left(1-\alpha \varphi_{2}^{AI}\Delta t\right)}^{N_{I\left(i,j,t\right)}}$, and the local theoretical transition probability of directly transforming into knowledge-exited employees *R* is $P{\;}_{\left(S_{2}\to R\right)}^{\prime }=1-{\left(1-\alpha \rho \Delta t\right)}^{N_{I\left(i,j,t\right)}}$.

A synchronous update mechanism is adopted, in which all cells update their states simultaneously at each discrete time step t→t+1. For *S*_1_ and *S*_2_, there are two mutually exclusive pathways within the same time step—transforming into *E* or *R*—and proportional normalization is applied.

Taking *S*_1_ as an example, the actual transition probabilities are:


$$\begin{array}{c} P_{\left(S_{1}\to E\right)}=\left[P^{\prime }\left(S_{1}\to E\right)/\left(P^{\prime }\left(S_{1}\to E\right)+P^{\prime }\left(S_{1}\to R\right)\right)\right]\times \left[1-\left(1-P^{\prime }\left(S_{1}\to E\right)\left(1-P_{\left(S_{1}\to R\right)}^{\prime }\right)\right)\right] \end{array}$$

$$\begin{array}{c} P_{\left(S_{1}\to R\right)}=\left[P^{\prime }\left(S_{1}\to R\right)/\left(P^{\prime }\left(S_{1}\to E\right)+P^{\prime }\left(S_{1}\to R\right)\right)\right]\times \left[1-\left(1-P^{\prime }\left(S_{1}\to E\right)\right)\left(1-P_{\left(S_{1}\to R\right)}^{\prime }\right)\right] \end{array}$$

Based on the actual transition probabilities described above, the system generates an independent uniformly distributed random number ξ∈(0,1) for each cell at each time step to determine its state transition.

### Parameterization and simulation settings

To improve the transparency, reproducibility, and comparability of numerical simulations, a unified set of baseline specifications is adopted for the continuous dynamic model, optimal control model, and cellular automaton model. Except for the subcritical threshold comparison scenario, the continuous model employs the baseline parameter values listed in Table 2, with the initial state specified as


$$\begin{array}{c} \left(S_{1}\left(0\right),S_{2}\left(0\right),E\left(0\right),I\left(0\right),R\left(0\right)\right)=\left(10,5,2,1,0\right), \end{array}$$

corresponding to an initial organizational population of 18 employees. This specification indicates that, at the onset of knowledge dissemination, the organization comprises 10 employees who have not yet mastered the relevant knowledge, 5 employees with limited knowledge mastery, 2 employees with strong knowledge mastery who have not yet participated in dissemination, and 1 initial knowledge-disseminating employee, while no employees have entered the knowledge-exited state. To facilitate comparison among different simulation scenarios, the baseline initial population composition is consistently adopted in parameter analyses and control analyses unless otherwise specified. For robustness evaluation, additional initial conditions are introduced to examine whether the main evolutionary patterns depend on specific initial population structures.

The continuous dynamical equations are solved using the *ode*45 solver in MATLAB. For the threshold dynamics simulations, the time interval is set to t∈[0,100], with an output interval of 0.1. For the steady-state parameter analysis, the time interval is set to t∈[0,1000], and the relative and absolute error tolerances are set to $10^{-6}$ and $10^{-8}$, respectively, to ensure that the system sufficiently approaches a stable state. The optimal control system is solved using the forward–backward sweep method, with both the state equations and the adjoint equations numerically integrated using the fourth-order Runge–Kutta method. The optimal control simulation is conducted over t∈[0,150] with 1,500 time nodes. The iteration is terminated when the maximum absolute difference between the four control trajectories obtained from two successive iterations is less than $10^{-3}$, and the maximum number of iterations is set to 600. Here, *t* denotes dimensionless simulation time and does not directly correspond to a specific number of days, weeks, or months. To further clarify the numerical implementation procedure of the optimal control model, Fig. 2 presents the computational workflow of the optimal control simulation, including scenario initializa*t*ion, forward–backward iteration, convergence evaluation, and result output.

**Fig 2:**
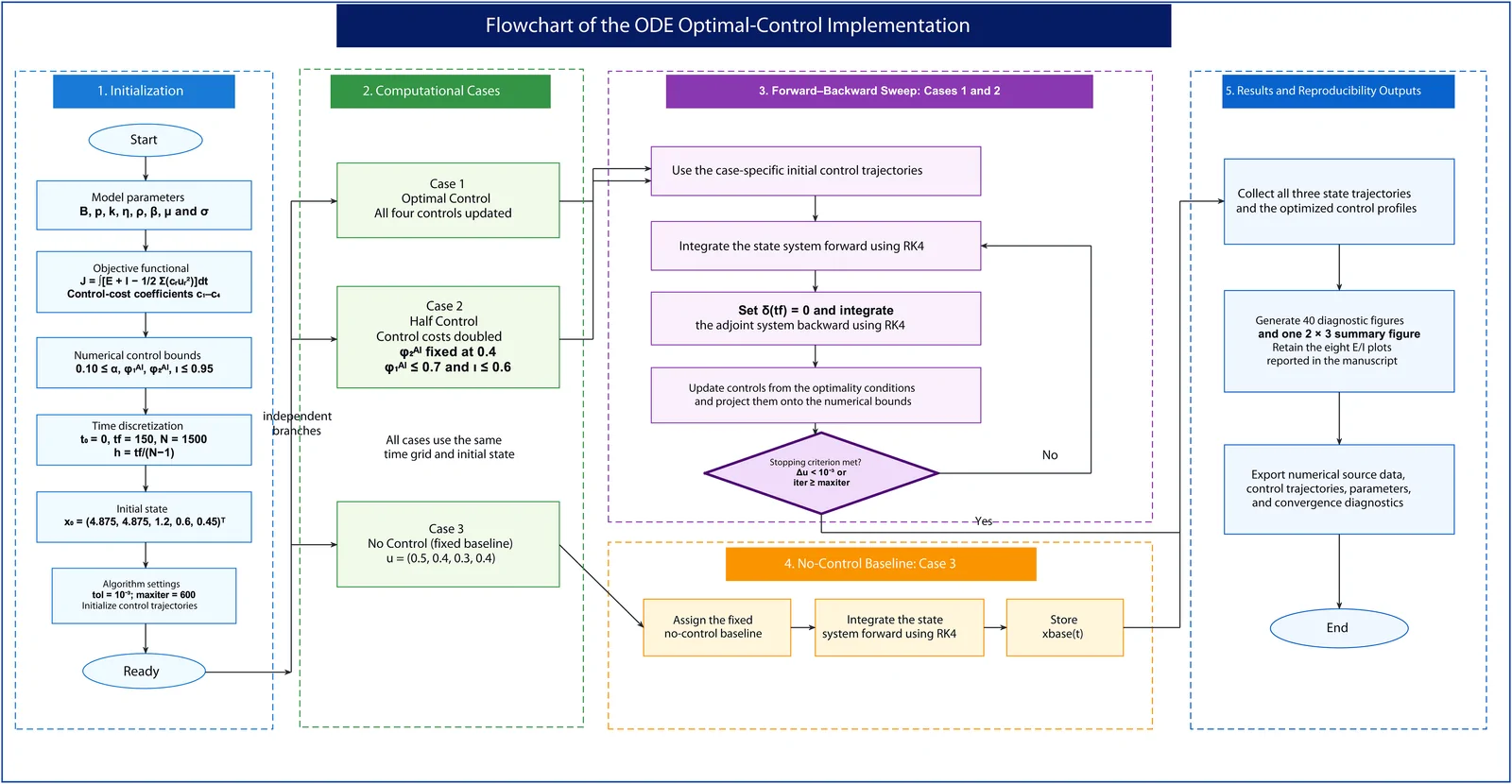
Numerical implementation procedure of optimal control for AI-enabled knowledge dissemination.

In the cellular automata simulations, each cell represents a representative employee in a specific knowledge state. The baseline spatial domain is defined as a 60×60 two-dimensional square grid comprising 3,600 cells. Consistent with the baseline initial composition of the continuous model, the initial numbers of cells in the five states are set to 2,000, 1,000, 400, 200, and 0, corresponding to the $S_{1},S_{2},E,I$ and *R* states, respectively. Under the baseline scenario, while holding the total number of cells in each state constant, the initial *E* and *I* cells are spatially clustered around the center of the grid to simulate a process in which knowledge first emerges within a local team or core knowledge group in the organization and subsequently diffuses outward. Cell states are updated synchronously according to the local states within the Moore eight-neighbor neighborhood, and periodic boundary conditions are adopted to mitigate the influence of finite boundaries on the dissemination process. The baseline grid is simulated for 150 iteration steps, with the time-scaling parameter set to *dt* = 0.1. Representative spatial snapshots are generated using a fixed random seed of 42. For the random-seed robustness analysis, 30 prespecified independent random seeds are employed, and the mean, variability, and 95% simulation interval are reported based on repeated simulation runs. The details are shown in Table 3. To further clarify the numerical implementation procedure of the spatial simulation, Fig. 3 presents the overall workflow of the cellular automata (CA) simulation, including grid initialization, control scenario specification, synchronous state updating, stochastic state transition evaluation, and spatiotemporal result output.

**Table 3:** Baseline numerical settings of the continuous and cellular automaton models.

Simulation module	Baseline initial scale	Temporal setting	Numerical method	Other settings
Threshold dynamics	N(0)=18,(10,5,2,1,0)	0−100, output interval = 0.1	MATLAB *ode*45 solver	The same baseline initial condition was adopted for the numerical comparison of the *R*_0_ < 1 and *R*_0_ > 1 scenarios, while the parameter sets were adjusted to generate different threshold regimes.
Steady-state parameter analysis	N(0)=18,(10,5,2,1,0)	0−1000	MATLAB *ode*45 solver	RelTol = 10^–6^; AbsTol = 10^–8^.
Optimal-control analysis	N(0)=18,(10,5,2,1,0)	0−150,1,500 time nodes	Forward–backward sweep method with fourth-order Runge–Kutta integration	Convergence tolerance = 10^–3^; maximum of 600 iterations.
Baseline CA simulation	60×60=3600 cells	150steps,*dt* = 0.1	Synchronous updating	Moore neighborhood; periodic boundary conditions.
Initial CA composition	2000,1000,400,200,0	—	Fixed-count initialization	The baseline spatiotemporal snapshots adopt a centrally clustered hotspot distribution, whereas the robustness analysis employs a randomly dispersed distribution across the entire grid.
Stochastic CA implementation	Baseline snapshot seed = 42	—	Stochastic state transitions	The robustness analysis employs 30 prespecified random seeds, and the simulation run with a terminal coverage rate closest to the median is selected as the representative result.
CA grid robustness	40×40,60×60,80×80	Each run comprises 150 iteration steps	Identical CA update rules	Each grid maintains identical initial state proportions, neighborhood rules, boundary conditions, and model parameters.

**Fig 3:**
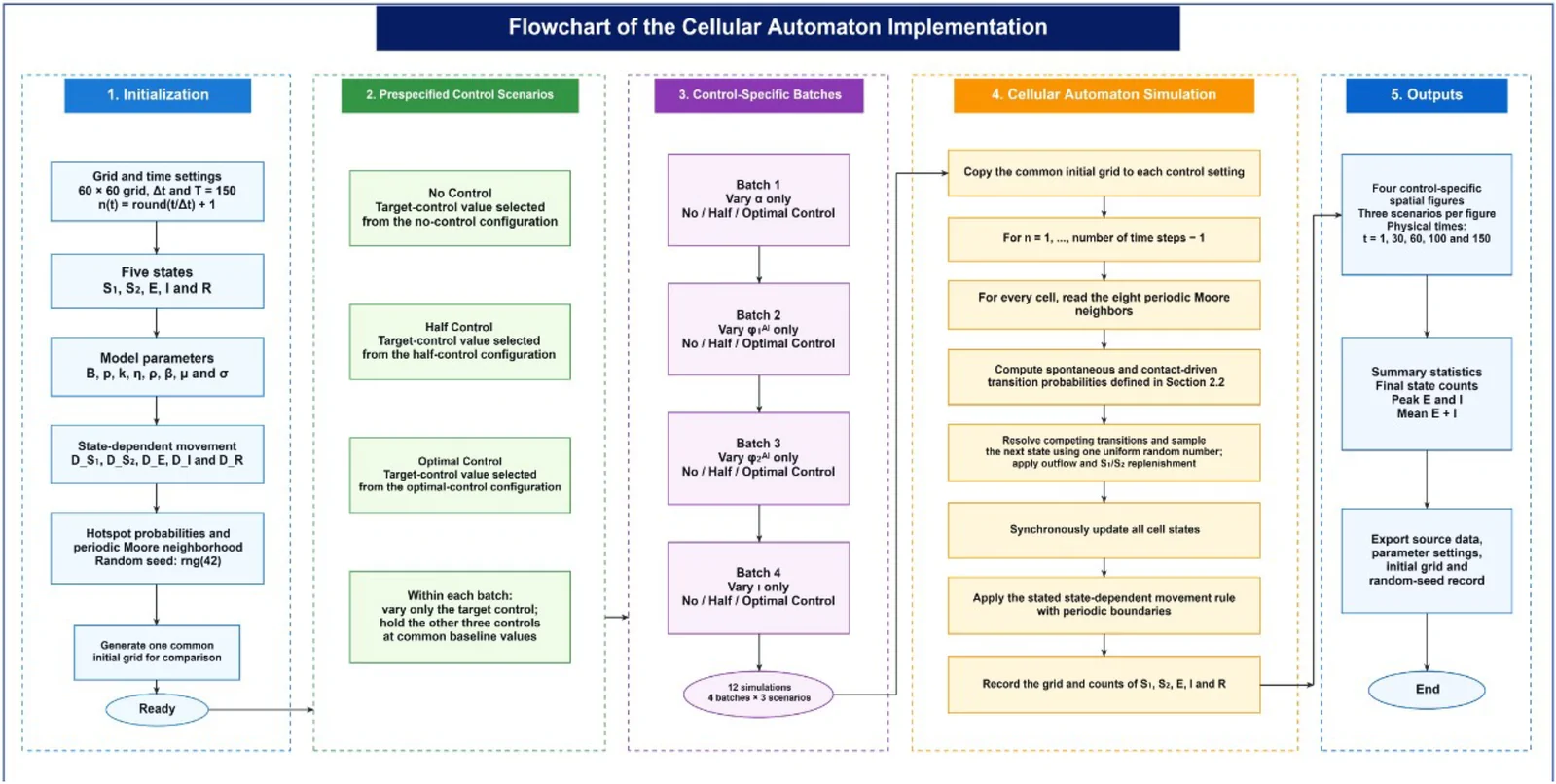
Cellular automaton-based procedure for simulating the spatiotemporal evolution of knowledge dissemination.

To further examine whether the numerical results depend on specific parameter values, the initial population composition, or spatial simulation settings, this study conducts robustness analyses at both the continuous dynamical model and cellular automata model levels. For the continuous dynamical model, while holding all other parameters at their baseline values, each of the 12 parameters listed in Table 2 is varied independently over 21 equally spaced values within its respective exploration range, yielding a total of 252 single-parameter perturbation simulations. This design enables the stability of the system’s dynamic characteristics to be examined as each parameter varies across its full feasible range, while avoiding complex scenarios that are difficult to interpret because of the simultaneous combination of multiple extreme parameter values. For the initial-condition robustness analysis, the initial total population is held constant at *N*(0) = 18, and four scenarios are considered: the baseline initial state, no initial knowledge-disseminating employees, a higher initial number of knowledge-disseminating employees, and a stronger initial knowledge foundation.

For the cellular automata model, two-dimensional grids of 40×40, 60×60, and 80×80 are employed to examine finite-size effects. Across all grid sizes, the initial proportions of the five cell states, model parameters, Moore neighborhood, periodic boundary conditions, and number of iteration steps are held constant. To prevent changes in the perimeter-to-area relationship of a single central hotspot across grid sizes from confounding grid comparisons, the grid-size and random-seed robustness analyses employ fixed-count initialization with cells randomly dispersed across the entire grid, whereas the original spatiotemporal diffusion snapshots retain the centrally clustered hotspot initialization. For each grid size, 30 prespecified random seeds are used for independent simulation runs, and the distributional characteristics of the terminal knowledge coverage rate are reported. For the baseline 60×60 grid, the mean and pointwise 95% simulation interval are further calculated across the 30 runs, and the trajectory whose terminal knowledge coverage rate is closest to the median of the 30 runs is selected as the representative stochastic implementation.


$$\begin{array}{c} C_{K}\left(t\right)=\frac{S_{2}\left(t\right)+E\left(t\right)+I\left(t\right)}{L^{2}}, \end{array}$$

Here, $C_{K}(t)$ denotes the knowledge coverage rate of the cellular automata model at the *t*th iteration s*t*ep, and *L* denotes the side length of the square grid. Because the *S*_2_, *E*, and *I* states represent employees with limited knowledge mastery, employees with strong knowledge mastery, and knowledge-disseminating employees, respectively, the sum of these three states is used to measure the proportion of employees within the organization who have acquired the relevant knowledge to varying degrees. Results across different grid sizes are compared using the normalized knowledge coverage rate rather than the absolute numbers of cells.

## Stability analysis of the model

First, it is necessary to prove the existence of the equilibrium point $\Lambda =\left(S_{1},S_{2},E,I,R\right)$ of System 1. It is straightforward to obtain the knowledge-free equilibrium of System 1 as $\Lambda_{0}=\left(\frac{pB}{\mu },\frac{\left(1-p\right)B}{\mu },0,0,0\right)$, which implies that the number of knowledge-disseminating employees in System 1 tends to zero.

Then, the basic reproduction number *R*_0_ of System 1 can be calculated using the next-generation matrix. The basic reproduction number is critical for designing system interventions, as it represents the expected secondary transmissions generated by a single knowledge-disseminating employee.

Let $X={\left(E,I,R,S_{1},S_{2}\right)}^{T}$. Accordingly, System 1 can be written as:


$$\begin{array}{c} \;\frac{dX}{dt}=F\left(X\right)-V\left(X\right). \end{array}$$ (3)

$$\begin{array}{c} F\left(X\right)=\left(\begin{array}{c} \alpha \varphi_{1}^{AI}kS_{1}I+\alpha \varphi_{2}^{AI}kS_{2}I\\ \iota E\\ 0\\ 0\\ 0 \end{array}\right),\begin{array}{c} V\left(X\right)=\left(\begin{array}{c} \left(\sigma +\iota +\mu \right)E\\ \left(\beta +\mu \right)I\\ -pB+\alpha \eta S_{1}I+\alpha \varphi_{1}^{AI}kS_{1}I+\mu S_{1}\\ -\left(1-p\right)B+\alpha \rho S_{2}I+\alpha \varphi_{2}^{AI}kS_{2}I+\mu S_{2}\\ \mu R-\alpha \eta S_{1}I-\alpha \rho S_{2}I-\sigma E-\beta I \end{array}\right) \end{array} \end{array}$$ (4)

It follows that:


$$\begin{array}{c} F=\left(\begin{array}{cc} 0 & \alpha k\left(\varphi_{1}^{AI}S_{1}+\varphi_{2}^{AI}S_{2}\right)\\ \iota & 0 \end{array}\right),V=\left(\begin{array}{cc} \sigma +\iota +\mu & 0\\ 0 & \beta +\mu \end{array}\right) \end{array}$$ (5)

where *F* and *V* denote the infection matrix and the transition matrix, respectively. By simple calculation, the inverse matrix of *V* can be obtained as:


$$\begin{array}{c} \;V^{-1}=\left(\begin{array}{cc} \frac{1}{\sigma +\iota +\mu } & 0\\ 0 & \frac{1}{\beta +\mu } \end{array}\right) \end{array}$$ (6)

The next generation matrix is:


$$\begin{array}{c} \;FV^{-1}=\left(\begin{array}{cc} 0 & \frac{\alpha k\left(\varphi_{1}^{AI}S_{1}+\varphi_{2}^{AI}S_{2}\right)}{\beta +\mu }\\ \frac{\iota }{\sigma +\iota +\mu } & 0 \end{array}\right), \end{array}$$ (7)

Therefore, the basic reproduction number *R*_0_ of System 1 is given by the spectral radius of the next-generation matrix $FV^{-1}$, where the spectral radius is the largest eigenvalue of *FV*^–1^. Hence, *R*_0_ can be calculated as:


$$\begin{array}{c} \;R_{0}=\rho \left(FV^{-1}\right)=\sqrt{\frac{\alpha k\iota \left(\varphi_{1}^{AI}S_{1}+\varphi_{2}^{AI}S_{2}\right)}{\left(\sigma +\iota +\mu \right)\left(\beta +\mu \right)}}. \end{array}$$ (8)

If *R*_0_ > 1, the knowledge-present equilibrium of System 1 can be expressed as $\Lambda^{*}=\left(S_{1}^{*},S_{2}^{*},E^{*},I^{*},R^{*}\right)$, indicating that knowledge will be widely disseminated. The knowledge-present equilibrium $\Lambda^{*}$ satisfies:


$$\begin{array}{c} \;\{\begin{array}{c} pB-\alpha \eta S_{1}^{*}I^{*}-\alpha \varphi_{1}^{AI}kS_{1}^{*}I^{*}-\mu S_{1}^{*}=0,\\ \left(1-p\right)B-\alpha \rho S_{2}^{*}I^{*}-\alpha \varphi_{2}^{AI}kS_{2}^{*}I^{*}-\mu S_{2}^{*}=0,\\ \alpha \varphi_{1}^{AI}kS_{1}^{*}I^{*}+\alpha \varphi_{2}^{AI}kS_{2}^{*}I^{*}-\left(\sigma +\iota +\mu \right)E^{*}=0,\\ \iota E^{*}-\mu I^{*}-\beta I^{*}=0,\\ \alpha \eta S_{1}^{*}I^{*}+\alpha \rho S_{2}^{*}I^{*}+\sigma E^{*}+\beta I^{*}-\mu R^{*}=0, \end{array} \end{array}$$ (9)

by solving the system of equations in Eq. 9, we obtain:


$$\begin{array}{c} S_{1}^{*}=\frac{pB}{\alpha I^{*}\left(\eta +\varphi_{1}^{AI}k\right)+\mu },S_{2}^{*}=\frac{\left(1-p\right)B}{\alpha I^{*}\left(\rho +\varphi_{2}^{AI}k\right)+\mu },E^{*}=\frac{\left(\beta +\mu \right)I^{*}}{\iota }. \end{array}$$ (10)

Substituting Eq. 10 into Eq. 9 and solving the resulting system yields:


$$\begin{array}{c} I^{*}=\frac{-b+\sqrt{\begin{array}{c} b^{2}-4\left(\alpha \eta +\alpha \varphi_{1}^{AI}k\right)\left(\alpha \rho +\alpha \varphi_{2}^{AI}k\right){\left[\left(\sigma +\iota +\mu \right)\left(\beta +\mu \right)\right]}^{2}\mu^{2}\\ +4\left(\alpha \eta +\alpha \varphi_{1}^{AI}k\right)\left(\alpha \rho +\alpha \varphi_{2}^{AI}k\right)\left(\sigma +\iota +\mu \right)\left(\beta +\mu \right)\\ \left[pB\alpha \varphi_{1}^{AI}k\iota +\left(1-p\right)B\alpha \varphi_{2}^{AI}k\iota \right]\mu \end{array}}}{2\left(\alpha \eta +\alpha \varphi_{1}^{AI}k\right)\left(\alpha \rho +\alpha \varphi_{2}^{AI}k\right)\left(\sigma +\iota +\mu \right)\left(\beta +\mu \right)}, \end{array}$$ (11)

where:


$$\begin{array}{cc} b & =\left(\alpha \eta +\alpha \varphi_{1}^{AI}k\right)\cdot \left(\sigma +\iota +\mu \right)\left(\beta +\mu \right)\cdot \mu +\left(\alpha \rho +\alpha \varphi_{2}^{AI}k\right)\cdot \left(\sigma +\iota +\mu \right)\left(\beta +\mu \right)\cdot \mu \\ & \;-pB\alpha \varphi_{1}^{AI}k\iota \cdot \left(\alpha \rho +\alpha \varphi_{2}^{AI}k\right)-\left(1-p\right)B\alpha \varphi_{2}^{AI}k\iota \cdot \left(\alpha \eta +\alpha \varphi_{1}^{AI}k\right). \end{array}$$

Substituting Eq. 10 into Eq. 9 and simplifying yields:


$$\begin{array}{c} R^{*}=\frac{1}{\mu }\left[\frac{\alpha \eta pBI^{*}}{\alpha I^{*}\left(\eta +\varphi_{1}^{AI}k\right)+\mu }+\frac{\left(1-p\right)B\alpha \rho I^{*}}{\alpha I^{*}\left(\rho +\varphi_{2}^{AI}k\right)+\mu }+\beta I^{*}+\frac{\sigma \left(\beta +\mu \right)I^{*}}{\iota }\right]. \end{array}$$ (12)

**Theorem 1.** *If R*_*0*_ *< 1 and* $\sigma \mu^{2}+\sigma \beta \mu +\iota \mu^{2}+\iota \beta \mu +\mu^{3}+\beta \mu^{2}>B\alpha \varphi_{2}^{AI}k\iota$*, the knowledge-free equilibrium* $\Lambda_{0}=\left(\frac{pB}{\mu },\frac{\left(1-p\right)B}{\mu },0,0,0\right)$ *of System 1 is locally asymptotically stable.*

**Proof 1***. The Jacobian matrix of System 1 at the knowledge-free equilibrium* $\Lambda_{0}=\left(\frac{pB}{\mu },\frac{\left(1-p\right)B}{\mu },0,0,0\right)$ *can be expressed as:*


$$\begin{array}{c} \;J\left(\Lambda_{0}\right)=\left[\begin{array}{ccccc} -\mu & 0 & 0 & -\frac{pB\alpha \eta }{\mu }-\frac{pB\alpha \varphi_{1}^{AI}k}{\mu } & 0\\ 0 & -\mu & 0 & -\frac{\left(1-p\right)B\alpha \rho }{\mu }-\frac{\left(1-p\right)B\alpha \varphi_{2}^{AI}k}{\mu } & 0\\ 0 & 0 & -\sigma -\iota -\mu & \frac{pB\alpha \varphi_{1}^{AI}k+\left(1-p\right)B\alpha \varphi_{2}^{AI}k}{\mu } & 0\\ 0 & 0 & \iota & -\mu -\beta & 0\\ 0 & 0 & \sigma & \frac{pB\alpha \eta }{\mu }+\frac{\left(1-p\right)B\alpha \rho }{\mu } & -\mu \end{array}\right] \end{array}$$ (13)

*The negative eigenvalues of* J(Λ0) *can be readily obtained as* $h_{01}=h_{02}=h_{03}=-\mu <0$*, while the remaining eigenvalues are the characteristic roots of* $\left|h\Lambda -J\left(\Lambda_{0}\right)\right|$*, where:*


$$\begin{array}{c} \left|h\Lambda -J\left(\Lambda_{0}\right)\right|=\left|\begin{array}{cc} h+\sigma +\iota +\mu & -\frac{pB\alpha \varphi_{1}^{AI}k+\left(1-p\right)B\alpha \varphi_{2}^{AI}k}{\mu }\\ -\iota & h+\mu +\beta \end{array}\right| \end{array}$$ (14)

Eq. 14 *yields the following quadratic polynomial:*


$$\begin{array}{c} \;\left|h\Lambda -J\left(\Lambda_{0}\right)\right|=Q_{3}h^{2}+Q_{2}h+Q_{1}, \end{array}$$ (15)

*where:*


$$\begin{array}{c} \begin{array}{c} Q_{3}=1,\\ Q_{2}=2\mu +\beta +\sigma +\iota ,\\ Q_{1}=\sigma \mu +\sigma \beta +\iota \mu +\iota \beta +\mu^{2}+\beta \mu -\frac{\left[pB\varphi_{1}^{AI}+\left(1-p\right)B\varphi_{2}^{AI}\right]\alpha k\iota }{\mu }. \end{array} \end{array}$$ (16)

*From* Eq. 16*, it follows that the existence of the equilibrium point requires:*


$$\begin{array}{c} \;\sigma \mu +\sigma \beta +\iota \mu +\iota \beta +\mu^{2}+\beta \mu >\frac{\left[pB\varphi_{1}^{AI}+\left(1-p\right)B\varphi_{2}^{AI}\right]\alpha k\iota }{\mu }, \end{array}$$ (17)

*From* Eq. 17*, we obtain:*


$$\begin{array}{c} \;\sigma \mu^{2}+\sigma \beta \mu +\iota \mu^{2}+\iota \beta \mu +\mu^{3}+\beta \mu^{2}>pB\varphi_{1}^{AI}\alpha k\iota +B\varphi_{2}^{AI}\alpha k\iota -pB\varphi_{2}^{AI}\alpha k\iota , \end{array}$$ (18)

*where the term* $pB\varphi_{1}^{AI}\alpha k\iota -pB\varphi_{2}^{AI}\alpha k\iota$ *is assumed to be negligible, that is:*


$$\begin{array}{c} \;\sigma \mu^{2}+\sigma \beta \mu +\iota \mu^{2}+\iota \beta \mu +\mu^{3}+\beta \mu^{2}>B\alpha \varphi_{2}^{AI}k\iota . \end{array}$$ (19)

*When the condition* $\sigma \mu^{2}+\sigma \beta \mu +\iota \mu^{2}+\iota \beta \mu +\mu^{3}+\beta \mu^{2}>B\alpha \varphi_{2}^{AI}k\iota$ *is satisfied, namely when R*_*0*_ *< 1, the eigenvalues h*_*04*_ *and h*_*05*_ *are negative.*

*In summary, when R*_*0*_ *< 1, the knowledge-free equilibrium* $\Lambda_{0}=\left(\frac{pB}{\mu },\frac{\left(1-p\right)B}{\mu },0,0,0\right)$ *is locally asymptotically stable.*

**Theorem 2.** *If R*_*0*_ *< 1 and* $\alpha \left[pB\varphi_{1}^{AI}k+\left(1-p\right)B\varphi_{2}^{AI}k+pB\eta +\left(1-p\right)B\rho \right]\leq \mu^{2}$*, then the knowledge-free equilibrium* $\Lambda_{0}=\left(\frac{pB}{\mu },\frac{\left(1-p\right)B}{\mu },0,0,0\right)$ *is globally asymptotically stable.*

**Proof 2***. It follows that* $S_{1}\left(t\right)+S_{2}\left(t\right)+E\left(t\right)+I\left(t\right)+R\left(t\right)=N\left(t\right)$ *and that* N(t) *satisfies* $\frac{dN\left(t\right)}{dt}\leq B-\mu S_{1}\left(t\right)-\mu S_{2}\left(t\right).$


$$\begin{array}{c} \;\underset{t\to 0}{\lim}\sup N\left(t\right)\leq \frac{B}{\mu }. \end{array}$$ (20)

*For* t≥0*, the positively invariant set of System 1 can be expressed as:*


$$\begin{array}{c} \;T=\left\{\left(S_{1}\left(t\right),S_{2}\left(t\right),E\left(t\right),I\left(t\right),R\left(t\right)\in R_{5}^{+}\right):S_{1}\left(t\right)+S_{2}\left(t\right)+E(t)+I\left(t\right)+R(t)\leq \frac{B}{\mu }\right\}, \end{array}$$ (21)

*Then, a Lyapunov function can be constructed as* L(t)=E(t)+I(t)+R(t)*, and where* $S_{1}=\frac{pB}{\mu },S_{2}=\frac{\left(1-p\right)B}{\mu },$*then its derivative* $L^{\prime }\left(t\right)$ *is calculated as:*


$$\begin{array}{cc} L^{\prime }\left(t\right) & =\alpha \varphi_{1}^{AI}kS_{1}I+\alpha \varphi_{2}^{AI}kS_{2}I-\left(\sigma +\iota +\mu \right)E+\iota E-\mu I-\beta I+\alpha \eta S_{1}I\\ & \;+\alpha \rho S_{2}I+\sigma E+\beta I-\mu R,\\ & =\left(\alpha \varphi_{1}^{AI}kS_{1}+\alpha \varphi_{2}^{AI}kS_{2}+\alpha \eta S_{1}+\alpha \rho S_{2}-\mu \right)I-\mu \left(E+R\right),\\ & \leq \left(\alpha \varphi_{1}^{AI}k\frac{pB}{\mu }+\alpha \varphi_{2}^{AI}k\frac{\left(1-p\right)B}{\mu }+\alpha \eta \frac{PB}{\mu }+\alpha \rho \frac{\left(1-p\right)B}{\mu }-\mu \right)I-\mu \left(E+R\right). \end{array}$$ (22)

*By simplifying* Eq. 22*, it can be obtained that:*


$$\begin{array}{c} \alpha \left[pB\varphi_{1}^{AI}k+\left(1-p\right)B\varphi_{2}^{AI}k+pB\eta +\left(1-p\right)B\rho \right]\leq \mu^{2}, \end{array}$$ (23)

*It is easy to verify that* $L^{\prime }\left(t\right)\leq 0,$ *if* $S_{1}+S_{2}\leq \frac{B}{\mu }$*,and* $\alpha \left[pB\varphi_{1}^{AI}k+\left(1-p\right)B\varphi_{2}^{AI}k+pB\eta +\left(1-p\right)B\rho \right]\leq \mu^{2}$.

*Moreover, the necessary and sufficient condition for* $L^{\prime }\left(t\right)=0$ *to hold is* $L^{\prime }\left(t\right)=0,E=I=R$*. From System 1, it follows that when* $L^{\prime }\left(t\right)=0$, $\Lambda_{0}$ *is the unique solution in T. Based on the Lyapunov–LaSalle invariance principle, every solution of System 1 is shown to approach* $\Lambda_{0}$ *as* t→∞*. Therefore, the knowledge-free equilibrium* $\Lambda_{0}=\left(\frac{pB}{\mu },\frac{\left(1-p\right)B}{\mu },0,0,0\right)$ *of System 1 is globally asymptotically stable.*

**Theorem 3.** *If R*_*0*_ *> 1 and the system parameters satisfy the condition H*_*1*_*, the knowledge-present equilibrium point* $\Lambda^{*}$ *of the knowledge dissemination system is locally asymptotically stable.*

**Proof 3***. To examine the local asymptotic stability of the knowledge-present equilibrium point* $\Lambda^{*}$, System 1 is linearized at $\Lambda^{*}$. The Jacobian matrix of the system at the knowledge-present equilibrium point $\Lambda^{*}=\left(S_{1}^{*},S_{2}^{*},E^{*},I^{*},R^{*}\right)$ *is:*


$$\begin{array}{c} J\left(\Lambda^{*}\right)=\left[\begin{array}{ccccc} -\alpha \eta I^{*}-\alpha \varphi_{1}^{AI}kI^{*}-\mu & 0 & 0 & -\alpha \eta S_{1}^{*}-\alpha \varphi_{1}^{AI}kS_{1}^{*} & 0\\ 0 & -\alpha \rho I^{*}-\alpha \varphi_{2}^{AI}kI^{*}-\mu & 0 & -\alpha \rho S_{2}^{*}-\alpha \varphi_{2}^{AI}kS_{2}^{*} & 0\\ \alpha \varphi_{1}^{AI}kI^{*} & \alpha \varphi_{2}^{AI}kI^{*} & -\left(\sigma +\iota +\mu \right) & \alpha \varphi_{1}^{AI}kS_{1}^{*}+\alpha \varphi_{2}^{AI}kS_{2}^{*} & 0\\ 0 & 0 & \iota & -\mu -\beta & 0\\ \alpha \eta I^{*} & \alpha \rho I^{*} & \sigma & \alpha \eta S_{1}^{*}+\alpha \rho S_{2}^{*}+\beta & -\mu \end{array}\right] \end{array}$$ (24)

*The negative eigenvalue of* $J\left(\Lambda^{*}\right)$ *is obtained as* $h_{11}=-\mu <0,$ *while the remaining eigenvalues are the characteristic roots of* $\left|h\Lambda -J\left(\Lambda^{*}\right)\right|$*, where:*


$$\begin{array}{c} \left|h\Lambda -J\left(\Lambda^{*}\right)\right|=\left|\begin{array}{cccc} h+\alpha \eta I^{*}+AI^{*}+\mu & 0 & 0 & \alpha \eta S_{1}^{*}+AS_{1}^{*}\\ 0 & h+\alpha \rho I^{*}+CI^{*}+\mu & 0 & \alpha \rho S_{2}^{*}+CS_{2}^{*}\\ -AI^{*} & -CI^{*} & h+D & -AS_{1}^{*}-CS_{2}^{*}\\ 0 & 0 & -\iota & h+G \end{array}\right|, \end{array}$$ (25)

*where* $A=\alpha \varphi_{1}^{AI}k,C=\alpha \varphi_{2}^{AI}k,D=\sigma +\iota +\mu ,G=\mu +\beta .$

*From* $\left|h\Lambda -J\left(\Lambda^{*}\right)\right|$*, we obtain:*


$$\begin{array}{cc} \left|h\Lambda -J\left(\Lambda^{*}\right)\right| & =h^{4}+\left\{\left[\left(\alpha \rho +C+\alpha \eta +A\right)I^{*}+G+D+2\mu \right]\right\}h^{3}+\{[(\alpha \rho G+\alpha \rho D\\ & \;+CG+CD+\alpha \eta G+\alpha \eta D+\alpha \eta \mu +AG+AD+\mu A+\alpha \rho \mu +\mu C)I^{*}\\ & \;+\left(\alpha^{2}\eta \rho +\alpha \eta C+\alpha \rho A+AC\right)I^{{*}^{2}}+DG+2\mu G+2\mu D+\mu^{2}-\iota AS_{1}^{*}\\ & \;-\iota CS_{2}^{*}]\}h^{2}+\{[(\alpha \rho DG+CDG+\alpha \rho \iota CS_{2}^{*}+\alpha \eta DG+\alpha \eta \mu G+\alpha \eta \mu D\\ & \;+ADG+\mu AG+\mu AD+\alpha \rho \mu +\alpha \rho \mu G+\alpha \rho \mu D+\mu CG+\mu CD)I^{*}\\ & \;+(\alpha^{2}\eta \rho G+\alpha^{2}\eta \rho D+\alpha \eta CG+\alpha \eta CD+\alpha \rho AG+\alpha \rho AD+ACG\\ & \;+ACD)I^{{*}^{2}}+2\mu DG+\left(D+G\right)\mu^{2}-(2\iota C^{2}S_{2}^{*}+\alpha \rho \iota AS_{1}^{*}+\alpha \rho \iota CS_{2}^{*}\\ & \;+\iota ACS_{1}^{*}+\alpha \eta \iota CS_{2}^{*}+\iota ACS_{2}^{*})I^{*}-2\iota \mu A\iota ACS_{1}^{*}-2\iota \mu C\iota ACS_{2}^{*}]\}h\\ & \;+\{[(\alpha^{2}\rho \eta DG+\alpha \eta CDG+\alpha \rho ADG+ACDG-2\alpha \eta \iota C^{2}S_{2}^{*}-2\iota AC^{2}S_{2}^{*})I^{{*}^{2}}\\ & \;+(\alpha \eta \mu DG+\mu ADG+\alpha \rho \mu DG-\alpha \eta \iota \mu CS_{2}^{*}-\iota \mu ACS_{2}^{*}-\iota \mu C^{2}S_{2}^{*}\\ & \;-\alpha \rho \iota \mu AS_{1}^{*}-\iota \mu ACS_{1}^{*}-\iota \mu C^{2}S_{2}^{*})I^{*}+\mu^{2}DG-\iota \mu^{2}AS_{2}^{*}-\iota \mu^{2}CS_{2}^{*}]\}, \end{array}$$ (26)

*According to* Eq. 26*, a quartic polynomial can be constructed, in which* $Q_{5},Q_{4},Q_{3},Q_{2},Q_{1}$ *are used to denote the coefficients so as to determine the remaining eigenvalues of the system. Therefore, the above expression can be rewritten as:*


$$\begin{array}{c} \;Q_{5}h^{4}+Q_{4}h^{3}+Q_{3}h^{2}+Q_{2}h+Q_{1}=0, \end{array}$$ (27)

*where the coefficients* $Q_{5},Q_{4},Q_{3},Q_{2},Q_{1}$ *are algebraic expressions composed of the elements of the Jacobian matrix* $J\left(\Lambda^{*}\right)$*, and their explicit expansions are given as follows:*


$$\begin{array}{c} \{\begin{array}{ccc} Q_{5} & = & 1,\\ Q_{4} & = & \left(\alpha \rho +C+\alpha \eta +A\right)I^{*}+G+D+2\mu ,\\ Q_{3} & = & (\alpha \rho G+\alpha \rho D+CG+CD+\alpha \eta G+\alpha \eta D+\alpha \eta \mu +AG+AD+\mu A+\alpha \rho \mu +\mu C)I^{*}\\ & & +\left(\alpha^{2}\eta \rho +\alpha \eta C+\alpha \rho A+AC\right)I^{{*}^{2}}+DG+2\mu G+2\mu D+\mu^{2}-\iota AS_{1}^{*}-\iota CS_{2}^{*},\\ Q_{2} & = & (\alpha \rho DG+CDG+\alpha \rho \iota CS_{2}^{*}+\alpha \eta DG+\alpha \eta \mu G+\alpha \eta \mu D+ADG+\mu AG+\mu AD\\ & & +\alpha \rho \mu +\alpha \rho \mu G+\alpha \rho \mu D+\mu CG+\mu CD)I^{*}+(\alpha^{2}\eta \rho G+\alpha^{2}\eta \rho D+\alpha \eta CG+\alpha \eta CD\\ & & +\alpha \rho AG+\alpha \rho AD+ACG+ACD)I^{{*}^{2}}+2\mu DG+\left(D+G\right)\mu^{2}-(2\iota C^{2}S_{2}^{*}+\alpha \rho \iota AS_{1}^{*}\\ & & +\alpha \rho \iota CS_{2}^{*}+\iota ACS_{1}^{*}+\alpha \eta \iota CS_{2}^{*}+\iota ACS_{2}^{*})I^{*}-2\iota \mu A\iota ACS_{1}^{*}-2\iota \mu C\iota ACS_{2}^{*},\\ Q_{1} & = & (\alpha^{2}\rho \eta DG+\alpha \eta CDG+\alpha \rho ADG+ACDG-2\alpha \eta \iota C^{2}S_{2}^{*}-2\iota AC^{2}S_{2}^{*})I^{{*}^{2}}+(\alpha \eta \mu DG\\ & & +\mu ADG+\alpha \rho \mu DG-\alpha \eta \iota \mu CS_{2}^{*}-\iota \mu ACS_{2}^{*}-\iota \mu C^{2}S_{2}^{*}-\alpha \rho \iota \mu AS_{1}^{*}-\iota \mu ACS_{1}^{*}-\iota \mu C^{2}S_{2}^{*})I^{*}\\ & & +\mu^{2}DG-\iota \mu^{2}AS_{2}^{*}-\iota \mu^{2}CS_{2}^{*}. \end{array} \end{array}$$ (28)

*According to the Routh–Hurwitz criterion, the necessary and sufficient conditions for all characteristic roots of the equation to have negative real parts are* $Q_{1}>0,Q_{2}>0,Q_{3}>0,Q_{4}>0,$ *and* $Q_{4}Q_{3}>Q_{5}Q_{2},Q_{4}Q_{3}Q_{2}>Q_{5}Q_{2}^{2}+Q_{4}^{2}Q_{1}.$

*Because the model integrates an AI-enabled mechanism with a dual-channel knowledge dissemination mechanism, the nonlinear coupling among parameters makes the above inequalities difficult to simplify algebraically in an intuitive manner. Therefore, this study defines the following sufficient condition H*_*1*_:


$$\begin{array}{c} H_{1}:Q_{1}>0,Q_{2}>0,Q_{3}>0,Q_{4}>0, \end{array}$$

*and*


$$\begin{array}{c} Q_{4}Q_{3}>Q_{5}Q_{2},Q_{4}Q_{3}Q_{2}>Q_{5}Q_{2}^{2}+Q_{4}^{2}Q_{1} \end{array}$$

*Furthermore, the quartic equation* $Q_{5}h^{4}+Q_{4}h^{3}+Q_{3}h^{2}+Q_{2}h+Q_{1}=0$ *has all characteristic roots with negative real parts; therefore, the knowledge-present equilibrium is locally asymptotically stable, and* $I^{*},E^{*}$ *are locally asymptotically stable. The equilibrium values are therefore given by:*


$$\begin{array}{cc} S_{1}^{*} & =\frac{pB}{\alpha I^{*}\left(\eta +\varphi_{1}^{AI}k\right)+\mu },\\ S_{2}^{*} & =\frac{\left(1-p\right)B}{\alpha I^{*}\left(\rho +\varphi_{2}^{AI}k\right)+\mu },\\ E^{*} & =\frac{\left(\beta +\mu \right)I^{*}}{\iota },\\ I^{*} & =\frac{-b+\sqrt{\begin{array}{c} b^{2}-4\left(\alpha \eta +\alpha \varphi_{1}^{AI}k\right)\left(\alpha \rho +\alpha \varphi_{2}^{AI}k\right){\left[\left(\sigma +\iota +\mu \right)\left(\beta +\mu \right)\right]}^{2}\mu^{2}\\ +4\left(\alpha \eta +\alpha \varphi_{1}^{AI}k\right)\left(\alpha \rho +\alpha \varphi_{2}^{AI}k\right)\left(\sigma +\iota +\mu \right)\left(\beta +\mu \right)\\ \left[pB\alpha \varphi_{1}^{AI}k\iota +\left(1-p\right)B\alpha \varphi_{2}^{AI}k\iota \right]\mu \end{array}}}{2\left(\alpha \eta +\alpha \varphi_{1}^{AI}k\right)\left(\alpha \rho +\alpha \varphi_{2}^{AI}k\right)\left(\sigma +\iota +\mu \right)\left(\beta +\mu \right)}, \end{array}$$ (29)

*Where:*


$$\begin{array}{cc} b & =\left(\alpha \eta +\alpha \varphi_{1}^{AI}k\right)\cdot \left(\sigma +\iota +\mu \right)\left(\beta +\mu \right)\cdot \mu +\left(\alpha \rho +\alpha \varphi_{2}^{AI}k\right)\cdot \left(\sigma +\iota +\mu \right)\left(\beta +\mu \right)\cdot \mu \\ & \;-pB\alpha \varphi_{1}^{AI}k\iota \cdot \left(\alpha \rho +\alpha \varphi_{2}^{AI}k\right)-\left(1-p\right)B\alpha \varphi_{2}^{AI}k\iota \cdot \left(\alpha \eta +\alpha \varphi_{1}^{AI}k\right), \end{array}$$ (30)

*In the theoretical proof of Theorem 3, this study provides the sufficient condition H*_*1*_ *for the local asymptotic stability of* $\Lambda^{*}$. To verify the attainability of this condition in practical organizational management, this section conducts a numerical substitution test. Based on the benchmark parameter ranges specified in the model, it can be calculated that there exists a set of parameter values:


$$\begin{array}{c} \begin{array}{c} B=3.0,p=0.5,\alpha =0.7,\varphi_{1}^{AI}=0.7,\varphi_{2}^{AI}=0.5,k=1.5\\ \eta =0.2,\rho =0.2,\iota =0.7,\beta =0.1,\sigma =0.2,\mu =0.2. \end{array} \end{array}$$ (31)

*then we have:*


$$\begin{array}{c} \begin{array}{c} A=0.735,C=0.525,D=1.1,G=0.3,\\ I^{*}=18.599385032,S_{1}^{*}=0.091050014795,S_{2}^{*}=0.11934511947. \end{array} \end{array}$$ (32)

*Substituting* Eq. 31 *and* Eq. 32 *into* Eq. 28*, we obtain:*


$$\begin{array}{c} \left\{ \begin{array}{ccc} Q_{5} & = & 1,\\ Q_{4} & = & \left(\alpha \rho +C+\alpha \eta +A\right)I^{*}+G+D+2\mu =30.443052949>0,\\ Q_{3} & = & (\alpha \rho G+\alpha \rho D+CG+CD+\alpha \eta G+\alpha \eta D+\alpha \eta \mu +AG+AD+\mu A+\alpha \rho \mu +\mu C)I^{*}\\ & & +\left(\alpha^{2}\eta \rho +\alpha \eta C+\alpha \rho A+AC\right)I^{{*}^{2}}+DG+2\mu G+2\mu D+\mu^{2}-\iota AS_{1}^{*}-\iota CS_{2}^{*},\\ & = & 245.22623439,\\ Q_{2} & = & (\alpha \rho DG+CDG+\alpha \rho \iota CS_{2}^{*}+\alpha \eta DG+\alpha \eta \mu G+\alpha \eta \mu D+ADG+\mu AG+\mu AD\\ & & +\alpha \rho \mu +\alpha \rho \mu G+\alpha \rho \mu D+\mu CG+\mu CD)I^{*}+(\alpha^{2}\eta \rho G+\alpha^{2}\eta \rho D+\alpha \eta CG+\alpha \eta CD\\ & & +\alpha \rho AG+\alpha \rho AD+ACG+ACD)I^{{*}^{2}}+2\mu DG+\left(D+G\right)\mu^{2}-(2\iota C^{2}S_{2}^{*}+\alpha \rho \iota AS_{1}^{*}\\ & & +\alpha \rho \iota CS_{2}^{*}+\iota ACS_{1}^{*}+\alpha \eta \iota CS_{2}^{*}+\iota ACS_{2}^{*})I^{*}-2\iota \mu A\iota ACS_{1}^{*}-2\iota \mu C\iota ACS_{2}^{*},\\ & = & 295.19527904,\\ Q_{1} & = & (\alpha^{2}\rho \eta DG+\alpha \eta CDG+\alpha \rho ADG+ACDG-2\alpha \eta \iota C^{2}S_{2}^{*}-2\iota AC^{2}S_{2}^{*})I^{{*}^{2}}+(\alpha \eta \mu DG\\ & & +\mu ADG+\alpha \rho \mu DG-\alpha \eta \iota \mu CS_{2}^{*}-\iota \mu ACS_{2}^{*}-\iota \mu C^{2}S_{2}^{*}-\alpha \rho \iota \mu AS_{1}^{*}-\iota \mu ACS_{1}^{*}-\iota \mu C^{2}S_{2}^{*})I^{*}\\ & & +\mu^{2}DG-\iota \mu^{2}AS_{2}^{*}-\iota \mu^{2}CS_{2}^{*},\\ & = & 53.397283045, \end{array}\right. \end{array}$$ (33)

*From* Eq. 33*, it follows that:*


$$\begin{array}{c} \begin{array}{ccc} Q_{4}Q_{3}-Q_{5}Q_{2} & = & 30.443052949\times 245.22623439-295.19527904,\\ & = & 7170.239959>0,\\ Q_{4}Q_{3}Q_{2}-Q_{5}Q_{2}^{2}-Q_{4}^{2}Q_{1} & = & 30.443052949\times 245.22623439\times 295.19527904,\\ & & -1\times {\left(295.19527904\right)}^{2}-{\left(30.443052949\right)}^{2}\times 53.397283045,\\ & = & 2067133.4796>0. \end{array} \end{array}$$ (34)

*The calculation results show that, within a reasonable spatiotemporal domain of management parameters, condition H*_*1*_ *holds strictly. This confirms the theoretical derivation of Theorem 3: regardless of the knowledge distribution state of the system at the initial moment, under the sustained action of the AI-enabled mechanism, the system will eventually converge spontaneously to the stable knowledge-present equilibrium point* $\Lambda^{*}$. When R_*0*_ *> 1 and condition H*_*1*_ *is satisfied, the model is locally asymptotically stable. In addition to the parameter set considered in this study, other numerical solutions may also exist; this study only discusses the sufficient conditions under which the model satisfies local asymptotic stability.*

**Theorem 4.** *If R*_*0*_ *> 1, the knowledge-present equilibrium point of the system,* $\Lambda^{*}=\left(S_{1}^{*},S_{2}^{*},E^{*},I^{*},R^{*}\right)$*, is globally asymptotically stable.*

**Proof 4** *. In this study, the Lyapunov function is constructed as:*


$$\begin{array}{c} \;W\left(t\right)={\left[\left(S_{1}\left(t\right)-S_{1}^{*}\right)+\left(S_{2}\left(t\right)-S_{2}^{*}\right)+\left(E\left(t\right)-E^{*}\right)+\left(I\left(t\right)-I^{*}\right)+\left(R\left(t\right)-R^{*}\right)\right]}^{2}, \end{array}$$ (35)

*and*


$$\begin{array}{c} \begin{array}{ccc} W^{\prime }\left(t\right) & = & 2\left[\left(S_{1}\left(t\right)-S_{1}^{*}\right)+\left(S_{2}\left(t\right)-S_{2}^{*}\right)+\left(E\left(t\right)-E^{*}\right)+\left(I\left(t\right)-I^{*}\right)+\left(R\left(t\right)-R^{*}\right)\right]\\ & & \left[S_{1}^{{\;}^{\prime }}\left(t\right)+S_{2}^{{\;}^{\prime }}\left(t\right)+E^{{\;}^{\prime }}\left(t\right)+I^{{\;}^{\prime }}\left(t\right)+R^{{\;}^{\prime }}\left(t\right)\right]\\ & = & 2\left[\left(S_{1}\left(t\right)-S_{1}^{*}\right)+\left(S_{2}\left(t\right)-S_{2}^{*}\right)+\left(E\left(t\right)-E^{*}\right)+\left(I\left(t\right)-I^{*}\right)+\left(R\left(t\right)-R^{*}\right)\right]\\ & & \left[B-\mu S_{1}-\mu S_{2}-\mu E-\mu I-\mu R\right] \end{array}, \end{array}$$ (36)

*Since the equilibrium point* $\Lambda =\left(S_{1},S_{2},E,I,R\right)$ *exists, it follows that* $B-\mu S_{1}-\mu S_{2}-\mu E-\mu I-\mu R=0$*, i.e.,* $B=\mu S_{1}+\mu S_{2}+\mu E+\mu I+\mu R$.

*Thus,* Eq. 36 *can be calculated as:*


$$\begin{array}{c} \begin{array}{ccc} W^{\prime }\left(t\right) & = & 2\left[\left(S_{1}\left(t\right)-S_{1}^{*}\right)+\left(S_{2}\left(t\right)-S_{2}^{*}\right)+\left(E\left(t\right)-E^{*}\right)+\left(I\left(t\right)-I^{*}\right)+\left(R\left(t\right)-R^{*}\right)\right]\\ & & \left[\mu S_{1}^{*}+\mu S_{2}^{*}+\mu E^{*}+\mu I^{*}+\mu R^{*}-\mu S_{1}-\mu S_{2}-\mu E-\mu I-\mu R\right]\\ & = & -2\mu {\left[\left(S_{1}-S_{1}^{*}\right)+\left(S_{2}-S_{2}^{*}\right)+\left(E-E^{*}\right)+\left(I-I^{*}\right)+\left(R-R^{*}\right)\right]}^{2} \end{array}. \end{array}$$ (37)

*Moreover, the necessary and sufficient condition for* $W^{\prime }\left(t\right)=0$ *to hold is* $S_{1}\left(t\right)=S_{1}^{*},S_{2}\left(t\right)=S_{2}^{*},E\left(t\right)=E^{*},I\left(t\right)=I^{*},R\left(t\right)=R^{*}$. Therefore, based on the Lyapunov–LaSalle invariance principle, the knowledge-present equilibrium point of the system, $\Lambda^{*}=\left(S_{1}^{*},S_{2}^{*},E^{*},I^{*},R^{*}\right)$*, is globally asymptotically stable.*

## The optimal control model

### Optimal control model and its optimality conditions

Based on the knowledge dissemination model described above, employees who have not yet mastered the relevant knowledge$\left(S_{1}\right)$and employees with limited knowledge mastery*S*_2_can transition to employees with strong knowledge mastery(E), through AI-assisted learning, while employees with strong knowledge mastery(E), can further transition to knowledge-disseminating employees(I). To effectively promote dissemination within the organization, key parameters can be dynamically adjusted. This maximizes the number of knowledge-disseminating employees over a finite horizon while optimizing resource allocation. To this end, the four proportional constants $\alpha ,\varphi_{1}^{AI},\varphi_{2}^{AI},\iota$ in the model are respectively transformed into the control variables $\alpha \left(t\right),\varphi_{1}^{AI}\left(t\right),\varphi_{2}^{AI}\left(t\right),\iota \left(t\right)$. Here, α(t) denotes the organization’s adjustment of the effective knowledge contact intensity at time *t*. It reflects the level of effective contact established between employees and knowledge-disseminating employees through communication, collaboration, and knowledge sharing, and can be influenced through measures such as expert consultation, mentoring programs, cross-departmental exchanges, and knowledge-sharing activities. $\varphi_{1}^{AI}(t)$ denotes the efficiency with which employees who have not yet mastered *t*he relevant knowledge transition to the state of strong knowledge mastery under basic AI-assisted learning support. It can be adjusted by providing access to AI tools, foundational training, prompt templates, and internal knowledge bases. $\varphi_{2}^{AI}(t)$ denotes the efficiency with which employees with limited knowledge mastery transition to the state of strong knowledge mastery under advanced or personalized AI-assisted learning support. It can be adjusted through personalized learning pathways, task simulations, intelligent feedback, and expert review. ι(t) denotes the efficiency with which employees with strong knowledge mastery further transition into active knowledge-disseminating employees. It can be influenced by assigning knowledge-sharing tasks, establishing internal instructor or mentoring programs, allocating the necessary time for knowledge sharing, and implementing incentives for knowledge contributions. The above control variables represent the effective transformation levels generated by organizational interventions at different stages of knowledge transformation, rather than four mutually independent forms of direct financial investment. Through optimal control, a quantitative basis can be provided for enterprises to formulate phased and dynamic knowledge improvement strategies, thereby efficiently promoting the upgrading of employees’ knowledge structures and the cultivation of innovation capabilities under limited resources.

Therefore, the objective functional is defined as follows:


$$\begin{array}{c} \;J\left(\alpha ,\varphi_{1}^{AI},\varphi_{2}^{AI},\iota \right)=\int_{0}^{tf}\left[E\left(t\right)+I\left(t\right)-\frac{c_{1}}{2}\alpha^{2}\left(t\right)-\frac{c_{2}}{2}{\left(\varphi_{1}^{AI}\left(t\right)\right)}^{2}-\frac{c_{3}}{2}{\left(\varphi_{2}^{AI}\left(t\right)\right)}^{2}-\frac{c_{4}}{2}\iota^{2}\left(t\right)\right]dt. \end{array}$$ (38)

Subject to the following state system:


$$\begin{array}{c} \left\{ \begin{array}{ccc} \frac{dS_{1}}{dt} & = & pB-\alpha \left(t\right)\eta S_{1}I-\alpha \left(t\right)\varphi_{1}^{AI}\left(t\right)kS_{1}I-\mu S_{1},\\ \frac{dS_{2}}{dt} & = & \left(1-p\right)B-\alpha \left(t\right)\rho S_{2}I-\alpha \left(t\right)\varphi_{2}^{AI}\left(t\right)kS_{2}I-\mu S_{2},\\ \frac{dE}{dt} & = & \alpha \left(t\right)\varphi_{1}^{AI}\left(t\right)kS_{1}I+\alpha \left(t\right)\varphi_{2}^{AI}\left(t\right)kS_{2}I-\left(\sigma +\iota \left(t\right)+\mu \right)E,\\ \frac{dI}{dt} & = & \iota \left(t\right)E-\mu I-\beta I,\\ \frac{dR}{dt} & = & \alpha \left(t\right)\eta S_{1}I+\alpha \left(t\right)\rho S_{2}I+\sigma E+\beta I-\mu R. \end{array}\right. \end{array}$$ (39)

The initial conditions of System 39 satisfy:


$$\begin{array}{c} \;S_{1}\left(0\right)=S_{1,0},S_{2}\left(0\right)=S_{2,0},E\left(0\right)=E_{0},I\left(0\right)=I_{0},R\left(0\right)=R_{0}, \end{array}$$ (40)

where $\alpha \left(t\right),\varphi_{1}^{AI}\left(t\right),\varphi_{2}^{AI}\left(t\right),\iota \left(t\right)\in U.$


$$\begin{array}{c} U\overset{\Delta }{=}\left\{\left(\alpha ,\varphi_{1}^{AI},\varphi_{2}^{AI},\iota \right)|\alpha \left(t\right),\varphi_{1}^{AI}\left(t\right),\varphi_{2}^{AI}\left(t\right),\iota \left(t\right),0\leq \alpha \left(t\right),\varphi_{1}^{AI}\left(t\right),\varphi_{2}^{AI}\left(t\right),\iota \left(t\right)\leq 1,\forall t\in \left[0,tf\right]\right\}. \end{array}$$ (41)

Where *U* denotes the admissible control set. The control time interval ranges from 0 to $t_{f}$, and $c_{1},c_{2},c_{3},c_{4}$ are weight coefficients representing the control intensity and importance of the four control measures. Each control variable is normalized and constrained within a prescribed bounded interval to ensure that the admissible control set is nonempty and bounded. The quadratic control-cost terms in the objective functional are used to capture the convex costs associated with increasing control intensity. In practical applications, the upper bounds of the controls and the corresponding cost weights can be calibrated according to the organization’s AI platform capacity, personnel time availability, and budget constraints.

**Theorem 5.** *There exists an optimal control vector* $\left(\alpha^{*},{\varphi_{1}^{AI}}^{*},{\varphi_{2}^{AI}}^{*},\iota^{*}\right)\in U$ *such that the functional is established as follows:*


$$\begin{array}{c} \;J\left(\alpha^{*},\varphi_{1}^{AI*},\varphi_{2}^{AI*},\iota^{*}\right)=\max\left\{J\left(\alpha ,\varphi_{1}^{AI},\varphi_{2}^{AI},\iota \right):\left(\alpha ,\varphi_{1}^{AI},\varphi_{2}^{AI},\iota \right)\in U\right\}. \end{array}$$ (42)

**Proof 5***. Let* $X\left(t\right)={\left(S_{1}\left(t\right),S_{2}\left(t\right),E\left(t\right),I\left(t\right),R\left(t\right)\right)}^{T}$*, and*


$$\begin{array}{c} L\left(t;X\left(t\right),\alpha \left(t\right),\varphi_{1}^{AI}\left(t\right),\varphi_{2}^{AI}\left(t\right),\iota \left(t\right)\right)=E\left(t\right)+I\left(t\right)-\frac{c_{1}}{2}\alpha^{2}\left(t\right)-\frac{c_{2}}{2}{\left(\varphi_{1}^{AI}\left(t\right)\right)}^{2}-\frac{c_{3}}{2}{\left(\varphi_{2}^{AI}\left(t\right)\right)}^{2}-\frac{c_{4}}{2}\iota^{2}\left(t\right). \end{array}$$ (43)

*The existence of the optimal pair must satisfy the following conditions: (i) the set of control variables and state variables is nonempty; (ii) the control set U is convex and closed; (iii) the right-hand side of the state equations is bounded by a linear function of the state variables; (iv) the integrand of the objective functional is convex on U; and (v) there exist constants* $d_{1},d_{2}>0$ *and* ε>1 *such that the integrand of the objective functional satisfies:*


$$\begin{array}{c} \;-L\left(t;X\left(t\right),\alpha ;\varphi_{1}^{AI};\varphi_{2}^{AI};\iota \right)\geq d_{1}{\left({\left|\alpha \right|}^{2}+{\left|\varphi_{1}^{AI}\right|}^{2}+{\left|\varphi_{2}^{AI}\right|}^{2}+{\left|\iota \right|}^{2}\right)}^{\varepsilon /2\;}-d_{2}. \end{array}$$ (44)

*Conditions (i)–(iii) are clearly satisfied, and it remains only to prove conditions (iv) and (v). From the state system 39, the following inequality can be obtained:*


$$\begin{array}{c} \;S_{1}^{\prime }\leq pB,S_{2}^{\prime }\leq \left(1-p\right)B,E^{\prime }\leq \alpha \left(t\right)\varphi_{1}^{AI}\left(t\right)kS_{1}I+\alpha \left(t\right)\varphi_{2}^{AI}\left(t\right)kS_{2}I,I^{\prime }\leq \iota \left(t\right)E. \end{array}$$ (45)

*Therefore, condition (iv) holds. For any* t≥0*, there exists a constant M such that* |X(t)|≤M*; then,*


$$\begin{array}{c} \begin{array}{ccc} -L\left(t;X\left(t\right),\alpha ,\varphi_{1}^{AI},\varphi_{2}^{AI},\iota \right) & = & \left(c_{1}\alpha^{2}\left(t\right)+c_{2}\varphi_{1}^{AI2}\left(t\right)+c_{3}\varphi_{2}^{AI2}\left(t\right)+c_{4}\iota^{2}\left(t\right)\right)/2\;-E\left(t\right)-I\left(t\right),\\ & \geq & d_{1}{\left({\left|\alpha \right|}^{2}+{\left|\varphi_{1}^{AI}\right|}^{2}+{\left|\varphi_{2}^{AI}\right|}^{2}+{\left|\iota \right|}^{2}\right)}^{\varepsilon /2\;}-2M. \end{array} \end{array}$$ (46)

*Let* $d_{1}=\min\left\{\frac{c_{1}}{2},\frac{c_{2}}{2},\frac{c_{3}}{2},\frac{c_{4}}{2}\right\},d_{2}=2M,\varepsilon =2$*. Then condition (v) holds, and the optimal control is thereby achieved.*

**Theorem 6.** *For the optimal control pair* $\left(\alpha^{*},\varphi {{\;}_{1}^{AI}}^{*},\varphi {{\;}_{2}^{AI}}^{*},\iota^{*}\right)$ *of the state System 39, there exist adjoint variables* $\delta_{1},\delta_{2},\delta_{3},\delta_{4},\delta_{5}$ *satisfying:*


$$\begin{array}{c} \{\begin{array}{ccc} \frac{d\delta_{1}}{dt} & = & \left(\delta_{1}-\delta_{5}\right)\alpha \left(t\right)\eta I+\left(\delta_{1}-\delta_{3}\right)\alpha \left(t\right)\varphi_{1}^{AI}\left(t\right)kI+\delta_{1}\mu ,\\ \frac{d\delta_{2}}{dt} & = & \left(\delta_{2}-\delta_{5}\right)\alpha \left(t\right)\rho I+\left(\delta_{2}-\delta_{3}\right)\alpha \left(t\right)\varphi_{2}^{AI}\left(t\right)kI+\delta_{2}\mu ,\\ \frac{d\delta_{3}}{dt} & = & 1+\left(\delta_{3}-\delta_{5}\right)\sigma +\left(\delta_{3}-\delta_{4}\right)\iota \left(t\right)+\delta_{3}\mu ,\\ \frac{d\delta_{4}}{dt} & = & 1+\left(\delta_{1}-\delta_{5}\right)\alpha \left(t\right)\eta S_{1}+\left(\delta_{1}-\delta_{3}\right)\alpha \left(t\right)\varphi_{1}^{AI}\left(t\right)kS_{1}+\left(\delta_{2}-\delta_{5}\right)\alpha \left(t\right)\rho S_{2}\\ & & +\left(\delta_{2}-\delta_{3}\right)\alpha \left(t\right)\varphi_{2}^{AI}\left(t\right)kS_{2}+\left(\delta_{4}-\delta_{5}\right)\beta +\delta_{4}\mu ,\\ \frac{d\delta_{5}}{dt} & = & \delta_{5}\mu . \end{array} \end{array}$$ (47)

*With the boundary conditions:*


$$\begin{array}{c} \;\delta_{1}\left(tf\right)=\delta_{2}\left(tf\right)=\delta_{3}\left(tf\right)=\delta_{4}\left(tf\right)=\delta_{5}\left(tf\right)=0. \end{array}$$ (48)

*Moreover, the optimal control pair* $\left(\alpha^{*},\varphi_{1}^{AI*},\varphi_{2}^{AI*},\iota^{*}\right)$ *of the state System 39 can be given by:*


$$\begin{array}{c} \begin{array}{ccc} \alpha^{*}\left(t\right) & = & \min\left\{1,\max\left\{0,\frac{\left[\left(\delta_{1}-\delta_{3}\right)\varphi_{1}^{AI}\left(t\right)k+\left(\delta_{1}-\delta_{5}\right)\eta \right]S_{1}I+\left[\left(\delta_{2}-\delta_{3}\right)\varphi_{2}^{AI}\left(t\right)k+\left(\delta_{2}-\delta_{5}\right)\rho \right]S_{2}I}{c_{1}}\right\}\right\},\\ {\varphi_{1}^{AI}}^{*}\left(t\right) & = & \min\left\{1,\max\left\{0,\frac{\left(\delta_{1}-\delta_{3}\right)\alpha \left(t\right)kS_{1}I}{c_{2}}\right\}\right\},\\ {\varphi_{2}^{AI}}^{*}\left(t\right) & = & \min\left\{1,\max\left\{0,\frac{\left(\delta_{2}-\delta_{3}\right)\alpha \left(t\right)kS_{2}I}{c_{3}}\right\}\right\},\\ \iota^{*}\left(t\right) & = & \min\left\{1,\max\left\{0,\frac{\left(\delta_{3}-\delta_{4}\right)E}{c_{4}}\right\}\right\}. \end{array} \end{array}$$ (49)

**Proof 6** *. An augmented Hamiltonian with penalty terms is defined to obtain the optimality system and the expressions for the optimal control pair. The augmented Hamiltonian is given as follows:*


$$\begin{array}{cc} H & =-E\left(t\right)-I\left(t\right)+\frac{c_{1}}{2}\alpha^{2}\left(t\right)+\frac{c_{2}}{2}{\left(\varphi_{1}^{AI}\left(t\right)\right)}^{2}+\frac{c_{3}}{2}{\left(\varphi_{2}^{AI}\left(t\right)\right)}^{2}+\frac{c_{4}}{2}\iota^{2}\left(t\right)\\ & \;+\delta_{1}\left[pB-\alpha \left(t\right)\eta S_{1}I-\alpha \left(t\right)\varphi_{1}^{AI}\left(t\right)kS_{1}I-\mu S_{1}\right]+\delta_{2}[\left(1-p\right)B\\ & \;-\alpha \left(t\right)\rho S_{2}I-\alpha \left(t\right)\varphi_{2}^{AI}\left(t\right)kS_{2}I-\mu S_{2}]+\delta_{3}[\alpha \left(t\right)\varphi_{1}^{AI}\left(t\right)kS_{1}I\\ & \;+\alpha \left(t\right)\varphi_{2}^{AI}\left(t\right)kS_{2}I-\left(\sigma +\iota \left(t\right)+\mu \right)E]+\delta_{4}\left[\iota \left(t\right)E-\mu I-\beta I\right]\\ & \;+\delta_{5}\left[\alpha \left(t\right)\eta S_{1}I+\alpha \left(t\right)\rho S_{2}I+\sigma E+\beta I-\mu R\right]-\lambda_{11}\alpha \left(t\right)-\lambda_{12}\left(1-\alpha \left(t\right)\right)\\ & \;-\lambda_{21}\varphi_{1}\left(t\right)-\lambda_{22}\left(1-\varphi_{1}\left(t\right)\right)-\lambda_{31}\varphi_{2}\left(t\right)-\lambda_{32}\left(1-\varphi_{2}\left(t\right)\right)-\lambda_{41}\iota \left(t\right)-\lambda_{42}\left(1-\iota \left(t\right)\right). \end{array}$$ (50)

*Where the penalty terms satisfy* $\lambda_{ij}\left(t\right)\geq 0,$ *Under the optimal control condition,* $\alpha^{*}$ *satisfies* $\lambda_{11}\left(t\right)\alpha \left(t\right)=\lambda_{12}\left(t\right)\left(1-\alpha \left(t\right)\right)=0$*; under the optimal control condition,* $\varphi_{1}^{AI*}$ *satisfies* $\lambda_{21}\left(t\right)\varphi_{1}\left(t\right)=\lambda_{22}\left(t\right)\left(1-\varphi_{1}\left(t\right)\right)=0$*; under the optimal control condition,* $\varphi_{2}^{AI*}$ *satisfies* $\lambda_{31}\left(t\right)\varphi_{2}^{AI}\left(t\right)=\lambda_{32}\left(t\right)\left(1-\varphi_{2}^{AI}\left(t\right)\right)=0$*; and under the optimal control condition,* $\iota^{*}$ *satisfies* $\lambda_{41}\left(t\right)\iota \left(t\right)=\lambda_{42}\left(t\right)\left(1-\iota \left(t\right)\right)=0$.

*Based on Pontryagin’s Maximum Principle, the adjoint system is:*


$$\begin{array}{c} \;\frac{d\delta_{1}}{dt}=-\frac{\partial H}{\partial S_{1}},\frac{d\delta_{2}}{dt}=-\frac{\partial H}{\partial S_{2}},\frac{d\delta_{3}}{dt}=-\frac{\partial H}{\partial E},\frac{d\delta_{4}}{dt}=-\frac{\partial H}{\partial I},\frac{d\delta_{5}}{dt}=-\frac{\partial H}{\partial R}. \end{array}$$ (51)

*The boundary conditions of the adjoint system are:*


$$\begin{array}{c} \;\delta_{1}\left(tf\right)=\delta_{2}\left(tf\right)=\delta_{3}\left(tf\right)=\delta_{4}\left(tf\right)=\delta_{5}\left(tf\right)=0. \end{array}$$ (52)

*Taking* $\alpha^{*}$ *as an example, the optimal control is given as follows:*


$$\begin{array}{cc} \frac{\partial H}{\partial \alpha } & =c_{1}\alpha \left(t\right)+\delta_{1}\left[-\eta S_{1}I-\varphi_{1}^{AI}\left(t\right)kS_{1}I\right]+\delta_{2}\left[-\rho S_{2}I-\varphi_{2}^{AI}\left(t\right)kS_{2}I\right]\\ & \;+\delta_{3}\left[\varphi_{1}^{AI}\left(t\right)kS_{1}I+\varphi_{2}^{AI}\left(t\right)kS_{2}I\right]+\delta_{5}\left(\eta S_{1}I+\rho S_{2}I\right)-\lambda_{11}+\lambda_{12},\\ & =0. \end{array}$$ (53)

*The optimal control formula is given by:*


$$\begin{array}{c} \alpha^{*}=\frac{1}{c_{1}}\left[\left(\delta_{1}-\delta_{3}\right)\varphi_{1}^{AI}\left(t\right)k+\left(\delta_{1}-\delta_{5}\right)\eta \right]S_{1}I+\left[\left(\delta_{2}-\delta_{3}\right)\varphi_{2}^{AI}\left(t\right)k+\left(\delta_{2}-\delta_{5}\right)\rho \right]S_{2}I+\lambda_{11}-\lambda_{12}. \end{array}$$ (54)

*To obtain the final optimal control formula without considering* $\lambda_{11}$ *and* $\lambda_{12}$*, the following three cases need to be considered:*

(1) *The first case is that* $\lambda_{11}\left(t\right)=\lambda_{12}\left(t\right)=0$ *holds on the set*$\left\{t|0<\alpha^{*}\left(t\right)<1\right\}$*; then the expression of the optimal control parameter is:*

$$\begin{array}{c} \;\alpha^{*}\left(t\right)=\frac{1}{c_{1}}\left[\left[\left(\delta_{1}-\delta_{3}\right)\varphi_{1}^{AI}\left(t\right)k+\left(\delta_{1}-\delta_{5}\right)\eta \right]S_{1}I+\left[\left(\delta_{2}-\delta_{3}\right)\varphi_{2}^{AI}\left(t\right)k+\left(\delta_{2}-\delta_{5}\right)\rho \right]S_{2}I\right]. \end{array}$$ (55)

(2) *The second case is that* $\lambda_{11}\left(t\right)=0$ *holds on the set* $\left\{t|\alpha^{*}\left(t\right)=1\right\}$*; then the expression of the optimal control parameter is:*

$$\begin{array}{c} 1=\alpha^{*}\left(t\right)=\frac{1}{c_{1}}\left[\left[\left(\delta_{1}-\delta_{3}\right)\varphi_{1}^{AI}\left(t\right)k+\left(\delta_{1}-\delta_{5}\right)\eta \right]S_{1}I+\left[\left(\delta_{2}-\delta_{3}\right)\varphi_{2}^{AI}\left(t\right)k+\left(\delta_{2}-\delta_{5}\right)\rho \right]S_{2}I-\lambda_{12}\right]. \end{array}$$ (56)

*For* $\lambda_{12}\left(t\right)\geq 0$*, the result is:*


$$\begin{array}{c} \;\frac{1}{c_{1}}\left[\left[\left(\delta_{1}-\delta_{3}\right)\varphi_{1}^{AI}\left(t\right)k+\left(\delta_{1}-\delta_{5}\right)\eta \right]S_{1}I+\left[\left(\delta_{2}-\delta_{3}\right)\varphi_{2}^{AI}\left(t\right)k+\left(\delta_{2}-\delta_{5}\right)\rho \right]S_{2}I\right]\geq 1. \end{array}$$ (57)

(3) *The third case is that* $\lambda_{12}\left(t\right)=0$ *holds on the set* $\left\{t|\alpha^{*}\left(t\right)=0\right\}$*; then the optimal control expression is:*

$$\begin{array}{c} 0=\alpha^{*}\left(t\right)=\frac{1}{c_{1}}\left[\left[\left(\delta_{1}-\delta_{3}\right)\varphi_{1}^{AI}\left(t\right)k+\left(\delta_{1}-\delta_{5}\right)\eta \right]S_{1}I+\left[\left(\delta_{2}-\delta_{3}\right)\varphi_{2}^{AI}\left(t\right)k+\left(\delta_{2}-\delta_{5}\right)\rho \right]S_{2}I+\lambda_{11}\right]. \end{array}$$ (58)

*Based on the above cases, the final optimal control formula for* $\alpha^{*}\left(t\right)$ *is:*


$$\begin{array}{c} \alpha^{*}\left(t\right)=\min\left\{1,\max\left\{0,\frac{\left[\left(\delta_{1}-\delta_{3}\right)\varphi_{1}^{AI}\left(t\right)k+\left(\delta_{1}-\delta_{5}\right)\eta \right]S_{1}I+\left[\left(\delta_{2}-\delta_{3}\right)\varphi_{2}^{AI}\left(t\right)k+\left(\delta_{2}-\delta_{5}\right)\rho \right]S_{2}I}{c_{1}}\right\}\right\}. \end{array}$$ (59)

*Similarly, the final optimal control formula for* $\varphi {{\;}_{1}^{AI}}^{*}\left(t\right)$ *is:*


$$\begin{array}{c} {\varphi_{1}^{AI}}^{*}\left(t\right)=\min\left\{1,\max\left\{0,\frac{\left(\delta_{1}-\delta_{3}\right)\alpha \left(t\right)kS_{1}I}{c_{2}}\right\}\right\}. \end{array}$$ (60)

*The final optimal control formula for* $\varphi_{2}^{AI*}\left(t\right)$ *is:*


$$\begin{array}{c} \;{\varphi_{2}^{AI}}^{*}\left(t\right)=\min\left\{1,\max\left\{0,\frac{\left(\delta_{2}-\delta_{3}\right)\alpha \left(t\right)kS_{2}I}{c_{3}}\right\}\right\}. \end{array}$$ (61)

*The final optimal control formula for* $\iota^{*}\left(t\right)$ *is:*


$$\begin{array}{c} \iota^{*}\left(t\right)=\min\left\{1,\max\left\{0,\frac{\left(\delta_{3}-\delta_{4}\right)E}{c_{4}}\right\}\right\}. \end{array}$$ (62)

*Thus, the obtained optimal control system consists of the state System 39, with initial conditions* $S_{1}\left(0\right),S_{2}\left(0\right),E\left(0\right),I\left(0\right),R\left(0\right)$*, and the adjoint System 47, together with the boundary conditions and optimality conditions. The optimal control system is given as follows:*


$$\begin{array}{c} \{\begin{array}{ccc} \frac{dS_{1}}{dt} & = & pB-\min\left\{1,\max\left\{0,\frac{\left[\left(\delta_{1}-\delta_{3}\right)\varphi_{1}^{AI}\left(t\right)k+\left(\delta_{1}-\delta_{5}\right)\eta \right]S_{1}I+\left[\left(\delta_{2}-\delta_{3}\right)\varphi_{2}^{AI}\left(t\right)k+\left(\delta_{2}-\delta_{5}\right)\rho \right]S_{2}I}{c_{1}}\right\}\right\}\left(t\right)\eta S_{1}I\\ & & -\min\left\{1,\max\left\{0,\frac{\left[\left(\delta_{1}-\delta_{3}\right)\varphi_{1}^{AI}\left(t\right)k+\left(\delta_{1}-\delta_{5}\right)\eta \right]S_{1}I+\left[\left(\delta_{2}-\delta_{3}\right)\varphi_{2}^{AI}\left(t\right)k+\left(\delta_{2}-\delta_{5}\right)\rho \right]S_{2}I}{c_{1}}\right\}\right\}\left(t\right)\\ & & \min\left\{1,\max\left\{0,\frac{\left(\delta_{1}-\delta_{3}\right)\alpha \left(t\right)kS_{1}I}{c_{2}}\right\}\right\}\left(t\right)kS_{1}I-\mu S_{1},\\ \frac{dS_{2}}{dt} & = & \left(1-p\right)B-\min\left\{1,\max\left\{0,\frac{\left[\left(\delta_{1}-\delta_{3}\right)\varphi_{1}^{AI}\left(t\right)k+\left(\delta_{1}-\delta_{5}\right)\eta \right]S_{1}I+\left[\left(\delta_{2}-\delta_{3}\right)\varphi_{2}^{AI}\left(t\right)k+\left(\delta_{2}-\delta_{5}\right)\rho \right]S_{2}I}{c_{1}}\right\}\right\}\left(t\right)\rho S_{2}I\\ & & -\min\left\{1,\max\left\{0,\frac{\left[\left(\delta_{1}-\delta_{3}\right)\varphi_{1}^{AI}\left(t\right)k+\left(\delta_{1}-\delta_{5}\right)\eta \right]S_{1}I+\left[\left(\delta_{2}-\delta_{3}\right)\varphi_{2}^{AI}\left(t\right)k+\left(\delta_{2}-\delta_{5}\right)\rho \right]S_{2}I}{c_{1}}\right\}\right\}\left(t\right)\\ & & \min\left\{1,\max\left\{0,\frac{\left(\delta_{2}-\delta_{3}\right)\alpha \left(t\right)kS_{2}I}{c_{3}}\right\}\right\}\left(t\right)kS_{2}I-\mu S_{2},\\ \frac{dE}{dt} & = & \min\left\{1,\max\left\{0,\frac{\left[\left(\delta_{1}-\delta_{3}\right)\varphi_{1}^{AI}\left(t\right)k+\left(\delta_{1}-\delta_{5}\right)\eta \right]S_{1}I+\left[\left(\delta_{2}-\delta_{3}\right)\varphi_{2}^{AI}\left(t\right)k+\left(\delta_{2}-\delta_{5}\right)\rho \right]S_{2}I}{c_{1}}\right\}\right\}\left(t\right)\\ & & \min\left\{1,\max\left\{0,\frac{\left(\delta_{1}-\delta_{3}\right)\alpha \left(t\right)kS_{1}I}{c_{2}}\right\}\right\}\left(t\right)kS_{1}I\\ & & +\min\left\{1,\max\left\{0,\frac{\left[\left(\delta_{1}-\delta_{3}\right)\varphi_{1}^{AI}\left(t\right)k+\left(\delta_{1}-\delta_{5}\right)\eta \right]S_{1}I+\left[\left(\delta_{2}-\delta_{3}\right)\varphi_{2}^{AI}\left(t\right)k+\left(\delta_{2}-\delta_{5}\right)\rho \right]S_{2}I}{c_{1}}\right\}\right\}\left(t\right)\\ & & \min\left\{1,\max\left\{0,\frac{\left(\delta_{2}-\delta_{3}\right)\alpha \left(t\right)kS_{2}I}{c_{3}}\right\}\right\}\left(t\right)kS_{2}I-\left(\sigma +\min\left\{1,\max\left\{0,\frac{\left(\delta_{3}-\delta_{4}\right)E}{c_{4}}\right\}\right\}+\mu \right)E,\\ \frac{dI}{dt} & = & \min\left\{1,\max\left\{0,\frac{\left(\delta_{3}-\delta_{4}\right)E}{c_{4}}\right\}\right\}\left(t\right)E-\beta I-\mu I,\\ \frac{dR}{dt} & = & \min\left\{1,\max\left\{0,\frac{\left[\left(\delta_{1}-\delta_{3}\right)\varphi_{1}^{AI}\left(t\right)k+\left(\delta_{1}-\delta_{5}\right)\eta \right]S_{1}I+\left[\left(\delta_{2}-\delta_{3}\right)\varphi_{2}^{AI}\left(t\right)k+\left(\delta_{2}-\delta_{5}\right)\rho \right]S_{2}I}{c_{1}}\right\}\right\}\left(t\right)\eta S_{1}I\\ & & +\min\left\{1,\max\left\{0,\frac{\left[\left(\delta_{1}-\delta_{3}\right)\varphi_{1}^{AI}\left(t\right)k+\left(\delta_{1}-\delta_{5}\right)\eta \right]S_{1}I+\left[\left(\delta_{2}-\delta_{3}\right)\varphi_{2}^{AI}\left(t\right)k+\left(\delta_{2}-\delta_{5}\right)\rho \right]S_{2}I}{c_{1}}\right\}\right\}\left(t\right)\rho S_{2}I\\ & & +\sigma E+\beta I-\mu R,\\ \frac{d\delta_{1}}{dt} & = & \left(\delta_{1}-\delta_{5}\right)\min\left\{1,\max\left\{0,\frac{\left[\left(\delta_{1}-\delta_{3}\right)\varphi_{1}^{AI}\left(t\right)k+\left(\delta_{1}-\delta_{5}\right)\eta \right]S_{1}I+\left[\left(\delta_{2}-\delta_{3}\right)\varphi_{2}^{AI}\left(t\right)k+\left(\delta_{2}-\delta_{5}\right)\rho \right]S_{2}I}{c_{1}}\right\}\right\}\left(t\right)\eta I\\ & & +\left(\delta_{1}-\delta_{3}\right)\min\left\{1,\max\left\{0,\frac{\left[\left(\delta_{1}-\delta_{3}\right)\varphi_{1}^{AI}\left(t\right)k+\left(\delta_{1}-\delta_{5}\right)\eta \right]S_{1}I+\left[\left(\delta_{2}-\delta_{3}\right)\varphi_{2}^{AI}\left(t\right)k+\left(\delta_{2}-\delta_{5}\right)\rho \right]S_{2}I}{c_{1}}\right\}\right\}\left(t\right)\\ & & \min\left\{1,\max\left\{0,\frac{\left(\delta_{1}-\delta_{3}\right)\alpha \left(t\right)kS_{1}I}{c_{2}}\right\}\right\}\left(t\right)kI+\delta_{1}\mu ,\\ \frac{d\delta_{2}}{dt} & = & \left(\delta_{2}-\delta_{5}\right)\min\left\{1,\max\left\{0,\frac{\left[\left(\delta_{1}-\delta_{3}\right)\varphi_{1}^{AI}\left(t\right)k+\left(\delta_{1}-\delta_{5}\right)\eta \right]S_{1}I+\left[\left(\delta_{2}-\delta_{3}\right)\varphi_{2}^{AI}\left(t\right)k+\left(\delta_{2}-\delta_{5}\right)\rho \right]S_{2}I}{c_{1}}\right\}\right\}\left(t\right)\rho I\\ & & +\left(\delta_{2}-\delta_{3}\right)\min\left\{1,\max\left\{0,\frac{\left[\left(\delta_{1}-\delta_{3}\right)\varphi_{1}^{AI}\left(t\right)k+\left(\delta_{1}-\delta_{5}\right)\eta \right]S_{1}I+\left[\left(\delta_{2}-\delta_{3}\right)\varphi_{2}^{AI}\left(t\right)k+\left(\delta_{2}-\delta_{5}\right)\rho \right]S_{2}I}{c_{1}}\right\}\right\}\left(t\right)\\ & & \min\left\{1,\max\left\{0,\frac{\left(\delta_{2}-\delta_{3}\right)\alpha \left(t\right)kS_{2}I}{c_{3}}\right\}\right\}\left(t\right)kI+\delta_{2}\mu ,\\ \frac{d\delta_{3}}{dt} & = & 1+\left(\delta_{3}-\delta_{5}\right)\sigma +\left(\delta_{3}-\delta_{4}\right)\min\left\{1,\max\left\{0,\frac{\left(\delta_{3}-\delta_{4}\right)E}{c_{4}}\right\}\right\}\left(t\right)+\delta_{3}\mu ,\\ \frac{d\delta_{4}}{dt} & = & 1+\left(\delta_{1}-\delta_{5}\right)\min\left\{1,\max\left\{0,\frac{\left[\left(\delta_{1}-\delta_{3}\right)\varphi_{1}^{AI}\left(t\right)k+\left(\delta_{1}-\delta_{5}\right)\eta \right]S_{1}I+\left[\left(\delta_{2}-\delta_{3}\right)\varphi_{2}^{AI}\left(t\right)k+\left(\delta_{2}-\delta_{5}\right)\rho \right]S_{2}I}{c_{1}}\right\}\right\}\left(t\right)\eta S_{1}\\ & & +\left(\delta_{1}-\delta_{3}\right)\min\left\{1,\max\left\{0,\frac{\left[\left(\delta_{1}-\delta_{3}\right)\varphi_{1}^{AI}\left(t\right)k+\left(\delta_{1}-\delta_{5}\right)\eta \right]S_{1}I+\left[\left(\delta_{2}-\delta_{3}\right)\varphi_{2}^{AI}\left(t\right)k+\left(\delta_{2}-\delta_{5}\right)\rho \right]S_{2}I}{c_{1}}\right\}\right\}\left(t\right)\\ & & \min\left\{1,\max\left\{0,\frac{\left(\delta_{1}-\delta_{3}\right)\alpha \left(t\right)kS_{1}I}{c_{2}}\right\}\right\}\left(t\right)kS_{1}\\ & & +\left(\delta_{2}-\delta_{5}\right)\min\left\{1,\max\left\{0,\frac{\left[\left(\delta_{1}-\delta_{3}\right)\varphi_{1}^{AI}\left(t\right)k+\left(\delta_{1}-\delta_{5}\right)\eta \right]S_{1}I+\left[\left(\delta_{2}-\delta_{3}\right)\varphi_{2}^{AI}\left(t\right)k+\left(\delta_{2}-\delta_{5}\right)\rho \right]S_{2}I}{c_{1}}\right\}\right\}\left(t\right)\rho S_{2}\\ & & +\left(\delta_{2}-\delta_{3}\right)\min\left\{1,\max\left\{0,\frac{\left[\left(\delta_{1}-\delta_{3}\right)\varphi_{1}^{AI}\left(t\right)k+\left(\delta_{1}-\delta_{5}\right)\eta \right]S_{1}I+\left[\left(\delta_{2}-\delta_{3}\right)\varphi_{2}^{AI}\left(t\right)k+\left(\delta_{2}-\delta_{5}\right)\rho \right]S_{2}I}{c_{1}}\right\}\right\}\left(t\right)\\ & & \min\left\{1,\max\left\{0,\frac{\left(\delta_{2}-\delta_{3}\right)\alpha \left(t\right)kS_{2}I}{c_{3}}\right\}\right\}\left(t\right)kS_{2}+\left(\delta_{4}-\delta_{5}\right)\beta +\delta_{4}\mu ,\\ \frac{d\delta_{5}}{dt} & = & \delta_{5}\mu . \end{array} \end{array}$$ (63)

*And*


$$\begin{array}{c} \begin{array}{c} S_{1}\left(0\right)=S_{1,0},S_{2}\left(0\right)=S_{2,0},E\left(0\right)=E_{0},I\left(0\right)=I_{0},R\left(0\right)=R_{0},\\ \delta_{1}\left(tf\right)=\delta_{2}\left(tf\right)=\delta_{3}\left(tf\right)=\delta_{4}\left(tf\right)=\delta_{5}\left(tf\right)=0. \end{array} \end{array}$$ (64)

### Organizational operationalization and practical implementation of the control variables

The above four control variables should be interpreted as standardized intervention intensities regulating different knowledge-state transition processes, rather than as direct financial investments by enterprises in AI platforms, training programs, or incentive schemes. Specifically, α(t) represents the effective intensity of knowledge contact between employees and knowledge-disseminating employees; $\varphi_{1}^{AI}\left(t\right)$ and $\varphi_{2}^{AI}\left(t\right)$ characterize the regulatory effects of foundational and advanced AI-assisted learning support, respectively, on the knowledge transformation process; and ι(t) captures the regulatory effect on the transition of employees from the strong knowledge mastery state to the active knowledge dissemination state. In practical organizational contexts, these control variables can be influenced through mechanisms such as mentorship and expert consultation, cross-departmental knowledge exchange, AI tool access and training, internal knowledge repositories, personalized learning support, knowledge-sharing tasks, and incentives for knowledge contribution. The organizational implications and major operationalization approaches of each control variable are summarized in Table 4.

**Table 4:** Organizational implications and practical operationalization of control variables.

Control variable	Practical organizational meaning	Main observable information and implementation approaches
α(t)	Effective knowledge contact intensity between employees and knowledge-disseminating employees.	Records of mentorship or expert consultations, cross-departmental exchanges, and knowledge-sharing activities; can be regulated through mentorship programs, expert consultation mechanisms, and organizational knowledge exchange arrangements.
$\varphi_{1}^{AI}\left(t\right)$	Regulatory level of the transition from *S*_1_ to the strong knowledge mastery state under foundational AI-assisted learning support.	AI tool usage records, foundational training records, knowledge repository access logs, and knowledge assessment records; can be regulated through AI tool access permissions, foundational training programs, prompt templates, and internal knowledge repositories.
$\varphi_{2}^{AI}\left(t\right)$	Regulatory level of the transition from *S*_2_ to the strong knowledge mastery state under advanced or personalized AI-assisted learning support.	Personalized learning records, task simulation records, intelligent feedback records, and competency assessment records; can be regulated through personalized learning pathways, intelligent feedback mechanisms, and expert review processes.
ι(t)	Regulatory level of the transition from the strong knowledge mastery state to the active knowledge dissemination state.	Internal training records, mentorship activity records, knowledge-sharing records, and knowledge contribution records; can be regulated through knowledge-sharing tasks, mentorship programs, dedicated knowledge-sharing time allocation, and incentives for knowledge contribution.

It should be noted that the above observable records primarily provide information for the empirical calibration of control variables, rather than serving as direct measurements of individual control variables. In particular, single indicators, such as the frequency of AI platform usage or the actual proportion of transitions between specific knowledge states, should not be directly equated with any particular control variable in the model, because knowledge-state transitions in the model are generally jointly determined by multiple parameters. In practical applications, the feasible upper bounds of the control variables and the cost weights in the objective functional can be calibrated according to organizational conditions, such as AI platform capacity, expert availability, employee workload, training resources, and managerial budget constraints. Accordingly, the optimal control trajectories derived from the model should be interpreted as indicating the relative priorities and intervention intensities of different intervention channels over time, rather than as fixed resource allocation proportions that can be directly transferred across different organizational settings.

## Numerical simulations

### Simulation settings and parameter descriptions

To validate the theoretical analysis and investigate AI impacts on dissemination dynamics, the state equations are solved using the Runge–Kutta method. Given that existing studies have not yet provided a unified range of values for the relevant parameters, the parameter settings in this study draw on prior research on dissemination dynamics and cellular automata models [46,47]. In addition, the parameters are specified by jointly considering the stability conditions of the model and the value range of the basic reproduction number, so that they fall within reasonable intervals and thereby ensure the organizational interpretability and numerical stability of the system. During the simulation process, unless otherwise stated, all other parameters are kept unchanged, so as to analyze the influence of variations in a single parameter or multiple parameters on the evolutionary behavior of the system.

### Numerical simulation of system stability

This section verifies the feasibility of the theoretical analytical results of System 1. To validate the local and global stability of the knowledge-free equilibrium $\Lambda_{0}$ established in Theorem 1 and Theorem 2, let the initial system parameters be $B=2.0,p=0.6,\alpha =0.08,\varphi_{1}^{AI}=0.30,\rho =0.15,k=1.0,\eta =0.10,\varphi_{2}^{AI}=0.15,\iota =0.25,\beta =0.28,\sigma =0.35,\mu =0.10$. The calculation yields *R*_0_ = 0.16578972079 < 1. Fig 4 illustrates the stable state of the model at the knowledge-free equilibrium and the temporal evolution of the density of each compartment in the system with respect to time *t*. As shown in Fig 4, under the selected initial conditions, the densities of employees with strong knowledge mas*t*ery E(t), knowledge-disseminating employees I(t), and knowledge-exited employees R(t) decrease monotonically after brief fluctuations and eventually converge to zero. Meanwhile, the densities of employees who have not yet mastered the knowledge $S_{1}\left(t\right)$ and employees with limited knowledge mastery $S_{2}\left(t\right)$ gradually increase until they reach a stable state. Specifically, employees who have not yet mastered the knowledge $S_{1}\left(t\right)$ increase sharply within a short period, reach equilibrium, and then tend toward stability, whereas employees with limited knowledge mastery $S_{2}\left(t\right)$exhibit only a slight increase before eventually stabilizing. It can therefore be concluded that, when the basic reproduction number is less than 1, the knowledge-free equilibrium $\Lambda_{0}$ is stable, thereby verifying Theorem 1 and Theorem 2. Knowledge cannot form a persistent diffusion process within the network, and the system will eventually return to a stagnant state in which all employees remain either knowledge-free or at a low level of knowledge mastery.

**Fig 4:**
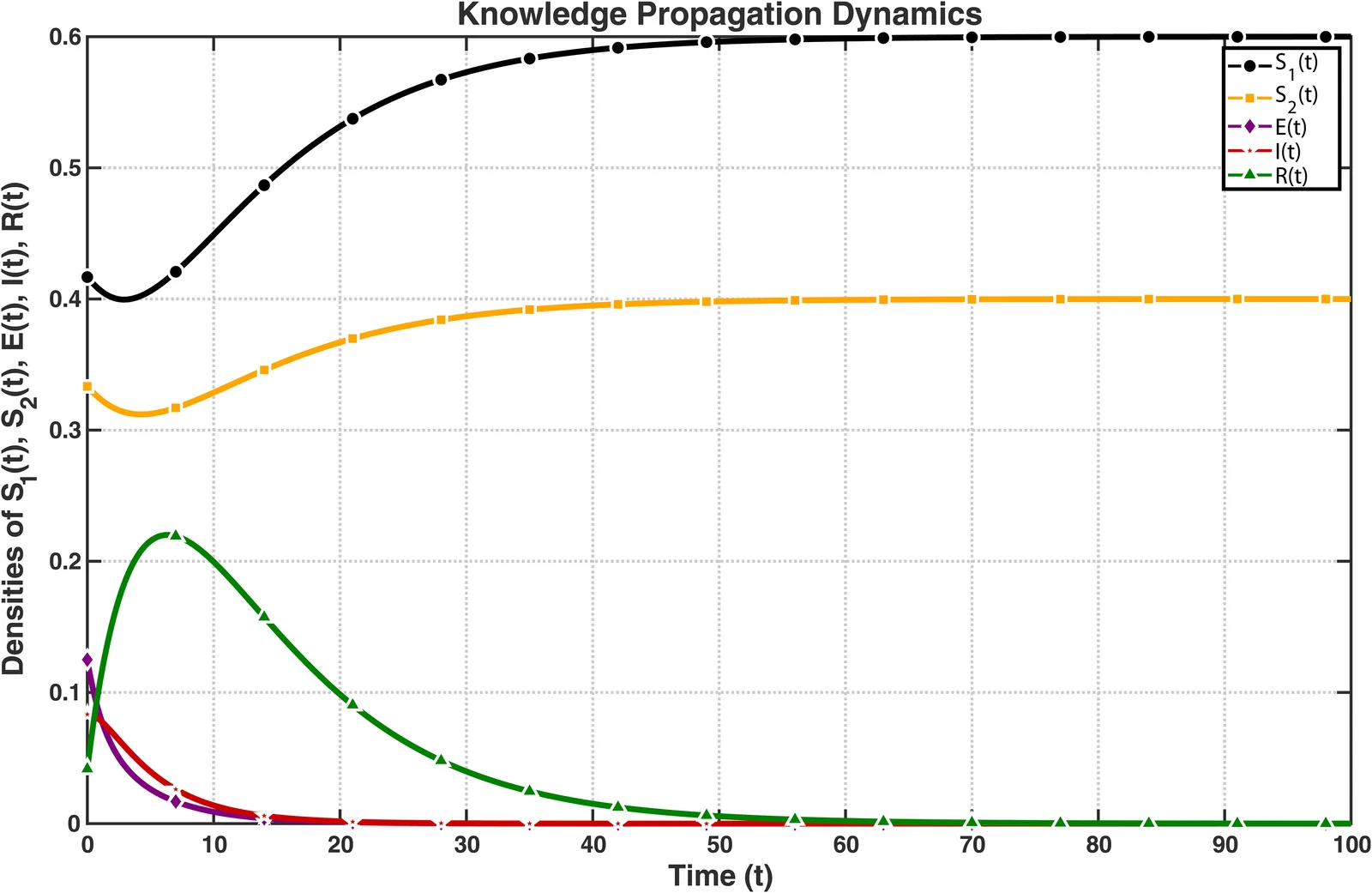
Stability of the equilibrium point $\Lambda_{0}$.

To verify the local and global asymptotic stability of the knowledge-present equilibrium $\Lambda_{1}$ established in Theorem 3 and Theorem 4, let $B=3.0,p=0.5,\alpha =0.7,\varphi_{1}^{AI}=0.7,k=1.5,\eta =0.2,\rho =0.2,\varphi_{2}^{AI}=0.5,\iota =0.7,\beta =0.1,\sigma =0.2,\mu =0.2$. The calculation gives *R*_0_ = 1.0056016319 > 1. As illustrated in Fig 5, as time evolves, the densities of employees who have not yet mastered the knowledge $S_{1}\left(t\right)$ and employees with limited knowledge mastery $S_{2}\left(t\right)$ decrease rapidly, reflecting the strong penetrative capacity of knowledge within the enterprise. The population of employees with strong knowledge mastery E(t) is partly derived from employees who have not yet mastered the knowledge $S_{1}\left(t\right)$ and employees with limited knowledge mastery $S_{2}\left(t\right)$; another portion of employees with strong knowledge mastery, after internalizing and assimilating the knowledge, transforms into knowledge-disseminating employees. Therefore, in the figure, the population of employees with strong knowledge mastery overall exhibits a more gradual downward trend compared with employees who have not yet mastered the knowledge and employees with limited knowledge mastery. The populations of knowledge-disseminating employees I(t) and knowledge-exited employees R(t) gradually increase and tend toward stability; in particular, the population of knowledge-disseminating employees I(t) first rises and then declines before eventually stabilizing. Ultimately, the system reaches a stable state at the equilibrium point. It can therefore be concluded that, when the basic reproduction number is greater than 1, the knowledge-present equilibrium $\Lambda_{1}$ is stable, thereby verifying Theorem 3 and Theorem 4. This indicates that knowledge can cross the dissemination threshold and form a long-term stable dissemination pattern within the enterprise.

**Fig 5:**
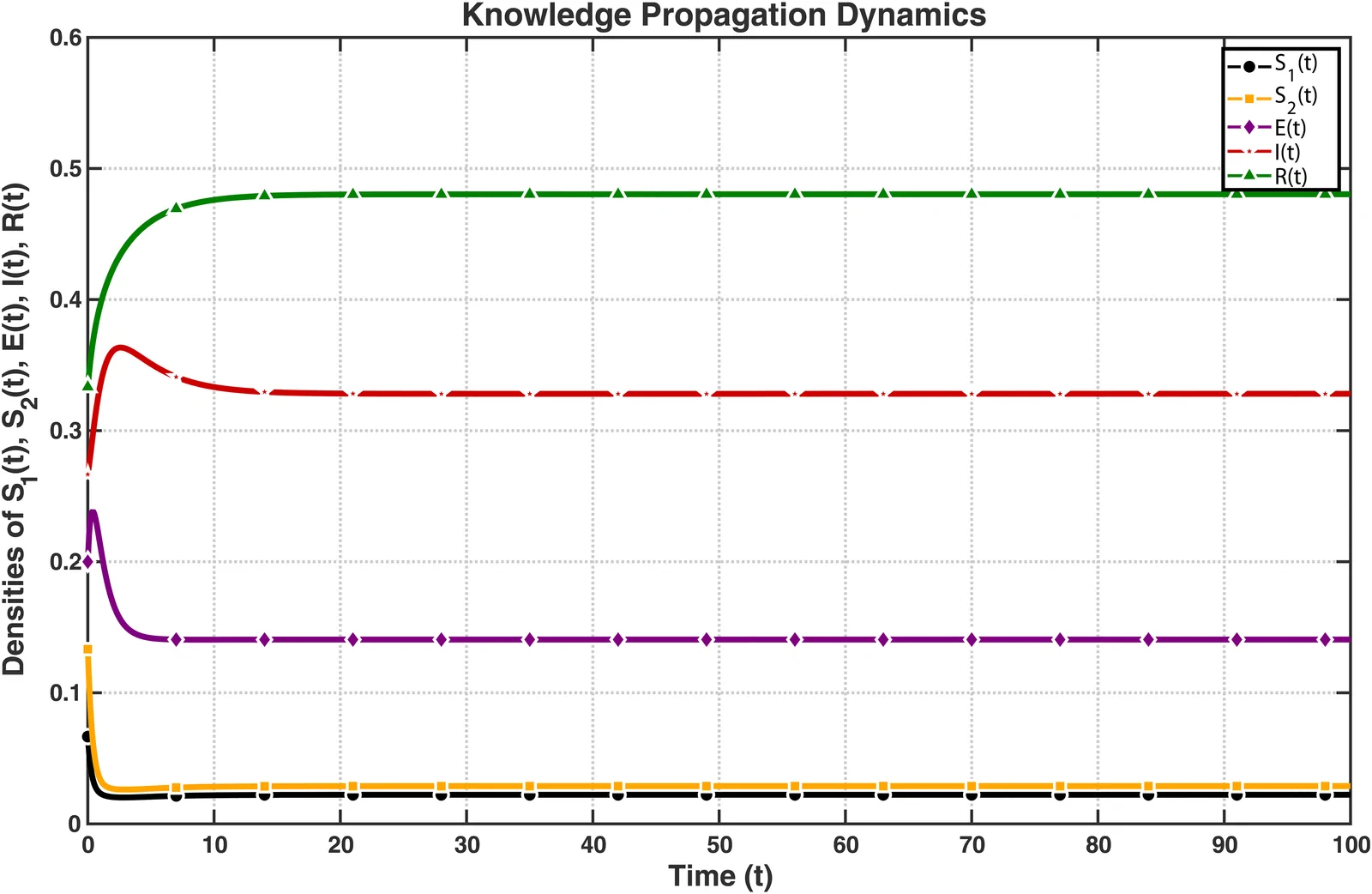
Stability of the equilibrium point $\Lambda_{1}$.

### Numerical simulations for parameter sensitivity analysis

To more intuitively observe the influence of key parameters in the model on knowledge dissemination, this study conducts a numerical sensitivity analysis of selected system parameters based on the basic reproduction number *R*_0_. By constructing three-dimensional response surfaces under two-dimensional parameter variations, the effects of different parameter combinations on *R*_0_ are systematically examined. Fig 6(a)–6(c) respectively present the dynamic characteristics of *R*_0_ under variations in the parameter pairs (α,η),(ι,ρ), and (σ,β).

**Fig 6:**
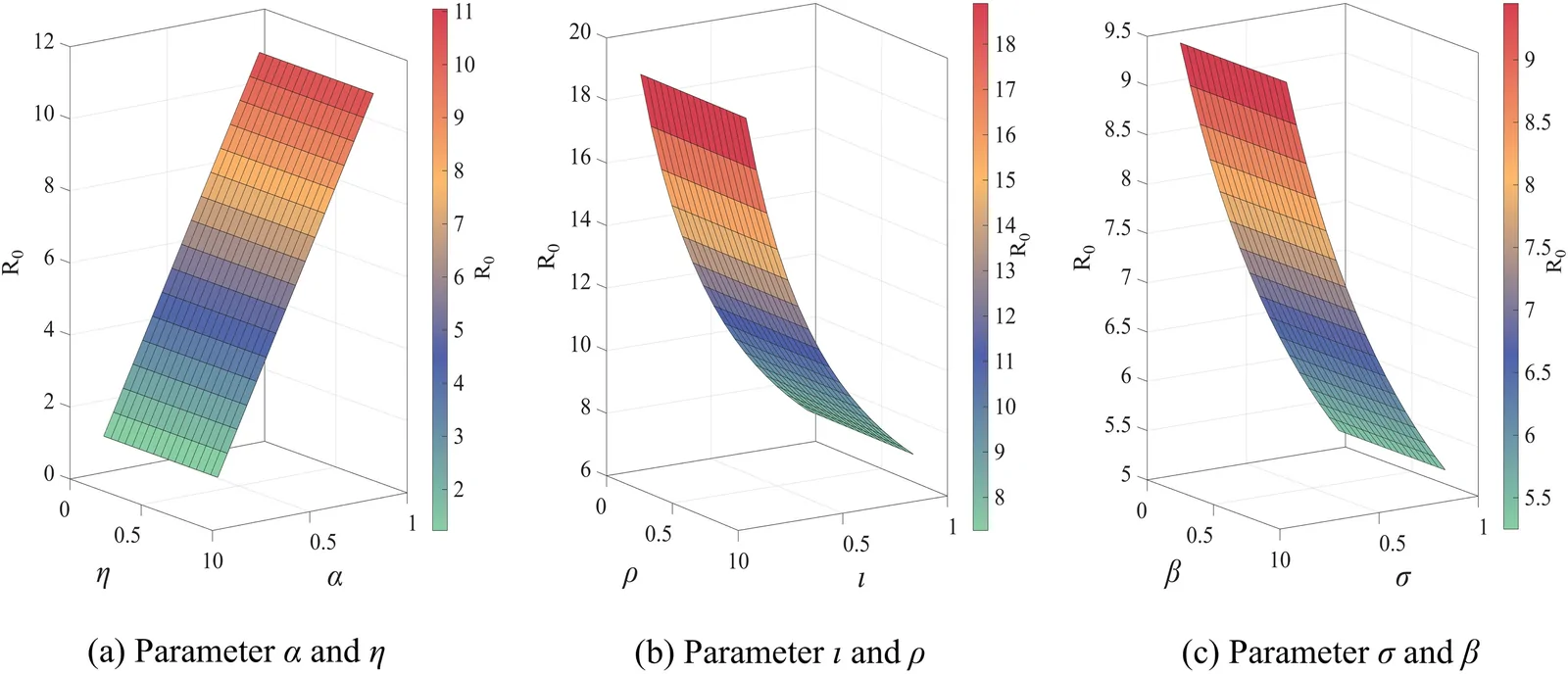
Parameter sensitivity analysis.

As shown in Fig 6, some parameters exert only a limited influence on the system within low-value intervals; however, once they exceed a certain threshold, they can markedly alter the dissemination state of the system. Specifically, the knowledge contact rate and knowledge absorption rate exhibit typical characteristics of “promoting parameters,” as their increase significantly enhances the dissemination capacity of the system. By contrast, the employee outflow rate and the proportion of employees who have not yet mastered the knowledge function as “inhibiting parameters,” reducing the dissemination efficiency of the system primarily by weakening the continuity of the dissemination chain. This indicates that enterprise knowledge dissemination is not a linear process. Instead, it is driven by coupled multi-parameter effects, where critical values of key parameters determine whether the system transitions from low to high dissemination states. Therefore, in practice, enterprises should monitor critical parameter thresholds and facilitate threshold crossing by enhancing contact and absorption efficiencies, thereby boosting overall diffusion capacity.

### Interaction analysis of key parameters

To reveal the influence of key parameters in the model on the structure of knowledge diffusion within enterprises, this section conducts parametric analysis on the variation in the proportion of different population groups corresponding to different parameter combinations under the steady-state condition of the system. By constructing three-dimensional response surfaces under two-dimensional parameter variations, this section focuses on examining how the interactions among the knowledge contact rate α, the knowledge absorption rate ι, the network average degree *k*, the transition rate from knowledge-disseminating employees to knowledge-exited employees β, the natural employee outflow rate μ, the proportion of newly entering enterprise members who have not yet mastered the knowledge *p*, and the transformation rate from employees with strong knowledge mastery to knowledge-exited employees σ influence the proportions of different populations. To ensure the interpretability of the simulation results, all parameters are varied within the following reasonable intervals: α,ι∈[0.3,1],k∈[0.5,3],β∈[0.05,0.3],μ∈[0.05,0.4],p∈[0.1,0.9],σ∈[0.05,0.5]. The remaining parameters are kept consistent with those used in the preceding numerical simulations, so as to maintain the system in a stable state of knowledge dissemination. The corresponding simulation results are shown in Figs 7–10.

**Fig 7:**
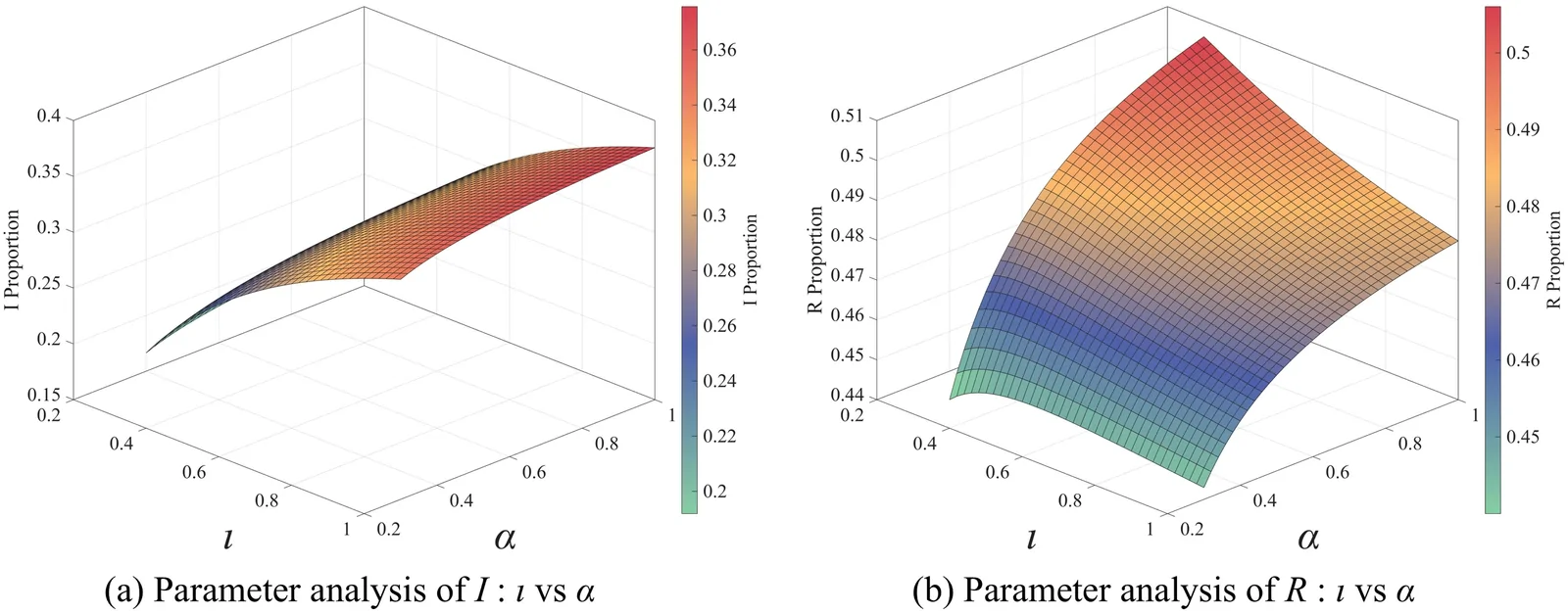
Parameters ι and α.

**Fig 8:**
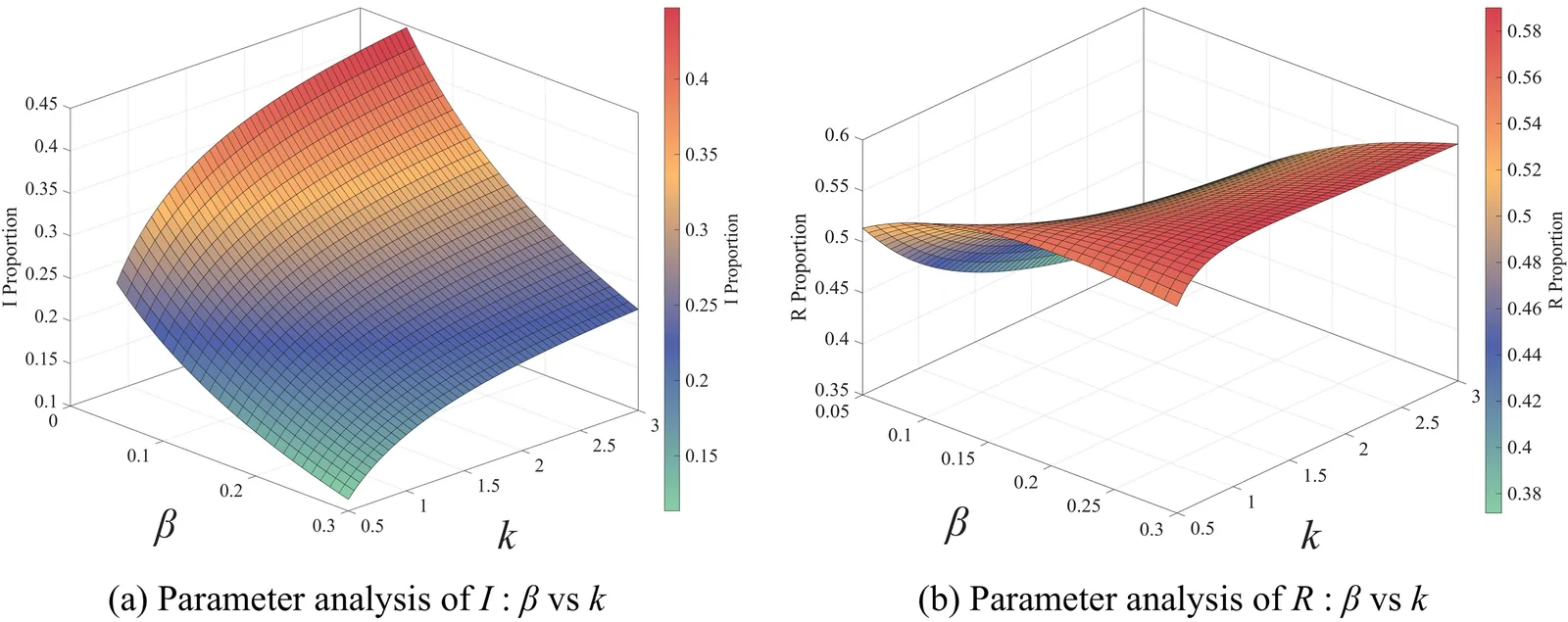
Parameter β and *k.*

**Fig 9:**
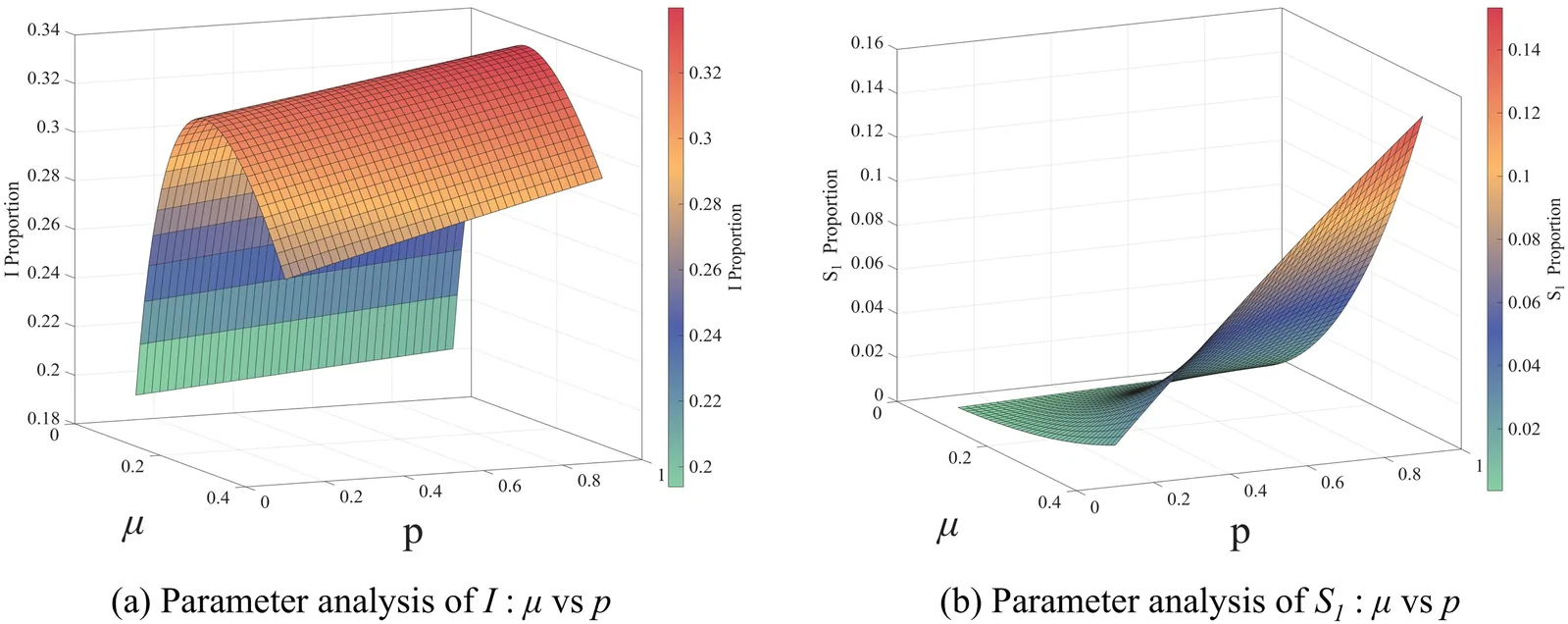
Parameter μ and *p.*

**Fig 10:**
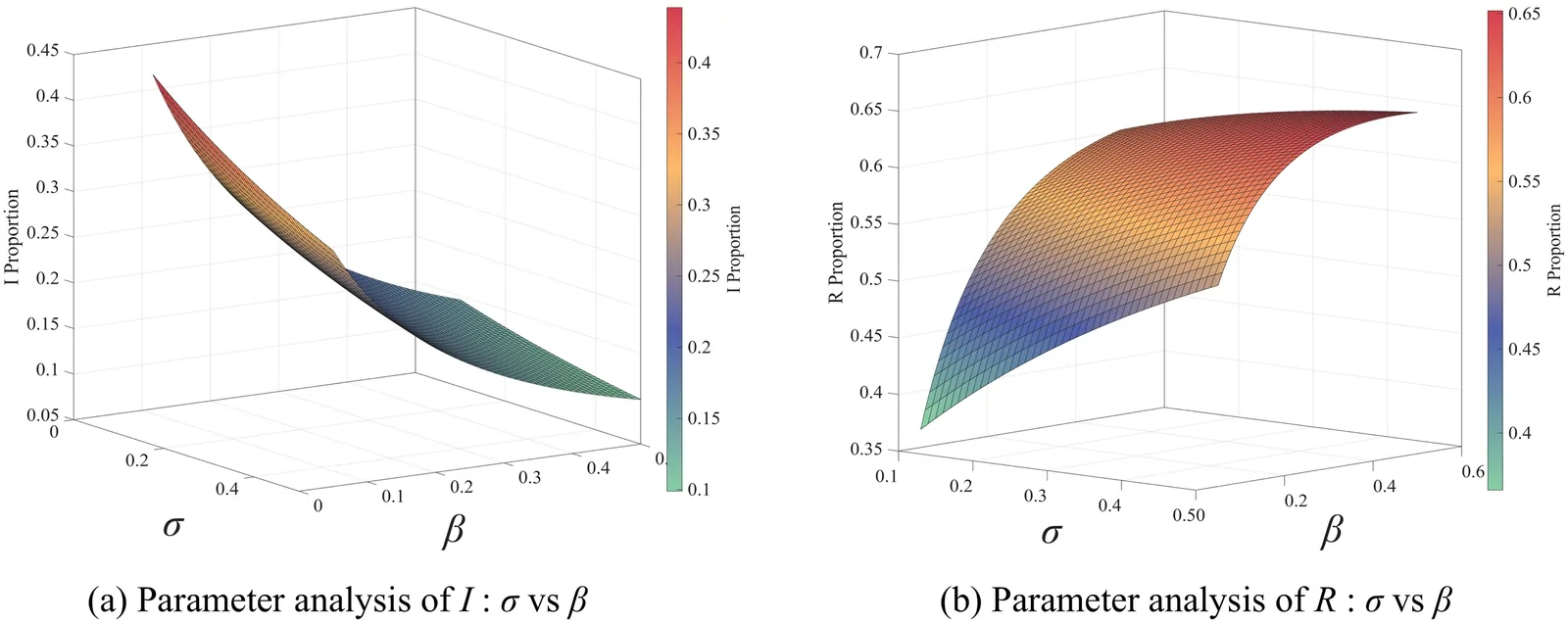
Parameter σ and β.

Fig 7(a) presents the joint influence of the knowledge contact rate α and the knowledge absorption rate ι on the proportion of knowledge-disseminating employees *I*. As illustrated in this figure, the proportion of knowledge-disseminating employees increases markedly as the two parameters rise simultaneously, while the overall variation remains relatively smooth. When either parameter is held fixed, increasing the other parameter can expand the scale of knowledge-disseminating employees, indicating that the two jointly promote dissemination through knowledge acquisition and transformation, respectively. However, in the high-parameter region, the growth rate of knowledge-disseminating employees gradually slows down, exhibiting a diminishing marginal effect. This suggests that, once contact and absorption capacities reach relatively high levels, their sustained promotional effect on dissemination scale becomes limited. Fig 7(b) presents the changing trend in the proportion of knowledge-exited employees under the same parameter combination. It can be observed that the proportion of knowledge-exited employees exhibits a pronounced upward trend as α increases, whereas the influence of ι on knowledge-exited employees is relatively weak, showing only a slight downward trend in the high-contact-rate region and, overall, displaying no significant monotonic pattern. Taken together, the formation of the knowledge-exited employee population mainly depends on the expansion of the knowledge dissemination scale, whereas the knowledge absorption rate influences the knowledge-exited employee population more indirectly by regulating the dissemination process, with a relatively limited marginal effect. This pattern is likewise applicable to knowledge dissemination in real organizational contexts: α corresponds to the information reach enabled by AI platforms and related mechanisms, whereas ι corresponds to employees’ capacity to internalize and assimilate knowledge into experience that can be further disseminated. If enterprises merely increase the frequency of knowledge push without establishing in-depth learning mechanisms, sustainable knowledge dissemination may be impeded. AI tools, by decomposing knowledge and constructing intelligent learning pathways, can enhance the knowledge absorption rate ι, facilitating the transformation of employees from knowledge recipients into knowledge disseminators and thereby fostering a sustainably diffusing knowledge dissemination network within the organization.

Fig 8(a) presents the joint influence of the network average degree *k* and the natural transformation rate from knowledge-disseminating employees to knowledge-exited employees β on the proportion of knowledge-disseminating employees *I*. From the variation of the response surface, it can be observed that the proportion of knowledge-disseminating employees increases with *k*, whereas it decreases significantly with β, indicating that the two parameters exert opposite effects on the knowledge-disseminating employee population. In the low-*k* region, the overall proportion of knowledge-disseminating employees remains relatively low and is highly sensitive to changes in β; as *k* increases, however, the proportion of knowledge-disseminating employees rises markedly, and the inhibitory effect of β exhibits a buffering characteristic. This suggests that high network connectivity can enhance the resistance of the knowledge-disseminating employee population to attrition. Fig 8(b) presents the changing trend in the proportion of knowledge-exited employees *R* under the same parameter combination. It can be observed that the proportion of knowledge-exited employees increases with both β and *k*, but its dependence on β is more pronounced. Taken together, the two figures indicate that the parameter β directly affects the population structure by regulating the dissemination and exit pathways, whereas the network average degree *k* indirectly modulates this process by enhancing the connectivity of dissemination pathways. When *k* is relatively high, the system is more likely to maintain a larger scale of knowledge-disseminating employees, thereby offsetting part of the attrition effect on knowledge-disseminating employees caused by an increase in β. The network average degree *k* corresponds to the density of collaboration among members within an enterprise organization, including cross-departmental communication, interactions in knowledge communities, and project-based collaborative relationships. Organizational closure may constrain the efficiency of knowledge dissemination, whereas a highly collaborative environment can enable disseminators to diffuse experience more rapidly. Therefore, enterprises can leverage AI-enabled collaborative platforms, knowledge communities, and cross-departmental project mechanisms to enhance the connectivity of the organizational network and construct a multi-node knowledge dissemination network.

Fig 9(a) presents the influence of the natural employee outflow rate μ and the proportion of employees among new system members who have not yet mastered the knowledge *p* on the knowledge-disseminating employee population *I*. It can be found that the proportion of knowledge-disseminating employees *I* declines significantly as the natural employee outflow rate μ increases, indicating that excessive personnel outflow weakens the continuity of knowledge dissemination. Meanwhile, when *p* is relatively high, the overall proportion of knowledge-disseminating employees remains low, suggesting that if new members in the organization are predominantly employees who have not yet mastered the knowledge, short-term dissemination efficiency will be inhibited to a certain extent. The interaction between the two parameters further indicates that the superposition of a high personnel outflow rate and a high proportion of employees who have not yet mastered the knowledge can substantially weaken the knowledge dissemination capacity within the enterprise. Fig 9(b) further illustrates the influence of the outflow rate μ and the proportion of employees who have not yet mastered the knowledge *p* on the population of employees who have not yet mastered the knowledge *S*_1_. As shown in the figure, the population *S*_1_ increases rapidly with the rise of *p*, while an increase in μ further amplifies this trend. This indicates that, in highly mobile organizations, if effective knowledge guidance mechanisms are lacking, new members may remain in a low-knowledge state for a prolonged period, thereby hindering knowledge diffusion. In enterprises undergoing rapid expansion or experiencing substantial personnel mobility, the large-scale entry of new employees may lead to a phased dilution of the overall knowledge level. If enterprises fail to establish a systematic knowledge guidance mechanism in a timely manner, new employees may find it difficult to integrate into the existing knowledge network, thereby reducing the efficiency of knowledge dissemination. Therefore, enterprises can leverage AI-based learning platforms to establish intelligent onboarding learning systems, enabling new employees to rapidly access key knowledge pathways and thereby shortening the transformation time from employees who have not yet mastered the knowledge *S*_1_ to knowledge-disseminating employees *I*.

Fig 10(a) presents the influence of the transformation rate from employees with strong knowledge mastery σ and the natural transformation rate of knowledge-disseminating employees β on the proportion of knowledge-disseminating employees *I*. As illustrated in this figure, as σ and β increase simultaneously, the proportion of knowledge-disseminating employees declines markedly, with the downward trend being more pronounced along the β direction. This indicates that, when both employees with strong knowledge mastery and knowledge-disseminating employees exit the dissemination process at relatively high rates, the effective dissemination agents in the system will rapidly decrease, thereby significantly weakening the capacity for knowledge diffusion. Correspondingly, Fig 10(b) shows the proportion of knowledge-exited employees *R* under the same parameter combination. It can be observed that the population *R* increases significantly as σ and β rise simultaneously. This result indicates that the synergistic increase in σ and β accelerates the transition of knowledge dissemination toward the knowledge sedimentation stage, causing the system to enter a stable but less active state more rapidly. This phenomenon can be understood as a process of attenuation in the driving force of knowledge dissemination: when knowledge-disseminating employees lack sustained incentives or perceived dissemination value, they will gradually cease their knowledge-sharing activities. If this phenomenon becomes widespread, the knowledge dissemination network may continue to deteriorate even when the organization possesses a large number of highly knowledgeable employees. Therefore, enterprises need not only to cultivate knowledge-disseminating employees, but also to maintain their activity through AI-driven incentive mechanisms, thereby preventing the knowledge network from prematurely entering a stable but low-activity state.

The above interaction analysis of parameters shows that the efficiency of knowledge dissemination within enterprises is not determined by a single parameter, but is jointly shaped by the synergistic effects of knowledge contact, knowledge absorption, organizational network structure, and individual transformation mechanisms. Among them, the synergistic enhancement of the knowledge contact rate α and the knowledge absorption rate ι helps expand the scale of knowledge-disseminating employees, while an increase in the network average degree *k* can effectively buffer the negative impact caused by the transition of knowledge-disseminating employees to the knowledge-exited state. By contrast, an excessively high natural employee outflow rate μ, together with the exit-related parameters β and σ, will significantly weaken the capacity for knowledge diffusion. The above results indicate that, in an AI-enabled context, enterprises should enhance opportunities for knowledge contact and improve knowledge absorption efficiency, optimize the organizational network structure, and at the same time reasonably control the pace of knowledge sedimentation and personnel mobility, so as to achieve a dynamic balance between knowledge dissemination efficiency and system stability.

### Validation under Different Combinations of Key Parameters

To examine whether the preceding conclusions depend on a single baseline parameter configuration, this study jointly investigates the effects of the knowledge contact rate α and the two AI-assisted learning transformation rates $\varphi_{1}^{AI}$ and $\varphi_{2}^{AI}$ on the peak number of knowledge-disseminating employees $I_{\max}$. In Fig 11(a), α∈[0.30,1.00] and $\varphi_{1}^{AI}\in \left[0.52,0.96\right]$, while $\varphi_{2}^{AI}=0.50$ is held fixed; in Fig 11(b), α∈[0.30,1.00] and $\varphi_{2}^{AI}\in \left[0.10,0.68\right]$, while $\varphi_{1}^{AI}=0.70$ is held fixed. All other parameters and initial conditions remain at their baseline settings, and all parameter combinations satisfy $\varphi_{1}^{AI}>\varphi_{2}^{AI}$.

**Fig 11:**
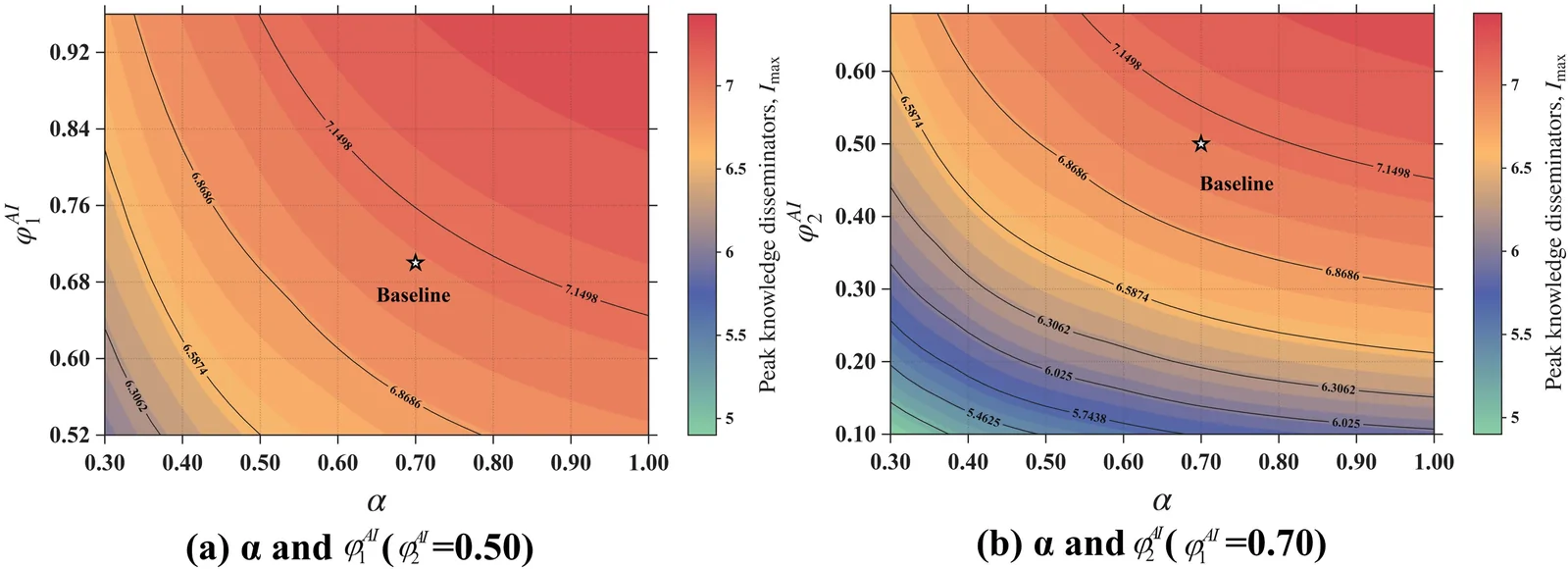
Validation under alternative combinations of key parameters.

The results show that $I_{\max}$ generally increases with the knowledge contact rate α and the two AI-assisted learning transformation rates, with the direction of these effects remaining consistent across different parameter combinations. The curved contour lines in both panels further indicate that the effect of AI-assisted learning varies with the level of knowledge contact, which is consistent with the multiplicative transition mechanism embedded in the model: interpersonal knowledge access and AI-enabled learning jointly shape employees’ transitions toward higher levels of knowledge mastery. The baseline parameter point lies within a relatively broad high-dissemination region, and variations in its neighboring parameter combinations do not alter the overall trend, indicating that the above conclusions are not driven by any single parameter configuration. Because the two AI-assisted learning transformation rates are examined over different parameter ranges, the purpose of this analysis is to verify the consistency of their effect directions rather than to compare their relative sensitivities.

### Deterministic–stochastic consistency check

To further verify the consistency of the numerical simulations, this study constructs a finite-population stochastic jump model with the same state-transition mechanisms as the deterministic five-compartment model. The stochastic model adopts the well-mixed assumption, with a system size of Ω=200 and a time step of 0.01, and each scenario is simulated independently for 100 realizations. The baseline scenario $\left(\alpha ,\varphi_{1}^{AI},\varphi_{2}^{AI},\iota )=(0.70,0.70,0.50,0.70\right)$ and a low-contact, low-learning-efficiency scenario (0.50,0.60,0.40,0.55) are examined separately, with the proportion of knowledge-disseminating employees, *I*(*t*)/*N*(*t*), used for comparison.

As shown in Fig 12, under both scenarios, the stochastic simulations closely reproduce the growth, peak, and steady-state convergence dynamics of the deterministic model. Under the baseline scenario, the RMSE, maximum absolute error, and Pearson correlation coefficient are 0.0012, 0.0035, and 0.9998, respectively; under the low-contact, low-learning-efficiency scenario, the corresponding values are 0.0008, 0.0021, and 0.9999. Moreover, the deterministic trajectory generally remains within the 95% interval of the stochastic simulations, while the residuals remain on the order of 10^–3^ without accumulating over time. The results indicate that the continuous model can robustly capture the mean dynamic behavior of the stochastic system governed by the same state-transition mechanisms, thereby supporting the internal consistency of the model’s numerical implementation. It should be emphasized that this assessment constitutes a numerical consistency validation rather than an independent validation of empirical validity.

**Fig 12:**
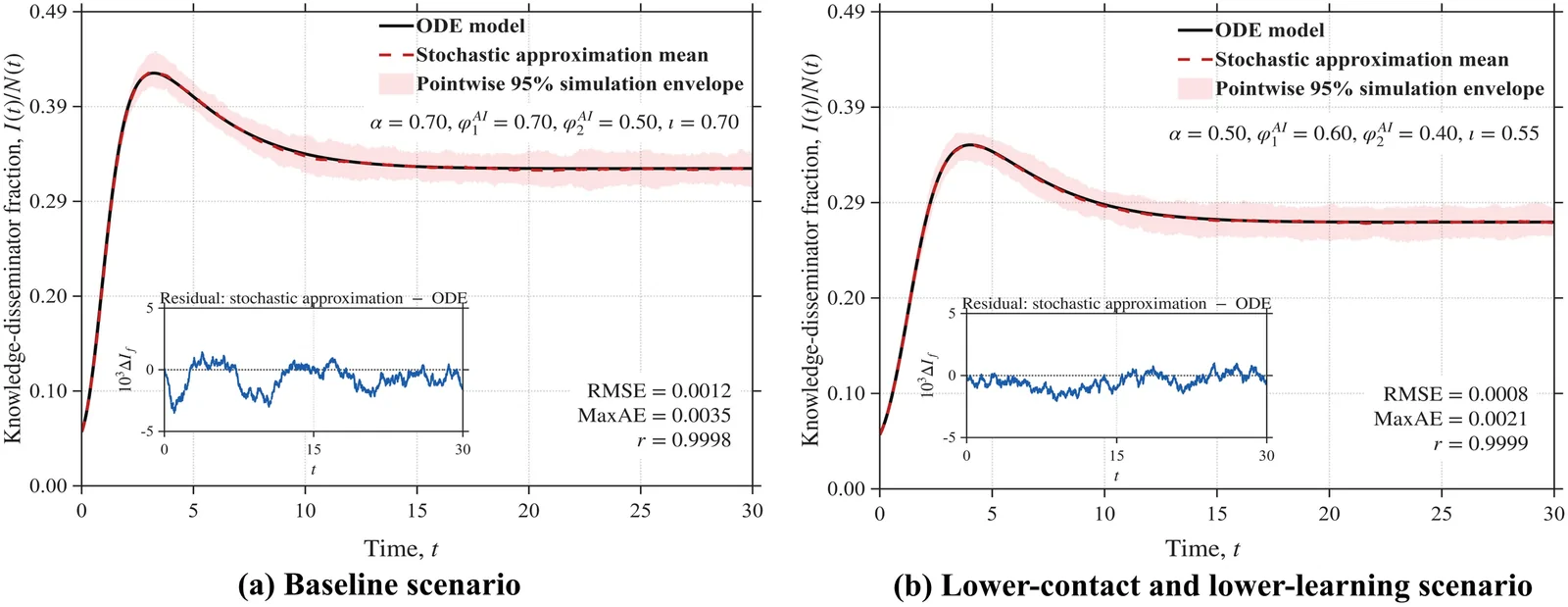
Deterministic–stochastic consistency check.

### Robustness analysis

To examine the robustness of the main simulation results with respect to parameter settings, initial conditions, and spatial simulation configurations, this study conducts a comprehensive robustness analysis from four perspectives: parameter perturbation, initial population composition, cellular automata (CA) grid size, and random seed selection. The corresponding results are presented in Fig 13. With all other parameters fixed at their baseline values, each of the 12 parameters in Table 2 was independently varied across its respective analysis range using 21 equally spaced values, resulting in a total of 252 single-parameter perturbation simulations. Fig 13(a) compares the median trajectories, central 95% intervals, and the trajectory obtained under the baseline parameter configuration. Although parameter variations alter the peak values and steady-state levels of the proportion of knowledge-disseminating employees, the fundamental dynamic pattern characterized by rapid growth, short-term peaking, and gradual convergence remains consistent. Fig 13(b) further compares four initial population configurations: the baseline state (10,5,2,1,0), the state without initial knowledge-disseminating employees (11,5,2,0,0), the state with a higher initial proportion of knowledge-disseminating employees (8,5,2,3,0), and the state with a stronger initial knowledge foundation (6,5,6,1,0). Different initial conditions primarily influence the early growth rate, peak magnitude, and time to peak; however, all scenarios eventually converge toward similar stable levels in the long term.

**Fig 13:**
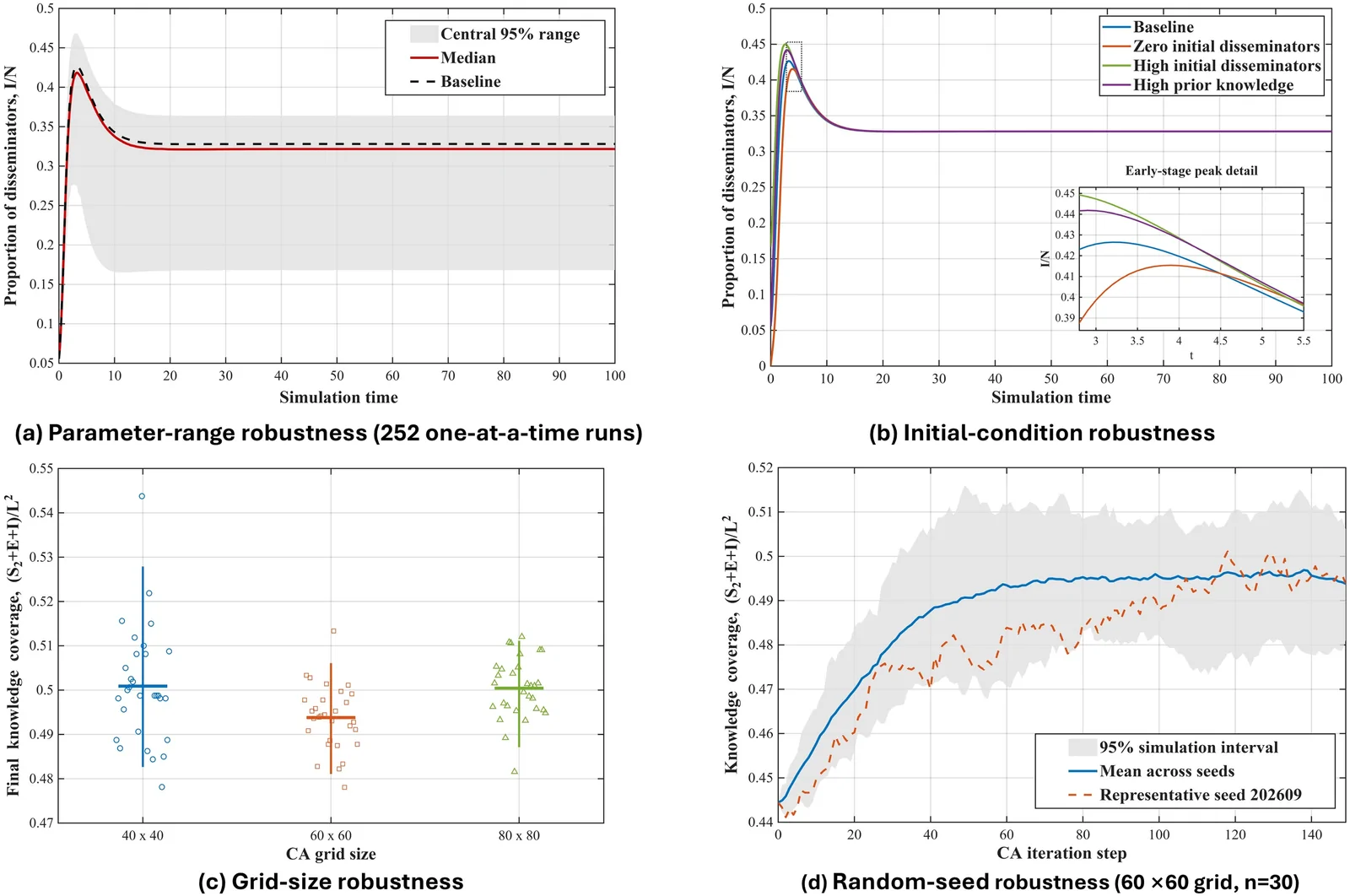
Robustness analysis of the continuous dynamical and cellular automata models.

For the spatial simulations, Fig 13(c) compares the terminal knowledge coverage rates obtained from 30 independent runs on three grid sizes: 40×40, 60×60, and 80×80. The results for the different grid sizes are concentrated around similar levels, while the stochastic dispersion decreases somewhat as the grid size increases, indicating that the normalized coverage rate exhibits no pronounced finite-size dependence. Fig 13(d) further presents the evolutionary trajectories from 30 random seeds on the baseline 60×60 grid. Although individual runs exhibit localized stochastic fluctuations, the overall growth trend, convergence process, and terminal coverage level remain highly consistent. Overall, the principal dynamic characteristics of the model remain stable under variations in parameter settings, initial conditions, grid size, and stochastic realizations, supporting the internal robustness of the simulation findings reported in this study.

### Simulation of optimal control strategies

To verify the regulatory effect of the optimal control strategy on knowledge dissemination within enterprises, this section conducts a numerical simulation study of the optimal control system based on the preceding theoretical analysis. Based on the preceding theoretical analysis of the optimal control strategy, the knowledge contact rate α(t), the AI-assisted learning transformation rates $\varphi_{1}^{AI}\left(t\right)$ and $\varphi_{2}^{AI}\left(t\right)$, and the knowledge absorption rate ι(t) are selected as control variables. To systematically evaluate the effectiveness of the optimal control strategy, this study establishes three scenarios for comparative analysis: no control, half control, and optimal control. The optimal control strategy is derived using Pontryagin’s Maximum Principle, and the state system and adjoint system are numerically solved using the Runge–Kutta algorithm to obtain the dynamic evolution curves of the system under different control scenarios. To explore strategies for promoting knowledge dissemination, this section focuses on the dynamic changes in the population of employees with strong knowledge mastery E(t) and the population of knowledge-disseminating employees I(t). In the process of enterprise knowledge dissemination, the *E* population represents the knowledge reserve layer, whereas the *I* population constitutes the true core force driving knowledge diffusion. Figs 14–17 respectively illustrate the dynamic evolution processes of the population of employees with strong knowledge mastery E(t) and the population of knowledge-disseminating employees I(t) under different control strategies.

**Fig 14:**
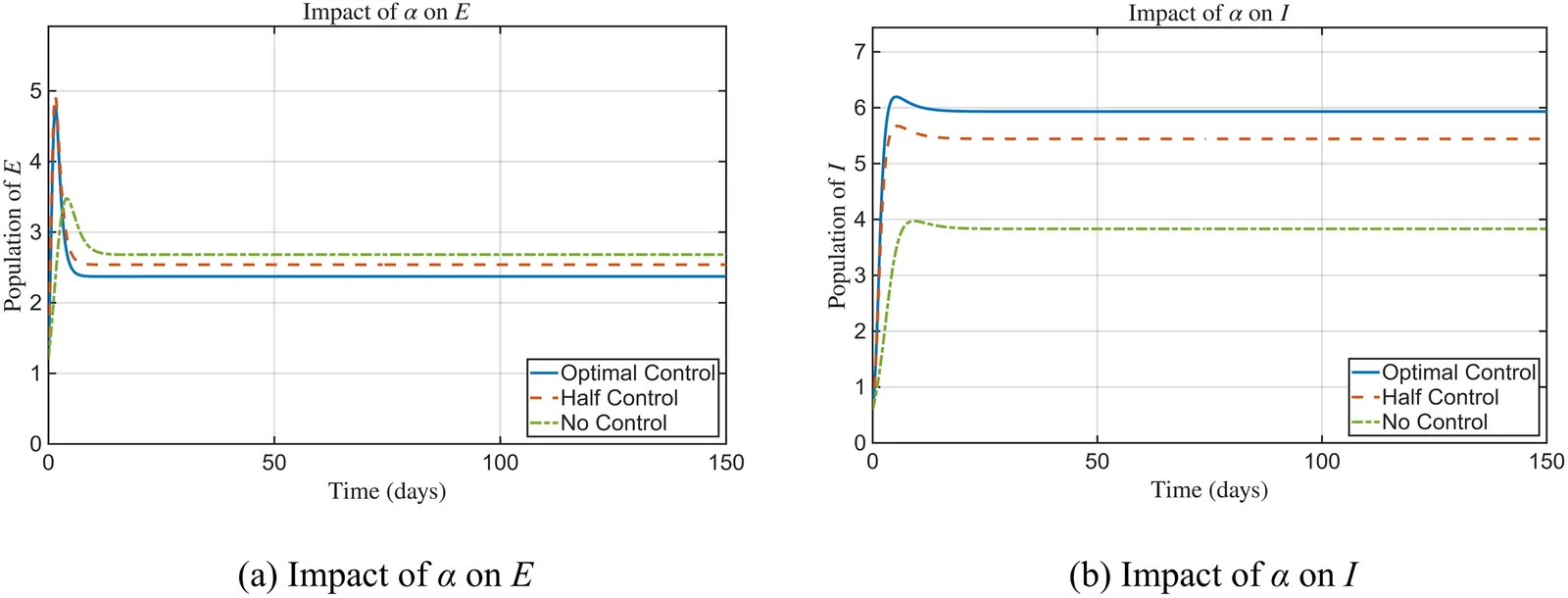
Influence of the knowledge contact rate α.

**Fig 15:**
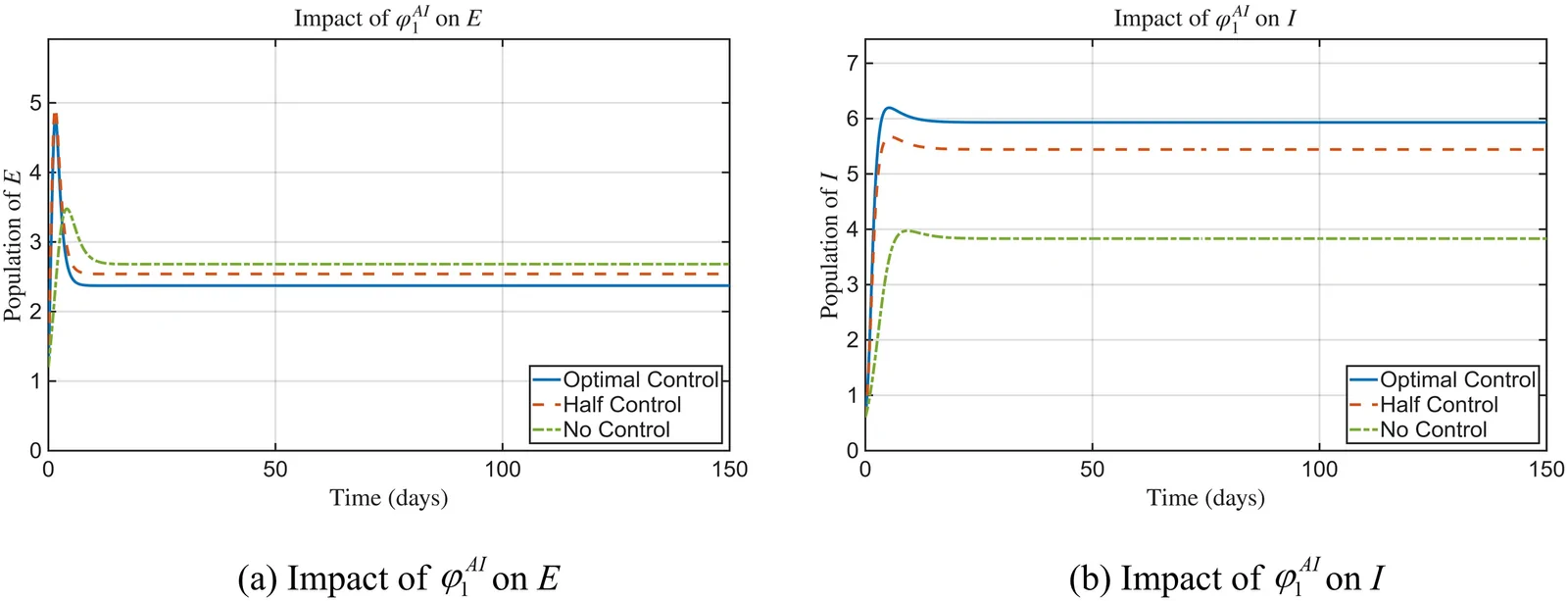
Influence of the AI-assisted learning transformation rate $\varphi_{1}^{AI}$.

**Fig 16:**
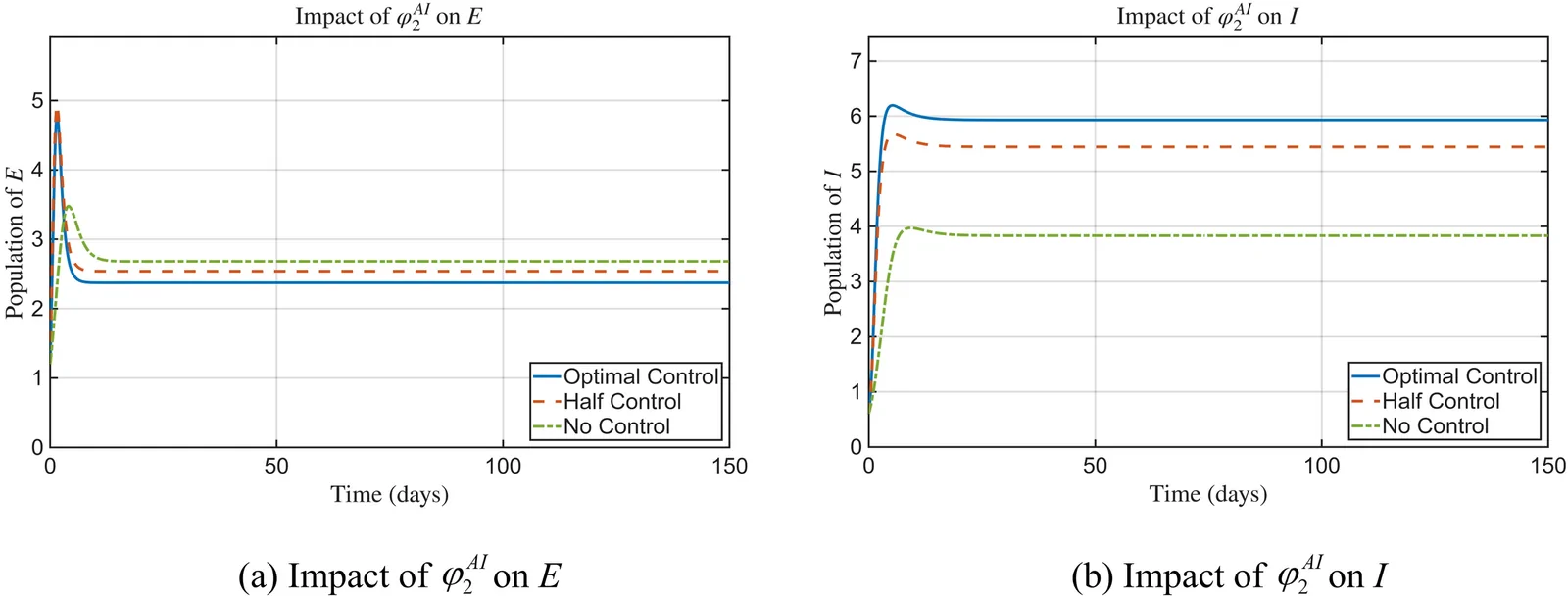
Influence of the AI-assisted learning transformation rate $\varphi_{2}^{AI}$.

**Fig 17:**
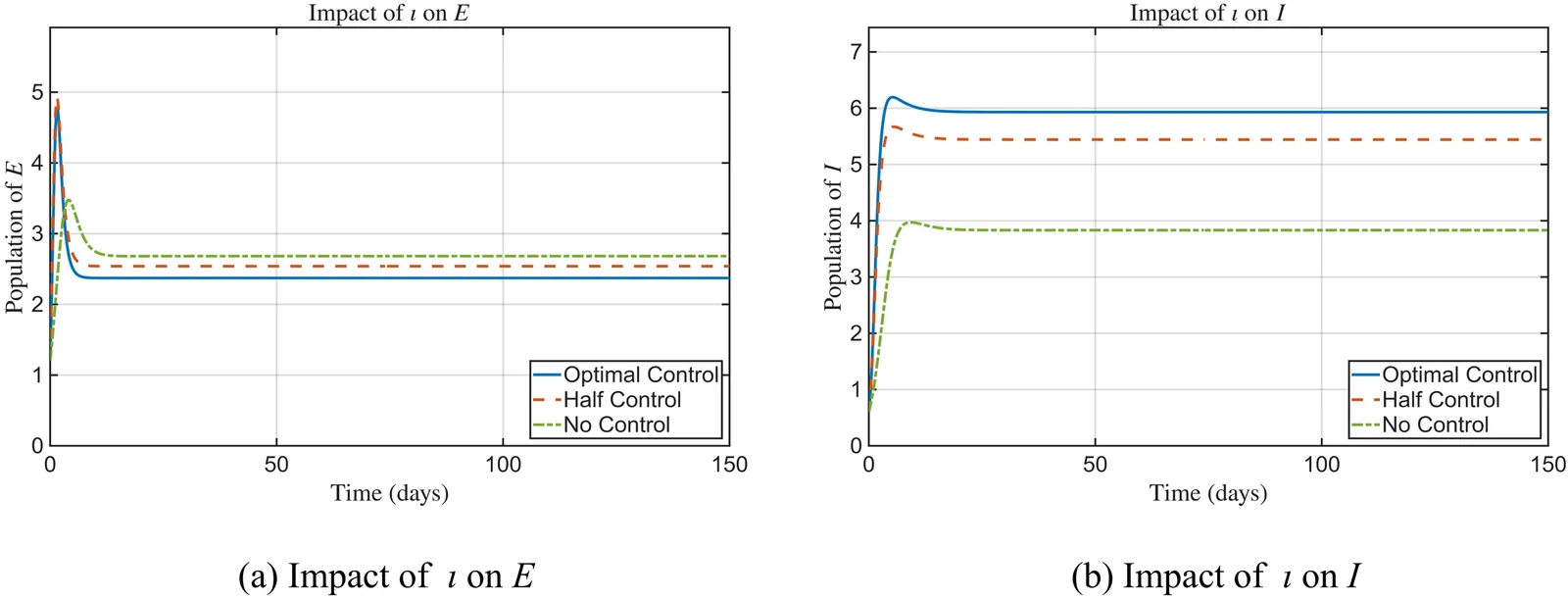
Influence of the knowledge absorption rate ι.

Fig 14(a) and 14(b) present the numerical simulation results for the evolution of E(t) and I(t), respectively, under the condition that optimal control is applied to the knowledge contact rate α(t). Let B=3,p=0.5,k=1.5,η=0.15,ρ=0.15,β=0.08,μ=0.2,σ=0.15,α∈[0.1,0.95]. The results show that the population of employees with strong knowledge mastery reaches a peak in the early stage and then declines under all scenarios, while optimal control significantly accelerates its transformation into knowledge-disseminating employees. Meanwhile, the population of knowledge-disseminating employees grows more rapidly and attains a higher steady-state level under optimal control, indicating that the control strategy can effectively improve the efficiency of knowledge dissemination. From the perspective of knowledge dissemination, the parameter α can be regarded as the opportunities for employees within an enterprise to come into contact with knowledge, such as the information reach enabled by internal AI knowledge platforms, intelligent question-answering recommendation systems, online collaborative communities, and cross-departmental project communication. When an organization lacks an effective mechanism for information reach, even if the enterprise already possesses a certain number of employees with strong knowledge mastery, such knowledge may remain confined to individuals or small-scale teams, making it difficult to achieve organization-level diffusion. By contrast, when AI-enabled knowledge management technologies are introduced, the knowledge contact rate α can be improved through measures such as pushing relevant knowledge to employees via intelligent recommendation algorithms, promoting cross-departmental communication through collaboration in knowledge communities, and helping employees rapidly identify domain experts through AI assistants. In this case, knowledge linkages among employees are continuously strengthened, and employees with strong knowledge mastery are more likely to transform into knowledge-disseminating employees, thereby gradually forming a spontaneously diffusing knowledge network within the enterprise.

Fig 15 presents the numerical simulation results for the evolution of the AI-assisted learning transformation rate $\varphi_{1}^{AI}\left(t\right)$ under different control scenarios. This parameter describes the transformation process from employees who have not yet mastered the knowledge *S*_1_ to employees with strong knowledge mastery *E*. Let $B=3,p=0.5,k=1.5,\eta =0.15,\rho =0.15,\beta =0.08,\mu =0.2,\sigma =0.15,\varphi_{1}^{AI}\in \left[0.1,0.95\right].$ As shown in Fig 15(a), under optimal control, the population of employees with strong knowledge mastery declines more rapidly and reaches a lower steady-state level, indicating that this strategy can shorten the time individuals remain in the knowledge mastery stage. As shown in Fig 15(b), the population of knowledge-disseminating employees reaches a higher steady-state level under optimal control, indicating that improving the AI-assisted learning transformation rate can significantly enhance the capacity for knowledge dissemination. This result reflects the critical role played by AI-assisted learning platforms in improving employees’ learning capabilities. Newly onboarded employees or employees with a relatively weak knowledge foundation often require a longer period of time to master professional knowledge. If systematic learning support or professional guidance is lacking, they may remain in the learning stage for a prolonged period and find it difficult to engage in knowledge dissemination. However, when enterprises introduce AI-assisted intelligent training systems, intelligent learning pathways, and related adaptive courses, new employees can establish their knowledge structures more rapidly, thereby shortening the transformation time from employees who have not yet mastered the knowledge to employees with strong knowledge mastery. As learning efficiency improves, some employees will gradually develop into knowledge-disseminating employees, thereby promoting the continuous diffusion of knowledge within the enterprise.

Fig 16 presents the results of the influence of the AI-assisted learning transformation rate $\varphi_{2}^{AI}\left(t\right)$ on system evolution. This parameter describes the transformation process from employees with limited knowledge mastery *S*_2_ to employees with strong knowledge mastery *E*. Let $B=3,p=0.5,k=1.5,\eta =0.15,\rho =0.15,\beta =0.08,\mu =0.2,\sigma =0.15,\varphi_{2}^{AI}\in \left[0.1,0.95\right].$ As shown in Fig 16, similar to the scenario of $\varphi_{1}^{AI}\left(t\right)$, under optimal control, the population of employees with strong knowledge mastery reaches a peak within a short period and then declines rapidly, whereas the population of knowledge-disseminating employees increases significantly and tends toward stability. This indicates that reasonably regulating this transformation rate can promote the transition of individuals from the intermediate stage to the dissemination stage, thereby improving the overall dissemination efficiency of the system. In the enterprise knowledge dissemination network, a large number of newly onboarded employees are typically in a state of limited knowledge mastery. They may have already acquired certain basic professional concepts, yet have not fully understood their application scenarios or practical implementation methods. In the absence of effective learning tools, these employees often find it difficult to further improve and may therefore remain in the transitional stage of knowledge for a prolonged period. The introduction of AI-assisted learning systems can significantly change this situation. AI-assisted learning systems can connect related knowledge points through knowledge graphs and help employees understand the relationships among different types of knowledge through case-based demonstrations and other approaches. Only when employees with limited knowledge mastery gradually overcome cognitive barriers and enter the stage of strong knowledge mastery are they more likely to participate in knowledge-sharing activities, thereby further expanding the knowledge dissemination network within the enterprise.

Fig 17(a) and 17(b) respectively present the evolution results of E(t) and I(t) after optimal control is applied to the knowledge absorption rate ι(t). Let B=3,p=0.5,k=1.5,η=0.15,ρ=0.15,β=0.08,μ=0.2,σ=0.15,ι∈[0.1,0.95]. As shown in Fig 17(a), under the regulation of the knowledge absorption rate ι(t), the steady-state level of E(t) under the optimal control scenario is significantly lower than those under the no-control and half-control scenarios. This indicates that the introduction of AI-enabled learning support does not simply accumulate highly knowledgeable individuals, but rather enables them to internalize and assimilate knowledge more rapidly and enter the dissemination role. Correspondingly, as shown in Fig 17(b), the growth rate of I(t) is markedly accelerated under optimal control, and its steady-state value consistently remains the highest among the three control scenarios, indicating that improving the knowledge absorption rate can accelerate individuals’ transition into the dissemination stage, thereby enhancing the capacity for knowledge diffusion. The parameter ι reflects enterprise employees’ capacity to understand and absorb knowledge. After employees come into contact with new professional knowledge, they generally need to undergo processes of comprehension, digestion, and practical application before the knowledge can truly be transformed into experience that can be shared and disseminated. If knowledge is difficult to understand or lacks a concrete practical environment, employees may remain for a long time in the stage of having mastered the knowledge but not yet applied it, thereby affecting the rate of knowledge dissemination within the enterprise. Conversely, if enterprises can help employees better understand knowledge by improving training systems, practical projects, or team collaboration mechanisms, employees’ speed of knowledge absorption will increase, making them more likely to transform into knowledge-disseminating employees. Therefore, improving the knowledge absorption rate ι can significantly shorten the transformation time from the knowledge mastery stage to the knowledge dissemination stage, thereby further enhancing the capacity for knowledge diffusion within the enterprise.

Based on the numerical simulation results in Figs 14–17, it can be found that, under the optimal control strategy, the steady-state level of the population of employees with strong knowledge mastery E(t) decreases compared with the no-control and half-control scenarios, whereas both the scale and stability of the population of knowledge-disseminating employees I(t) are significantly improved. This result indicates that optimal control does not weaken the accumulation of knowledge; rather, by accelerating the structural transitions among populations, it enables knowledge to be transformed more efficiently from individual mastery into organizational diffusion. The population of employees with strong knowledge mastery mainly serves as a transitional state within the system, and an increase in its scale is not directly equivalent to an improvement in dissemination efficiency. Optimal control enhances the dissemination capacity of the system by shortening the residence time in this stage and accelerating their transformation into knowledge-disseminating employees. In practice, the maximization of knowledge value lies not in the static accumulation of individuals with high levels of knowledge mastery, but in whether such knowledge can be continuously transformed into a stable and efficient dissemination force. This study further considers knowledge dissemination within enterprises in an AI-enabled context, promoting knowledge dissemination through AI-enabled digital technologies. The optimal control strategy achieves the unity of knowledge dissemination efficiency and system stability within enterprises precisely through the coordinated regulation of multi-stage parameters.

### Analysis of spatiotemporal evolution characteristics under different control strategies

To illustrate the evolutionary patterns of enterprise knowledge dissemination in the spatiotemporal dimension, this section introduces the cellular automata method based on the preceding numerical analysis of optimal control, and simulates the dynamic evolution process of the knowledge dissemination system within the spatiotemporal network. The cellular automata model can characterize the spatiotemporal diffusion characteristics of collective behavior through local interaction rules among individuals, and is therefore commonly used to investigate the spatiotemporal structural evolution of complex dissemination systems. In this section, organizational members within the enterprise are mapped onto a 50×50 two-dimensional regular grid, where each grid point represents an enterprise member. The state of each member in the system evolves among five states according to the dissemination mechanisms specified in the model: employees who have not yet mastered the knowledge *S*_1_, employees with limited knowledge mastery *S*_2_, employees with strong knowledge mastery *E*, knowledge-disseminating employees *I*, and knowledge-exited employees *R*. The system adopts a Moore neighborhood structure, in which each individual interacts with the eight surrounding neighboring nodes. At each discrete time step, individual states are synchronously updated according to the transition probabilities specified in the model, and the updating rules are determined by the 2*SEIR*-*CA* dissemination mechanism constructed in this study. The initial state of the system is randomly distributed in the spatiotemporal domain, and the spatiotemporal evolution process of the system is recorded at multiple time nodes, namely *t* = 1,*t* = 30,*t* = 60,*t* = 100,*t* = 150. By comparing *t*he *t*hree scenarios of no con*t*rol, half con*t*rol, and optimal control, the influence of different control parameters on the spatiotemporal structure of the knowledge dissemination network within the enterprise can be directly observed. The four sets of simulation results respectively present the spatiotemporal evolution of the system under different control conditions for the knowledge contact rate α(t), the AI-assisted learning transformation rates $\varphi_{1}^{AI}\left(t\right)and\varphi_{2}^{AI}\left(t\right)$, and the knowledge absorption rate ι(t). It should be noted that the cellular automata analysis serves a different purpose from the stochastic consistency check presented in Fig 12. The former incorporates local neighborhood interactions and spatiotemporal heterogeneity to characterize the spatiotemporal evolution of knowledge dissemination, whereas the latter employs a well-mixed finite-population stochastic jump process to verify its consistency with the continuous five-compartment dynamical model.

Fig 18 presents the influence of the knowledge contact rate α(t) on the spatiotemporal structure of knowledge dissemination community evolution under different control scenarios. In the no-control scenario, knowledge-disseminating employees form only scattered clusters in local regions, and the overall spatiotemporal structure remains relatively dispersed. In the half-control scenario, the coverage of knowledge-disseminating employees expands to some extent, but their distribution remains unstable. Under optimal control, however, knowledge-disseminating employees can form a dominant spatiotemporal distribution within a relatively short period and eventually cover the entire network. This indicates that increasing the knowledge contact rate can significantly enhance the spatiotemporal diffusion capacity of knowledge dissemination. The knowledge contact rate reflects the frequency of information exchange among employees. When communication channels within the organization become more open and knowledge exchange among employees occurs more frequently, knowledge can diffuse more readily across different teams, thereby forming a more closely connected knowledge network structure.

**Fig 18:**
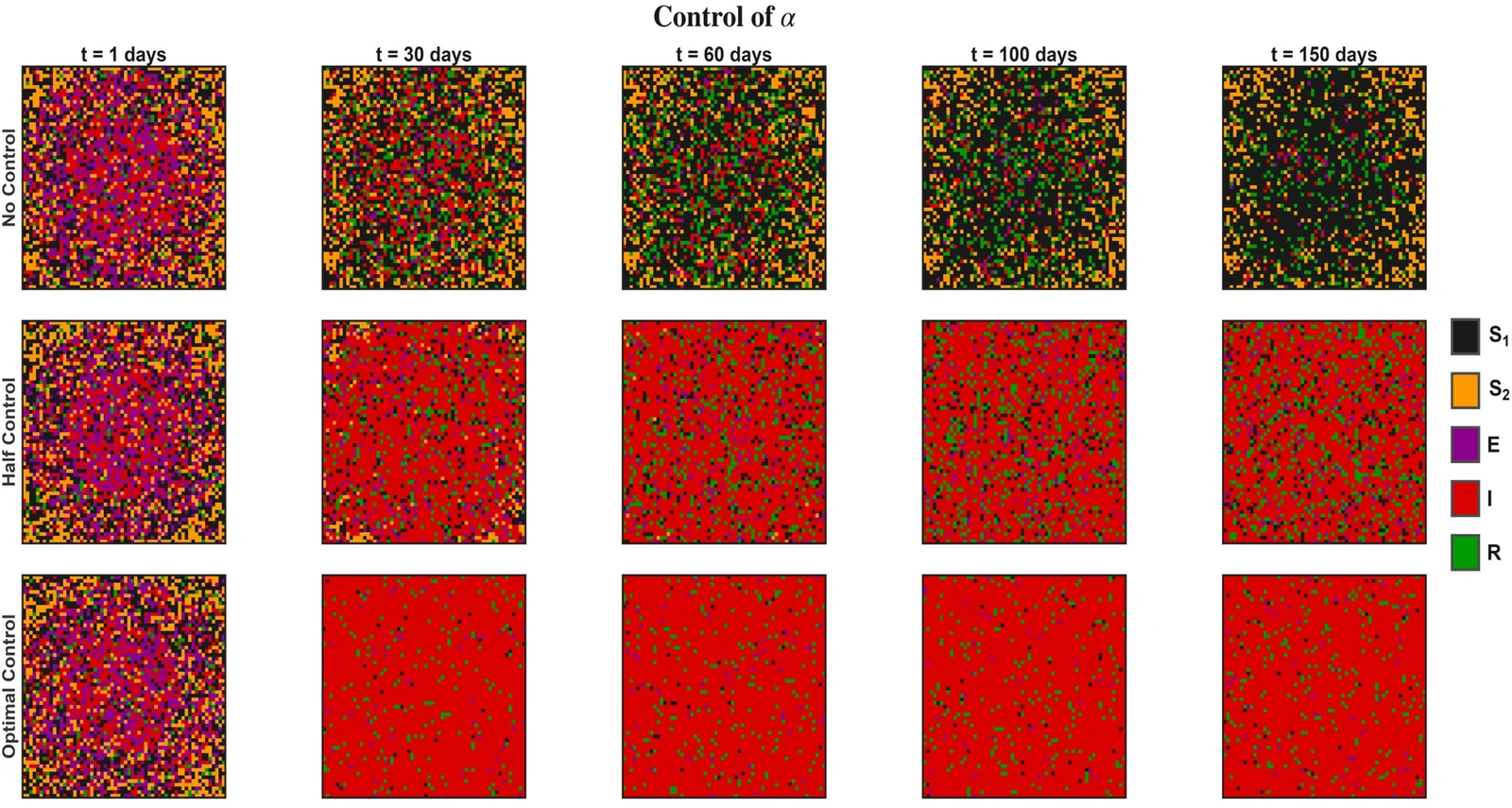
Spatiotemporal evolution of the control variable α.

Fig 19 presents the spatiotemporal evolution characteristics of the AI-assisted learning transformation rate $\varphi_{1}^{AI}\left(t\right)$ under different control strategies. This parameter describes the process by which employees who have not yet mastered the knowledge *S*_1_ transform into employees with strong knowledge mastery *E* through AI-assisted learning. It can be observed that, under the no-control condition, the population of employees with strong knowledge mastery persists in the spatiotemporal domain for a long time, while the distribution of knowledge-disseminating employees remains relatively limited. Under optimal control, however, the population of employees with strong knowledge mastery decreases rapidly, and knowledge-disseminating employees gradually become dominant. This indicates that optimizing this transformation process can effectively reduce knowledge retention and promote spatiotemporal diffusion. This process reflects the influence of AI-assisted learning efficiency among employees who have not yet mastered the knowledge on knowledge diffusion. When newly onboarded employees are able to rapidly establish their knowledge structures through systematic learning, they are also more likely to participate in knowledge-sharing activities and gradually become knowledge dissemination nodes within the organization.

**Fig 19:**
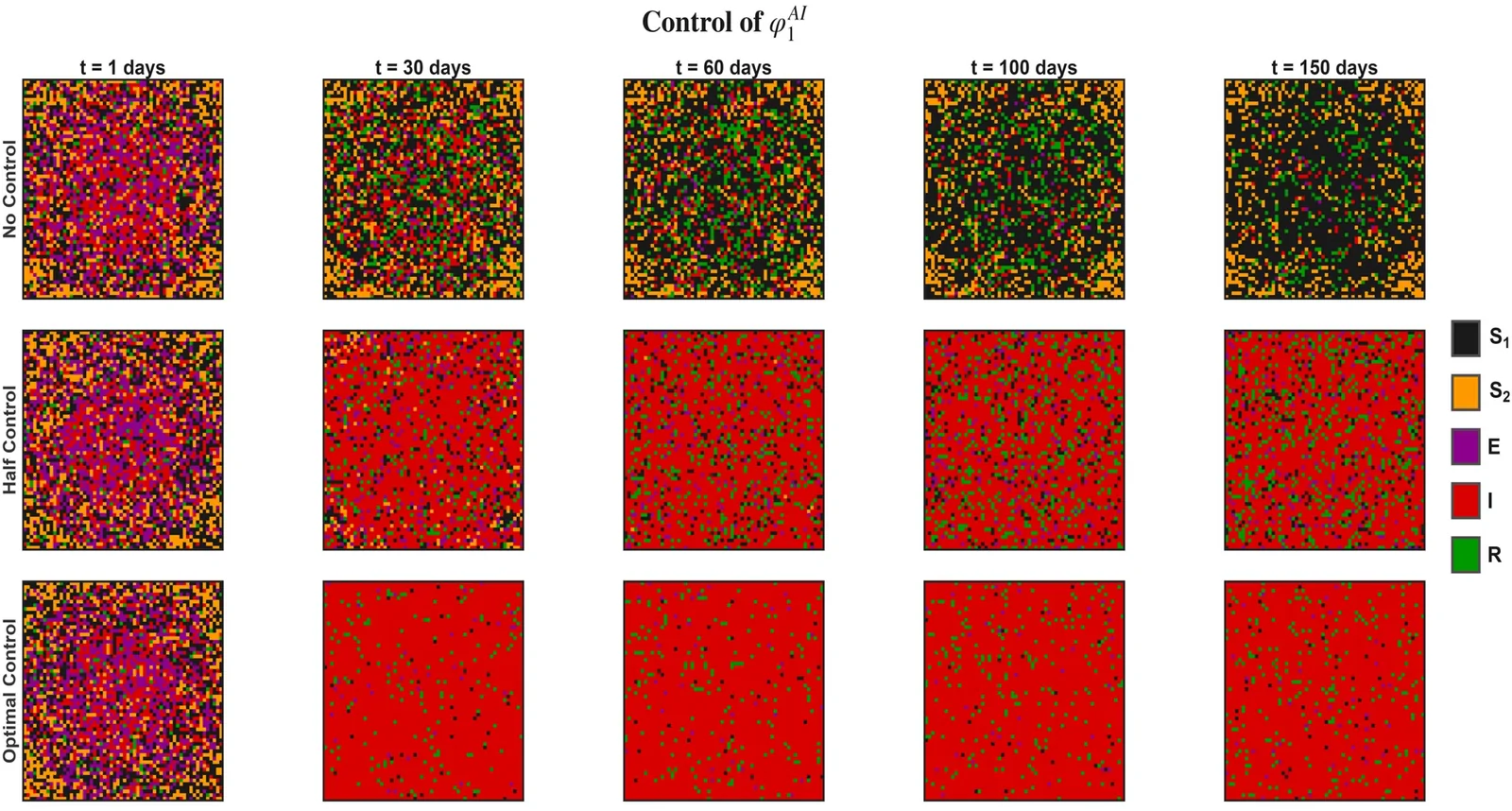
Spatiotemporal evolution of the control variable $\varphi_{1}^{AI}$.

Fig 20 presents the influence of the AI-assisted learning transformation rate $\varphi_{2}^{AI}\left(t\right)$ on the spatiotemporal structure of the system. This parameter describes the process by which employees with limited knowledge mastery *S*_2_ transform into employees with strong knowledge mastery *E* through AI-assisted learning. Under the optimal control scenario, the spatiotemporal retention of employees with strong knowledge mastery is significantly weakened, while the population of knowledge-disseminating employees expands and forms continuously distributed regions. This indicates that accelerating the transformation of individuals in the transitional stage helps improve the overall dissemination efficiency. Similarly, this process reflects the influence of AI-assisted learning efficiency among employees with a certain knowledge foundation on knowledge diffusion. As employees continuously improve their existing knowledge and expand their application capabilities, they are more likely to transform from knowledge learners into knowledge-disseminating employees.

**Fig 20:**
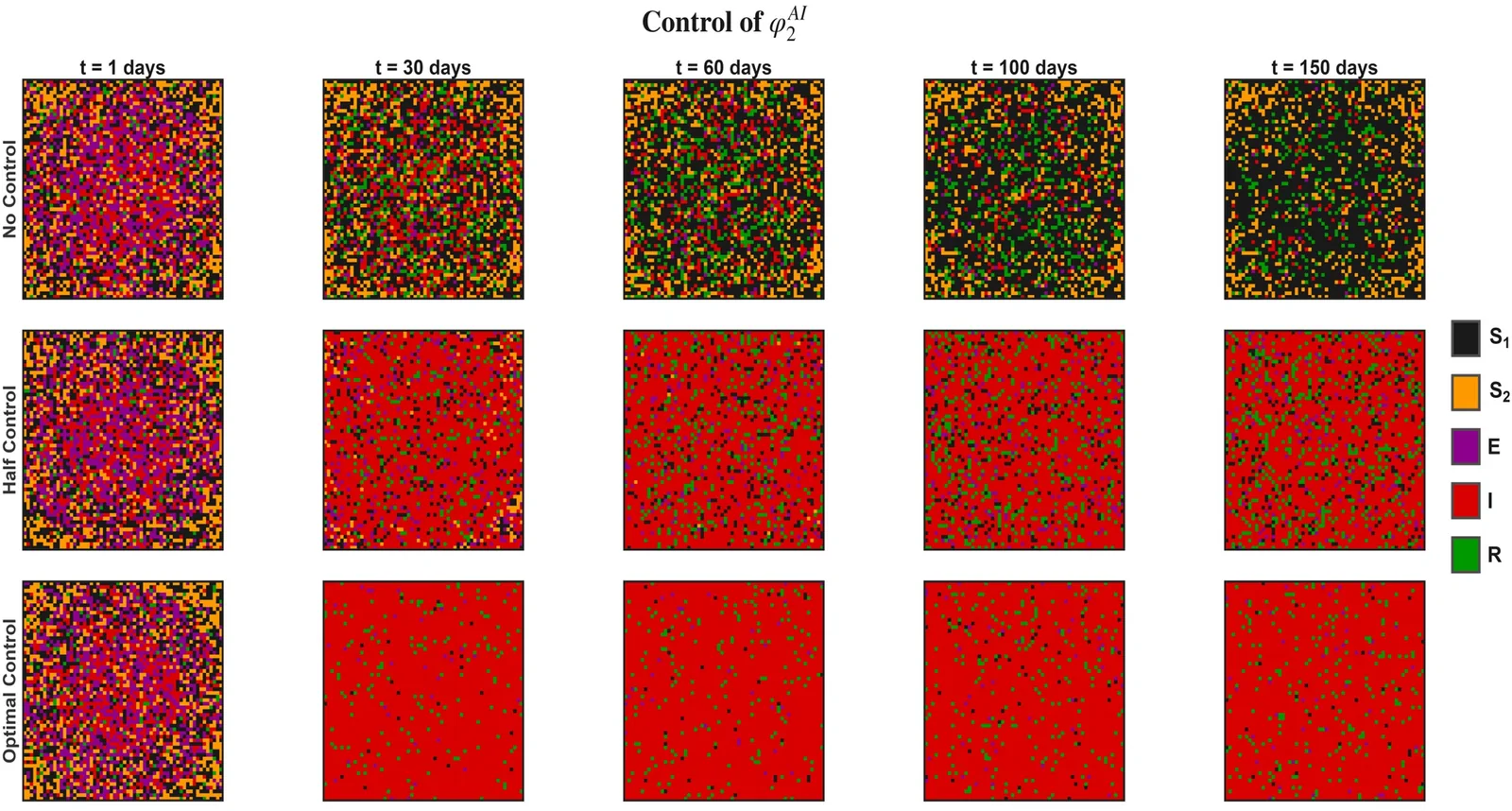
Spatiotemporal evolution of the control variable $\varphi_{2}^{AI}$.

Fig 21 presents the spatiotemporal evolution results of the knowledge absorption rate ι(t) under different control strategies. Under the no-control condition, although knowledge-disseminating employees *I* form small-scale clusters in local regions, their overall distribution remains relatively scattered, making it difficult to form a stable dissemination structure. Under optimal control, the population of knowledge-disseminating employees rapidly becomes dominant across the entire spatiotemporal domain and exhibits a highly consistent spatiotemporal distribution in the middle and later stages. This result indicates that optimal regulation of the knowledge absorption rate can effectively shorten the time individuals spend in the knowledge digestion stage, enabling them to transform more rapidly into actual knowledge-disseminating employees. The knowledge absorption rate reflects employees’ ability to absorb, master, and apply knowledge. When employees can rapidly understand the knowledge they have learned and apply it to practical work, they are more likely to promote knowledge diffusion within the organization through experience sharing.

**Fig 21:**
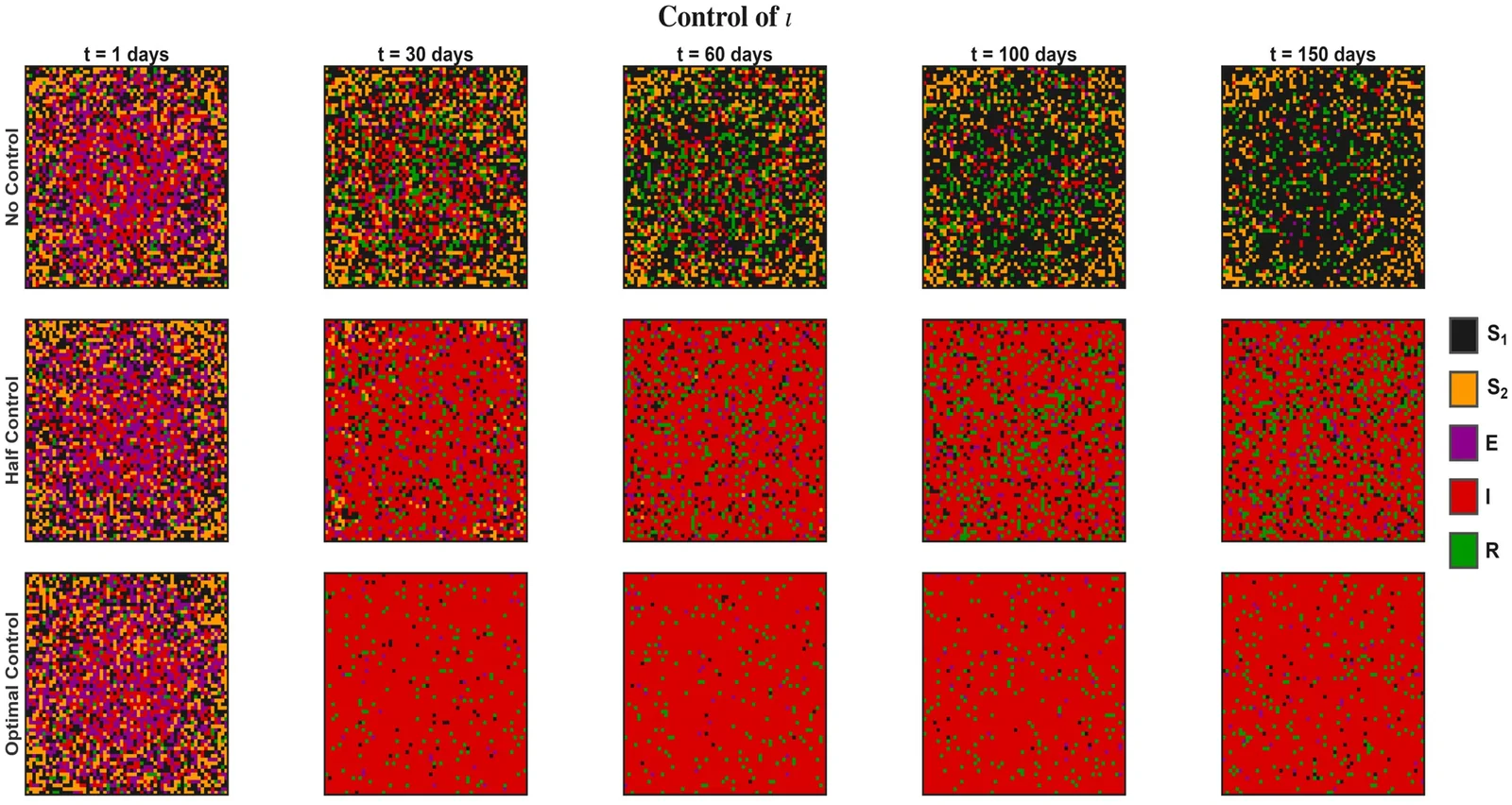
Spatiotemporal evolution of the control variable ι.

Taken together, the spatiotemporal evolution results shown in Figs 18–21 reveal that the optimal control strategy exhibits a highly consistent pattern at the spatiotemporal level: the system gradually evolves from an initially mixed multi-state distribution into a stable spatiotemporal configuration dominated by knowledge-disseminating employees *I*. This result is consistent with the preceding numerical simulation findings, further verifying the mechanism by which the optimal control strategy enhances the efficiency of knowledge diffusion within enterprises by accelerating knowledge-stage transitions and strengthening the formation of dissemination agents. This implies that, when enterprise organizations are able to simultaneously optimize knowledge contact, learning transformation, and knowledge absorption, knowledge dissemination will no longer remain confined to individual teams within the enterprise, but can instead form a stable and sustained diffusion structure across the entire organizational network.

## Conclusion and implications

Against the backdrop of artificial intelligence being deeply embedded in enterprise knowledge management and organizational learning processes, traditional knowledge dissemination models, which are founded on single interpersonal interaction and homogeneous mixing assumptions, cannot fully characterize the multi-stage dynamic mechanism of knowledge dissemination and its spatiotemporal evolution characteristics under “interpersonal–human-machine” collaboration. Based on this consideration, and taking into account differences in enterprise employees’ knowledge mastery as well as the AI-assisted learning mechanism, this paper constructs a multi-stage 2*SEIR*-*CA* dynamic model of knowledge dissemination that integrates an ‘interpersonal dissemination–human-machine interaction’ dual-channel structure. By introducing the cellular automata method, the macroscopic continuous dynamic model is further mapped onto a two-dimensional discrete spatiotemporal domain, so as to characterize the spatiotemporal evolution process driven by local interactions among individuals. On this basis, by integrating basic reproduction number analysis, equilibrium stability analysis, and optimal control theory, this study systematically investigates the dynamic mechanism, regulatory pathways, and spatiotemporal diffusion characteristics of knowledge dissemination within enterprises under the AI-enabled context. Under the above model and analytical framework, the system behavior exhibits the following characteristics:

1. Regarding the AI-enabled mechanism, under the assumed model structure, AI-assisted learning facilitates knowledge-state transitions, resulting in a multi-stage dissemination process jointly driven by interpersonal dissemination and human-machine augmentation.
2. Regarding parameter effects, within the examined parameter ranges, knowledge dissemination exhibits clear threshold-dependent behavior. Higher knowledge-contact and knowledge-absorption rates generally favor diffusion, whereas higher employee-outflow and knowledge-exit rates tend to weaken dissemination continuity. Additional parameter-perturbation and stochastic analyses indicate that these dynamic patterns are internally robust.
3. Regarding the optimal control strategy, under the assumed objective function, control constraints, and cost structure, the simulations suggest that dynamically coordinating knowledge contact, AI-assisted learning, and knowledge absorption may improve transition pathways across knowledge stages and the trade-off between dissemination benefits and intervention costs.
4. Regarding spatiotemporal evolution, under the specified local interaction rules, the model exhibits a transition from discrete clustering to continuous diffusion and stable coverage. Results across different CA grid sizes and stochastic realizations further support the internal stability of this simulated pattern.

These managerial implications should be interpreted in light of the assumptions of the proposed model rather than as universally applicable managerial prescriptions. Under the specified parameter and resource constraints, the model suggests that organizations may focus on knowledge contact, AI-assisted learning, knowledge absorption, and incentives for knowledge sharing, while dynamically adjusting intervention priorities across different dissemination stages. In particular, the optimal control trajectories are better interpreted as indicating the relative priority and intensity of different intervention channels rather than fixed resource-allocation proportions. Their practical application therefore requires adaptation to organization-specific conditions, such as AI-platform capacity, personnel availability, training resources, and managerial budgets.

Although the parameter perturbation, stochastic simulations, and alternative CA settings support the internal robustness of the model, the current study has not been empirically calibrated or externally validated using real enterprise data. Therefore, the findings should primarily be interpreted as theoretical and simulation-based patterns under the specified assumptions and parameter ranges. Future research could calibrate the model with longitudinal enterprise data and evaluate predictions across independent organizational samples, while incorporating complex network structures, individual heterogeneity, and dynamic recommendation mechanisms.

## Supporting information

S1 File Code. (ZIP)
